# Identification of microRNA Genes in Three Opisthorchiids

**DOI:** 10.1371/journal.pntd.0003680

**Published:** 2015-04-21

**Authors:** Vladimir Y. Ovchinnikov, Dmitry A. Afonnikov, Gennady V. Vasiliev, Elena V. Kashina, Banchob Sripa, Viacheslav A. Mordvinov, Alexey V. Katokhin

**Affiliations:** 1 Department of Human and Animal Genetics, Institute of Cytology and Genetics, Novosibirsk, Russian Federation; 2 Department of System Biology, Institute of Cytology and Genetics, Novosibirsk, Russian Federation; 3 Department of Natural Science, Novosibirsk State University, Novosibirsk, Russian Federation; 4 Sector of Genomic Investigation, Institute of Cytology and Genetics, Novosibirsk, Russian Federation; 5 Department of Pathology, Faculty of Medicine, Khon Kaen University, Muang District, Khon Kaen, Thailand; University of Melbourne, AUSTRALIA

## Abstract

**Background:**

*Opisthorchis felineus*, *O*. *viverrini*, and *Clonorchis sinensis* (family Opisthorchiidae) are parasitic flatworms that pose a serious threat to humans in some countries and cause opisthorchiasis/clonorchiasis. Chronic disease may lead to a risk of carcinogenesis in the biliary ducts. MicroRNAs (miRNAs) are small noncoding RNAs that control gene expression at post-transcriptional level and are implicated in the regulation of various cellular processes during the parasite- host interplay. However, to date, the miRNAs of opisthorchiid flukes, in particular those essential for maintaining their complex biology and parasitic mode of existence, have not been satisfactorily described.

**Methodology/Principal Findings:**

Using a SOLiD deep sequencing-bioinformatic approach, we identified 43 novel and 18 conserved miRNAs for *O*. *felineus* (miracidia, metacercariae and adult worms), 20 novel and 16 conserved miRNAs for *O*. *viverrini* (adult worms), and 33 novel and 18 conserved miRNAs for *C*. *sinensis* (adult worms). The analysis of the data revealed differences in the expression level of conserved miRNAs among the three species and among three the developmental stages of *O*. *felineus*. Analysis of miRNA genes revealed two gene clusters, one cluster-like region and one intronic miRNA in the genome. The presence and structure of the two gene clusters were validated using a PCR-based approach in the three flukes.

**Conclusions:**

This study represents a comprehensive description of miRNAs in three members of the family Opistorchiidae, significantly expands our knowledge of miRNAs in multicellular parasites and provides a basis for understanding the structural and functional evolution of miRNAs in these metazoan parasites. Results of this study also provides novel resources for deeper understanding the complex parasite biology, for further research on the pathogenesis and molecular events of disease induced by the liver flukes. The present data may also facilitate the development of novel approaches for the prevention and treatment of opisthorchiasis/clonorchiasis.

## Author Summary

Liver flukes of the family Opisthorchiidae cause diseases of the hepatobiliary system, known as opisthorchiasis/clonorchiasis. The chronic forms of these diseases greatly increase the risk of cancer developing in the biliary ducts. Much has been elucidated regarding the developmental biology of opisthorchiid flukes and the molecular pathological effects on the definitive host; however, the role of microRNAs (short non-coding RNAs) capable of influencing the pathogenic process and host-parasite interactions have not yet been comprehensively studied. The aim of the present work was to identify the miRNA genes of the liver flukes and provide a basis for further investigating the roles of these miRNAs in the complex opisthorchiidae life cycle and the pathogenesis of disease.

## Introduction

*Opisthorchis felineus*, *O*. *viverrini*, and *Clonorchis sinensis* (class Trematoda; order Plagiorchiida; family Opisthorchiidae) are parasitic flatworms with complex life cycles, which include three hosts, with human and piscivorous mammals as definitive hosts [1]. These three flukes cause diseases of the hepatobiliary system, referred to as opisthorchiasis/clonorchiasis. These diseases are characterized by chronicity and severe consequences, some of which are cancers of the biliary tract and liver [2–5]. *C*. *sinensis* is endemic in China, Taiwan, Vietnam, Korea, Japan, the Lao People's Democratic Republic and the Russian Far East; *O*. *viverrini* is found in Cambodia, the Lao People's Democratic Republic, Thailand, and Vietnam; and *O*. *felineus* is spread in the former Soviet Union (Ukraine, Belarus, Kazakhstan, the Baltic Republics and Russia, particularly Western Siberia) and some European countries [6,7].

Recently, many studies focusing on the developmental biology of the opisthorchiid flukes and the molecular mechanism of their pathological effects on host organisms were conducted using advanced genomic and transcriptomic techniques. For example, protein-coding transcriptomes have been well characterized for *O*. *felineus* [8], *O*. *viverrini* [9,10] and *C*. *sinensis* [9,11,12], allowing investigations of diverse issues of the host-parasite interaction at the molecular and cellular levels as well as indicating the diagnostic potential of particular proteins from the excretory secretory products (ESP) of the flukes. However, the microRNA-containing transcriptomes, which are known to dramatically influence many protein patterns, have not been comprehensively studied to date in opisthorchiid flukes.

It is well known that microRNAs (18–22 nucleotide, non-coding RNAs) are able to down-regulate target mRNA expression at the post-transcriptional level in multicellular animals and thus play important roles in many biological processes including development, differentiation, viral defense and apoptosis [13]. A miRNA becomes mature after processing of its stem-loop precursors by RNase III enzymes with short miRNA duplex generation. In addition, miRNA becomes functionally active upon detachment from its complement (miRNA*) in the duplex during integration into RNA-induced silencing complexes (RISC) [13,14]. Both the miRNA and the miRNA* are potentially functional in the RISC [15–18]; however, only one miRNA remains functional, and the other degrades [19–21]. The RISC-containing miRNA induces translational repression or the degradation of the target mRNA by binding to its 3’-UTR [14,22].

Increasing evidence shows that the action of miRNAs has great importance and broad roles in pathogen-host interactions and the regulation of immunity against infectious agents [23]. Recently, miRNAs have been detected circulating outside of cells in the serum within exosomes or in association with specific proteins [24]. These extracellular RNAs are stable in bodily fluids [24] and are involved in cell-to-cell communication [25,26]. Therefore, they have attracted attention as biomarkers of disease [26,27]. Moreover, parasite-derived miRNAs have recently been identified in the serum of hosts infected with *Schistosoma mansoni* [28] and in exosome-like vesicles in the ESP (*Dicrocoelium dendriticum*) [29]. MiRNA manipulation in parasites has been also proposed as a new strategy for controlling schistosomiasis and cystic echinococcosis [23]. Parasite miRNA studies have thus become promising for elucidating the molecular mechanisms of parasitic diseases and for the development of more specific diagnostic tools [30].

In the last decade, numerous miRNAs have been discovered in several flatworms species, such as *Schmidtea mediterranea* [31–33], *Dugesia japonica* [17,34], *Orientobilharzia turkestanicum* [35], *S*. *mansoni* [36,37], *S*. *japonicum* [38–40], *C*. *sinensis* [41], *Eurytrema pancreaticum* [42], *Echinococcus granulosus*, *E*. *multilocularis* [43], *Fasciola gigantica*, *F*. *hepatica* [44], *D*. *dendriticum* [29], *Hymenolepis microstoma* [45], *Taenia saginata* [46] and *Gyrodactylus salaris* [47]. Most of the miRNAs of *E*. *granulosus*, *E*. *multilocularis*, *S*. *japonicum*, *S*. *mansoni* and *S*. *mediterranea* have been described and are well annotated in miRBase (Release 21: June 2014). All proteins necessary for miRNA maturation and miRNA-induced silencing were identified in several flatworms species, for example, in *S*. *mansoni* [48]. The set of orthologous proteins were also found in opisthorchiid species [10,12,49]. So the description of miRNA transcriptomes of opisthorchiids is necessary for understanding gene expression and function in these parasites.

The aims of the present study were to identify the miRNAs of *O*. *felineus*, *O*. *viverrini* and *C*. *sinensis*, describe respective miRNA genes and provide a basis for further investigations of the roles of miRNAs in the regulation of gene expression in liver flukes.

## Materials and Methods

Adult worms of *C*. *sinensis*, *O*. *felineus*, and *O*. *viverrini*, as well as *O*. *felineus* metacercariae were taken for five RNA sample preparations. The first sample was prepared from adults of *C*. *sinensis* (14 flukes) that had been grown in rats (*Rattus norvegicus*) from metacercariae harvested from naturally infected Amur bitterling (*Rhodeus sericeus*) from the Bolshaya Ussurka river (Primorsky Krai, Russian Far East). The second sample was prepared from adults of *O*. *viverrini* (20 flukes) that were grown in golden hamsters (*Mesocricetus auratus*) from metacercariae extracted from naturally infected cyprinoid fish captured in Khon Kaen province (Thailand). The third and fourth samples were prepared from adults of *O*. *felineus* (20 flukes) that were grown in golden hamsters from metacercariae harvested from naturally infected ides (*Leuciscus idus*) from the Ob’ river (Novosibirsk city). The two *O*. *felineus* samples were Adult+Eggs—the manually dissected body portion with distal branches of the uterus filled with the eggs containing embryos (miracidia), and AdultNoEggs—the remaining body portion. The fifth sample (further as metacercariae) was prepared from 5000 *O*. *felineus* metacercariae from the same source.

The territories where sample collection (fishing) took place were neither conservation areas nor private or otherwise protected areas; hence, no fishing permits were required. The fish species collected are not considered endangered or rare, and fishing methods were in full compliance with the Federal Law N166-F3 of 20.12.2004 (ed. 18.07.2011) "Fishing and conservation of water bio-resources”. This study was conducted in strict accordance with the recommendations in the Guide for the Care and Use of Laboratory Animals of the National Institutes of Health. The protocol was approved by the animal ethics committee of the Institute of Cytology and Genetics (Permit Number: 7 of 19.11.2011). Euthanasia was performed by decapitation, and all efforts were made to minimize suffering.

### RNA preparation

For the detection of small RNAs of the three opisthorchiids, an enrichment technique consisting of the selective fractionation of RNA (18–200 nt) in polyethylene glycol solutions of various concentrations was used as described by Wang *et al*. [50]. The size distribution of the RNA molecules was analyzed by micro-electrophoresis with a BioAnalyzer (Agilent).

### SOLiD sequencing

The miRNA libraries were constructed using an Ambion® SOLiD Small RNA Expression Kit. For each sample, three libraries (technical replicates) were sequenced: two with Adaptor Mix A (yields the template for SOLiD sequencing from the 5' end of the sense strand) and one with Adaptor Mix B (yields the reverse complement sequence).

The cDNA libraries were produced using 200 ng of the small RNA fraction, following the protocol supplied with the kit, and amplified using barcoded primers and 17 PCR cycles for Mix A libraries and 15 PCR cycles for Mix B libraries. Amplified products were concentrated using the Fermentas® GeneJET PCR Purification Kit and gel purified using 6% acrylamide gels. Gel pieces containing PCR products of ~105–150 bp were excised, libraries were eluted by 5M ammonium acetate and cleaned by ethanol precipitation. Each library was diluted to a concentration of 0.5 pM for full-scale template bead preparation. Approximately 40 million beads for each sample were deposited on ¼ slide of the SOLiD 3.5 System and sequenced in 35-base runs. Sequencing was performed at the Siberian Branch of Russian Academy of Science (SB RAS) Genomics Core Facility. The library designations with corresponding GenBank database accession numbers are:

1. *C*. *sinensis*—A1 (SRX817942), rA1 (SRX817990), B1 (SRX817989)
2. *O*. *viverrini*—A2 (SRX817991), rA2 (SRX817993), B2 (SRX817992)
3. *O*. *felineus*
  - AdultNoEggs—A3 (SRX817994), rA3 (SRX817996), B3 (SRX817995)
  - Metacercaria—A4 (SRX817997), rA4 (SRX817999), B4 (SRX817998)
  - Adult+Eggs—A5 (SRX818000), rA5 (SRX818002), B5 (SRX818001)

### Computational analysis

**Fig 1:**
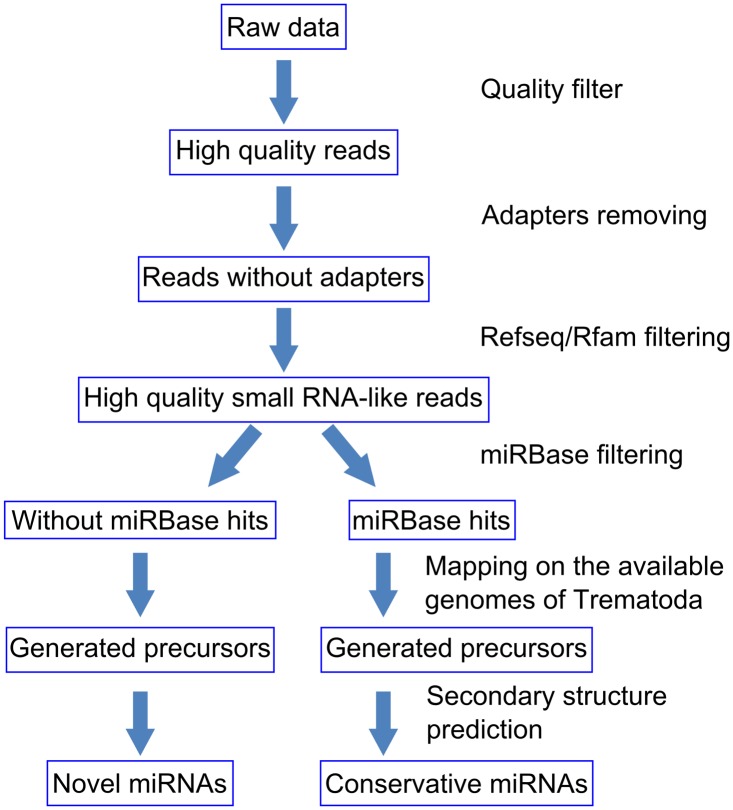
Computational pipeline for analyzing small RNA sequencing data.

The pipeline of the computational search for conserved and novel miRNAs in the opisthorchiid species is presented in Fig 1. First, quality filtering of the sequences was performed using the SOLiD preprocess filter [51] using the following parameters: *Min count for Polyclonal Analysis—1*, *Min QV for Polyclonal Analysis—25*, *Max count permitted errors—100*, *Max QV to consider an error—10*, *Removal of reads with negative QV score—y*, *and Truncation—off*. The adapter fragments were removed by cutadapt v. 0.9.5 [52] with a maximum error rate of 12.0% and a minimum read length of 18 bp.

To remove possible fragments of messenger and non-microRNA sequences, we mapped the reads to mRNA sequences in Refseq (rel. 106) [53], mRNA sequences of plathyhelmints and nematode taxa from the GenBank database (December, 2011) [54], and sequences from Rfam (rel. 10), [55] excluding miRNAs using BFAST [56]. The BFAST program was chosen, since it allows the mapping of short reads and uses the Smith-Waterman method, with gaps to support the detection of small indels at its final processing stage. This step improves the sensitivity of alignment, which, in our case, is important for mapping reads to genomes from different species. A significant advantage of this approach is that the alignment of sequences in the SOLiD 2-base color coding reduces the influence of sequencing errors. The following BFAST parameters were used: editing distance (the number of substitutions/insertions/deletions allowed in read alignment) ≤ 2, multiple mapping of reads was allowed, and other parameters were set as default. All reads mapped to these databases were removed from further analysis.

To identify conserved miRNAs, the remaining reads were mapped to animal pre-miRNA sequences in miRBase (Release 21: June 2014) [57] using BFAST with the following parameters: editing distance ≤ 4, multiple mapping of read was allowed, and other parameters were set as default. To identify genome-specific sequences of known miRNAs, we performed additional mapping of reads similar to miRBase sequences onto *C*. *sinensis* [58], *S*. *mansoni* (rel. 4) [59] and *S*. *japonicum* (rel. 2) [60] genomes with editing distances ≤ 2. To verify that these sequences can form pre-miRNA hairpins within their genomic context, the secondary structures of these candidate pre-miRNAs were reconstructed using the UNAFold program [61]. Two variants of the candidate pre-miRNA sequences were selected. The first variant spans from 50 bp upstream to 10 bp downstream of the miRNA region. The second variant spans from 10 bp upstream to 50 bp downstream of the miRNA region. We inferred miRNA sequences that met the following criteria: (1) ΔG ≤ -20 kcal / mol; (2) the fraction of paired nucleotides in the hairpin corresponding to the mature miRNA is > 70%; (3) no branching interactions for the hairpin forming nucleotides are allowed; (4) the sequence of miRNA is not in the terminal loop; and (5) the difference in the side lengths of internal loops and bulge size is not more than two nucleotides [62,63].

To identify novel miRNAs, the small RNA-like reads without similarity to sequences in miRBase were mapped to the genomic sequences of *C*. *sinensis*, *S*. *mansoni*, and *S*. *japonicum*. RepeatMasker (http://www.repeatmasker.org/) was used to mask repeats and regions with low complexity in the genomes. We used BFAST with an editing distance of ≤ 2, filtered out multiple mapped reads, and other parameters were as default. Genomic regions with lengths of ≤ 25 bp that were covered by at least three reads were considered as candidates for novel species-specific miRNAs. To verify the stem-loop pre-miRNA secondary structure of these sequences, we applied UNAFold analysis for their two extended sequence variants. The sequences meeting the above mentioned secondary structure criteria were considered as novel miRNA candidates.

To estimate reproducibility of technical replicates, the Spearman's rank correlation coefficients of normalized (RPKM) expression level of several conserved miRNAs (that are common for three flukes) were established using Past3 [64] (S1 Table). Conserved miRNAs were used in reproducibility analysis because new miRNAs have low non-normalized expression levels (around three reads were mapped to the genome for each new miRNA; therefore, the novel miRNAs were not detected in all technical replicates).

Additional similarity searches were performed using the BLAST [65]. To detect violations of one of the criteria of the conservative cluster definition (cluster of miRNAs should be a group of miRNA precursors expressed as a polycistronic unit [66]) we applied the protein coding gene-finding procedure using the Fgenesh program [67].

The alignments of some miRNAs (two miR-71/ miR-2 clusters, miR-1, miR-133, and miR-190) with sequences of these miRNAs orthologs (obtained from *S*. *mediterranea*, *G*. *salaris*, *S*. *mansoni*, *S*. *japonicum*, *E*. *granulosus*, *E*. *multilocularis*, *H*. *microstoma* and *T*. *solium* genomes) were performed using the program CLUSTALW [68]; miRNA sequences of *T*. *solium*, namely miR-1, miR-2b, miR-2c, miR-71, miR-133, miR-190, were obtained by homology search of these miRNAs in *T*. *solium* genome (http://www.genedb.org/Homepage/Tsolium) using the BLAST [65]. All time-consuming computations were performed using a high-throughput computing system at the Joint Access Center for Bioinformatics and a computational cluster at the Novosibirsk State University.

### Genomic region PCR amplification and sequencing

The following primers were used for the amplification of genomic regions hosting the miRNA genes of the three opisthorchiid species: clust1-for1 (5'-CACAGCCAGTATTGATGAAC-3'), clust1-for2 (5'-ACAGCCCTGCTTGGGACAC-3'), clust1-rev (5'-CCAAAGCTTGGACTGTGAT-3'), clust2-for (5'-AAAGACTTGAGTAGTGAGACGCT-3'), clust2-rev (5'-TCGTCACCTAAGCAGGACT-3'), Cl1-F (5'-CGCAAGTGATCAATGTTTTCCTC-3') and Cl1-R (5'-GCGCACCAACGGCCTAA-3'). The amplification was conducted using a DNA thermal cycler (Mastercycler gradient Eppendorf) as follows: initial denaturation at 95°C for 2 min, followed by 35 amplification cycles (95°C for 25 s, 56°C for reactions with clust1-rev, clust1-for1, clust1-for2, Cl1-F and Cl2-R and 53°C for reactions with clust2-for and clust2-rev for 30 s, 72°C for 30 s) and a final extension cycle (72°C for 5 min). PCR products were analyzed by agarose gel (2%) electrophoresis. Purification of PCR products was performed by the method of Exo-TsAP. To 20 μl of PCR product were added 1 μl of Exonuclease I and 1 μl of Thermosensitive Alkaline Phosphatase, followed by an incubation for 15 min at 37°C and then 15 min at 80°C. Sequencing reactions were performed using the BigDye ® Terminator v3.1 Cycle Sequencing Kit according to the manufacturer's instructions and analyzed at the SB RAS Genomics Core Facility.

## Results

### Computational identification of miRNAs

A three-step mapping and filtering procedure was applied to the reads (Fig 1) generated from the 15 libraries to obtain the pool of small RNA-like sequences for the three opisthorchiid species. The results of filtering are given in S2 Table. For *O*. *felineus*, the sequencing of nine libraries generated 446 million reads that were distributed as follows: 131 millions for three Adult+Eggs libraries, 152 millions for three AdultNoEggs libraries, and 162 millions for three Metacercaria libraries. For *C*.*sinensis*, three libraries were sequenced and 126 million reads were obtained. For *O*. *viverrini*, three libraries were sequenced with 150 million reads obtained. After filtering low quality tags, including 5′ and 3′ adaptors and adaptor-adaptor ligation products, a total of 279 million reads with high quality were retained for *O*. *felineus* (Adult+Eggs (84 million reads), AdultNoEggs (108 million reads), Metacercaria (87 million reads)), 75 million reads for *C*. *sinensis*, and 83 million for *O*. *viverrini*. Among the clean reads, an average of 13.6% were found to be rRNA, tRNA, snRNA, and snoRNA, when searched against the Refseq/Rfam databases. The percentage of the remaining reads mapping to miRBase sequences averaged 2.85%. Spearman's rank correlation coefficient analysis showed high reproducibility between A and rA libraries (~ 0.9) and somewhat less reproducibility between B and either A or rA libraries (~ 0.8), which might be explained by the fact that rA libraries were exact technical replicates of A libraries whereas B libraries were created using another adaptor.

**Fig 2:**
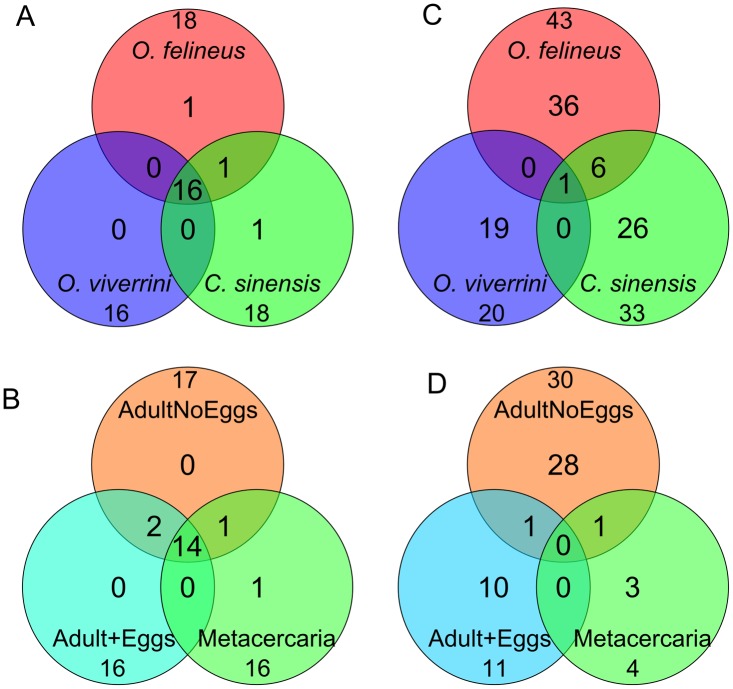
Venn diagrams of the miRNAs sets. (A) conserved miRNAs in three opisthorchiid species, (B) O. *felineus* conserved miRNAs at different developmental stages, (C) novel miRNAs in the three opisthorchiid species, (D) O. *felineus* novel miRNAs at different developmental stages.

The miRNA was regarded as conserved if it had an ortholog in another animal species. The ortholog search for the miRNAs of the three opisthorchiids yielded 19 conserved miRNAs belonging to 13 families (bantam, let-7, miR-1, miR-2, mir-7, miR-10, miR-36, miR-46, miR-71, miR-124, miR-125, miR-133, and miR-190) (Fig 2A, Table 1, S3 Table). Most families included one miRNA variant, but the miR-71 family consisted of two variants and the miR-2 family comprised five variants. Interestingly, the expression of miRNA* from the duplex carrier strands for two miRNAs (let-7 and miR-10) was also found (S3 Table).

**Table 1:** List of conserved miRNAs identified in three Opisthorchiidae species.

Species	miRNAs
*O*. *felineus*, *C*. *sinensis* and *O*. *viverrini*	bantam, let-7, miR-1, miR-2(a,b,c,d,e), miR-7, miR-36(a), miR-71(a,b), miR-124, miR-125, miR-133, miR-190
*O*. *felineus* and *C*. *sinensis*	miR-281 (miR-46 family)
*O*. *felineus*	miR-10
*C*. *sinensis*	miR-36b

Sixteen conserved miRNAs were identified as common in all three opisthorchiids. Additionally, miR-281 (miR-46 family) was found in two species—*O*. *felineus* and *C*. *sinensis*. There were also conserved miRNAs either in *O*. *felineus* only (miR-10) or in *C*. *sinensis* only (miR-36b) (Fig 2A; Table 1).

Eighteen conserved miRNAs were identified for *O*. *felineus* when combining the Adult+Eggs, AdultNoEggs, and Metacercaria samples. Individual analyses of the *O*. *felineus* samples (Adult+Eggs, AdultNoEggs, Metacercaria) revealed differences in miRNA composition between the samples. Fourteen of the eighteen *O*. *felineus* miRNAs were identified in all three samples. Two miRNAs (bantam and miR-281) were identified in AdultNoEggs and Adult+Eggs samples only, but not in the Metacercaria sample. miR-7 was detected in AdultNoEggs and Metacercaria samples but not in the Adult+Eggs sample, and miR-10 was found in the Metacercaria samples only (Fig 2B, Table 2). The mapping results demonstrated that most of the conserved miRNA sequences identified in the present study are common among opisthorchiid and schistosome species, which was expected.

**Table 2:** List of conserved miRNAs identified in different samples of *O*. *felineus*.

Developmental stage of *O*. *felineus*	miRNAs
AdultNoEggs & Adult+Eggs & Metacercaria	let-7, miR-1, miR-2(a,b,c,d,e), miR-36, miR-71(a,b), miR-124, miR-125, miR-133, miR-190
AdultNoEggs & Adult+Eggs	bantam, miR-281(miR-46 family)
AdultNoEggs & Metacercaria	miR-7
Metacercaria	miR-10

Candidate sequences for novel miRNAs (S4 Table) were selected from reads without matches to miRBase sequences after mapping them to the *C*. *sinensis* genome and processing the genomic fragments encompassing the resultant hits through the secondary structure filter (see Materials and Methods). We identified 43 such miRNAs for *O*. *felineus*, 20 for *O*. *viverrini* and 33 for *C*. *sinensis*. The occurrence of novel common and species-specific miRNAs in the samples from the three Opisthorchiidae species is presented in Fig 2C and S4 Table.

Interestingly, most of these novel miRNAs were species-specific. Only one miRNA (new_miR-001) had orthologs in all three species. The greatest number of novel specific miRNA candidates was identified for *O*. *felineus* (83%); however, the fraction of unique species-specific miRNAs was highest for *O*. *viverrini* (95%).

Forty-three novel miRNAs were obtained for *O*. *felineus* when combining the Adult+Eggs, AdultNoEggs and Metacercaria samples. The distribution of stage-specific and stage-nonspecific novel miRNA candidates in *O*. *felineus* demonstrated that no common miRNAs were identified in all three sample types, and only two of the 43 novel miRNAs were identified in more than one stage/body part (Fig 2D).

### Genomic organization of opisthorchiid miRNA genes

**Fig 3:**
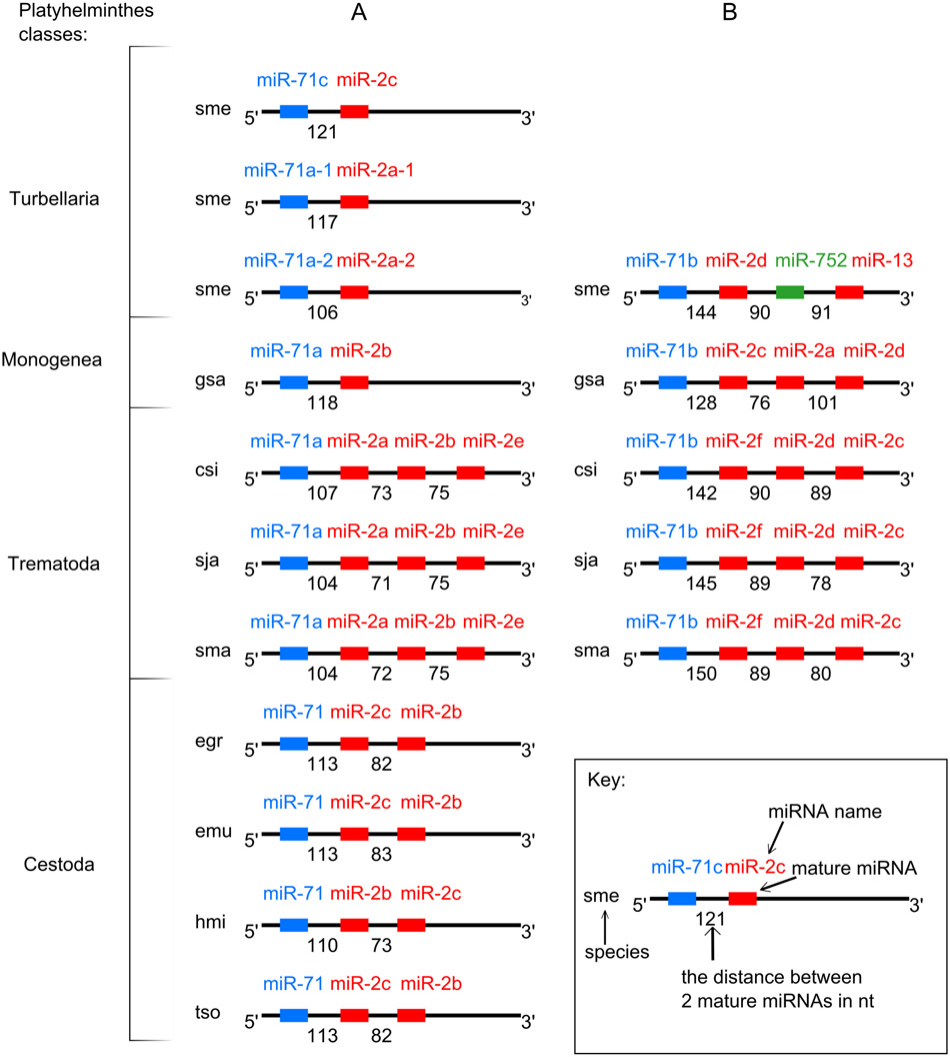
Scheme of miRNA gene clusters in Platyhelminthes. (A) miR-71a/2 cluster group, (B) miR-71b/2 cluster group. Species designations: csi—C. sinensis, sma—S. mansoni, sja—S. japonicum, sme—S. mediterranea, hmi—H. microstoma, egr—E. granulosus, emu—E. multilocularis, gsa- G. salaris.

Mapping the conserved miRNAs onto the *C*. *sinensis* genome provided evidence supporting the presence of two miRNA clusters: miR-71a/miR-2a/miR-2b/miR-2e (miR-71a/2) and miR-71b/miR-2d/miR-2c (miR-71b/2). Homologous clusters have been previously described for seven flatworms (*E*. *granulosus*, *E*. *multilocularis*, *G*. *salaris*, *H*. *microstoma*, *S*. *mediterranea*, *S*. *japonicum* and *S*. *mansoni*) [45,47], and in the current study, were also found in the *T*. *solium* genomic sequences (Fig 3, S1 Appendix). We compared the structures of these miRNA clusters from the *C*. *sinensis* genome with homologous sequences from the flatworm genomes mentioned (Fig 3).

All mature miRNA sequences of miR-71 family were located in the 5’ arm of their precursors, while all mature miRNAs of the miR-2 family were located in the 3’ arm [69]. In the miR-71a/2 cluster group, the distance between the mature miR-71 and the nearest miR-2 varied from 104 to 121 bp; in the miR-71b/2 cluster group, this distance ranged from 121 to 150 bp. The minimal distance between the two mature sequences of miR-2 family was found in the miR-71a/2 group (71 bp between miR-2a and miR-2b in *S*. *japonicum*); the maximal distance was found in the miR-71b/2 group (101 bp between miR-2a and miR-2d in *G*. *salaris*) [45,47].

The miR-71a/2 cluster mapped to *C*. *sinensis* contig 2339 and spanned 441 nucleotides (from 103849 to 104290), with the miRNA order the same as in both *Schistosoma* genomes. A comparative analysis of the miR-71a/2 cluster genomic organization among the flatworms revealed three distinct types (Fig 3A). The first type, comprising the precursors for miR-71a and the three miR-2 isoforms, was exemplified by clusters from the genomes of *C*. *sinensis*, *S*. *japonicum*, and *S*. *mansoni*. The second type, consisting of the precursors for miR-71 and the two miR-2 isoforms, was represented in the genomes of the cestodes *H*. *microstoma*, *E*. *granulosus* and *E*. *multilocularis*. The third type, with the precursors for miR-71 and only one miR-2 isoform, was observed in the monogenean *G*. *salaris* and the planarian S. *meditteranea* (in three genomic copies) [45,47].

The miR-71b/2 cluster mapped to *C*. *sinensis* contig 2957 and spans 416 nucleotides (from 323569 to 323984). Detailed analysis of the cluster sequences in three trematode, one monogenean and one turbellarian genome resulted in the discovery of the precursor for miR-2f in *C*. *sinensis* (Fig 3B, S5 Table). This miRNA was previously described for two schistosomes [70,71]. The miRNA order in these orthologous clusters was also well conserved in the *C*. *sinensis*, *S*. *japonicum* and *S*. *mansoni* genomes (Fig 3B). It should be noted that sme-miR-752, although not formally assigned to the mir-2 family, is recognized as having evolved from miR-2 [47].

### Experimental verification of the miRNA clusters miR-71a/miR-2a/miR-2b/miR-2e and miR-71b/miR-2f/miR-2d/miR-2c

**Fig 4:**
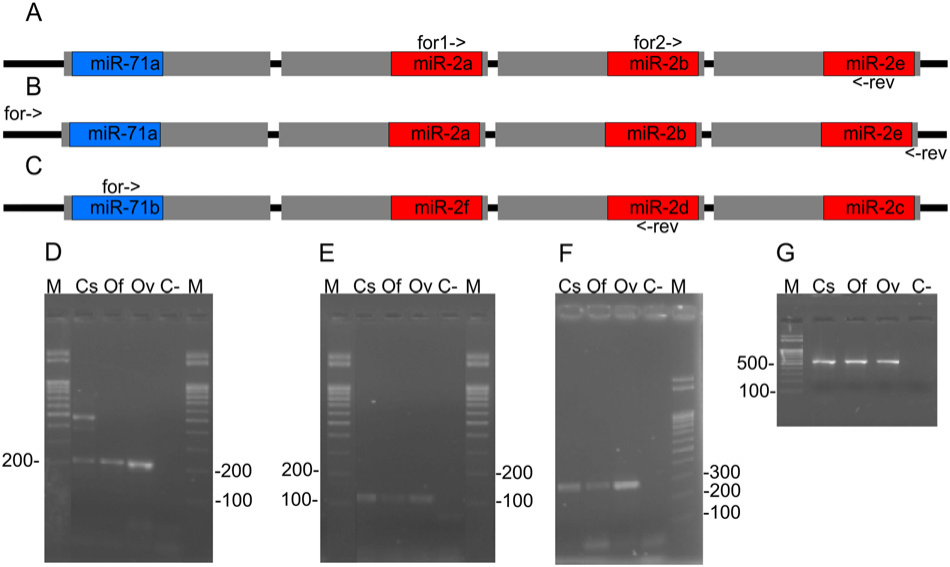
Upper: Scheme of primer target site positions in clusters: (A and B) miR-71a/2, (C) miR-71b/2. Grey rectangles mean miRNA precursors, blue, purple and read rectangles—mature miRNAs; for->, for1->, for2-> and <-rev indicate primers and their directions. Lower: electrophoretogram of PCR products generated out of the cluster genomic regions. Designations: C-—negative control, M—lengths marker. DNA templates: Cs—*C*. *sinensis*, Of—*O*. *felineus*, Ov—*O*. *viverrini*. Primers used: (D) clust1-for1 and clust1-rev; (E) clust1-for2 and clust1-rev; (F) clust2-for and clust2-rev; G) Cl1-F and Cl2-r. The original electrophoretogram photographs are presented in S1 Fig.

Because the mature miRNA sequences of the two clusters were identical among the three opisthorchiid species, we designed two primer sets to experimentally prove the presence of the clusters and partially structure the clusters using PCR amplification of corresponding regions in the three genomes. To amplify a fragment of cluster miR-71a/2, the primer set clust1-for1, clust1-for2, clust1-rev was used (Fig 4A, S2 Appendix). For the miR-71b/2 cluster, the primer set clust2-for, clust2-rev was employed (Fig 4C, S2 Appendix). The electropherogram presented in Fig 4D, 4E and 4F) show the PCR products generated using these primer sets with DNA templates prepared from *C*. *sinensis*, *O*. *felineus* and *O*. *viverrini*.

The sequence alignments of the corresponding genomic regions of the three opisthorchid species revealed specific variable positions: 8 per 387 nucleotides in cluster miR-71a/2 and 11 per 299 nucleotides in cluster miR-71b/2 (S3 Appendix). These variable positions were located mainly in the regions corresponding to the ends of the pre-miRNA, the terminal loops of the pre-miRNA and the spacers between miRNA precursors.

It is worth noting that miR-2f from cluster miR-71b/2 of *C*. *sinensis*, *S*. *japonicum* and *S*. *mansoni* (Fig 3) was also discovered in the respective clusters of *O*. *felineus* and *O*. *viverrini*. Furthermore, the alignment of this genomic region demonstrated high conservation among the three opisthorchiid species: only four variable positions (which were located closer to the precursor of miR-2d) per 154 nucleotides were found.

To experimentally prove the overall structure of the miR-71a/2 clusters in the three species, we designed primers Cl1-F and Cl1-r, which are capable of amplifying the genomic regions encompassing the clusters, using the only available sequences for *C*. *sinensis* (Fig 4B, S1 Appendix). The results are presented in the electrophoretogram (Fig 4G).

The sequencing of the three species-specific amplicons (S4 Appendix) allowed us to determine the four pre-miRNA sequences for each of the three flukes. The secondary structures of these pre-miRNAs were estimated by UNAFold (S2 Fig). The results of UNAFold demonstrated that the nucleotide substitutions discriminating the pre-miRNA sequences of each of the opisthorchiid species exerted minor or no effects on the pre-miRNA secondary structures.

### Cluster-like regions miR-1/miR-133

Upon analysis by Jin *et al*. [45], the genomic regions with matches for miR-1 and miR-133 were designated as orthologous miRNA gene clusters in three flatworms, namely the cestodes *E*. *granulosus*, *E*. *multilocularis* and *H*. *microstoma*.

We extended this list of flatworm species by demonstrating that *C*. *sinensis* and *S*. *mansoni* also have similar genomic regions. It should be mentioned that miR-133 were not annotated for *S*. *mansoni* in previous reports [36,37,71]. However, we found sequences highly similar to this miRNA in read archives (ERR278825, ERR278826, ERR278827, ERR278828) using a BLAST search. The UNAFold secondary structure prediction for the precursors of the conserved miRNAs showed no canonical structure for the putative *S*. *mansoni* pre-miR-133, which could possibly explain the delay in sma-miR-133 annotation (S6 Table).

**Fig 5:**
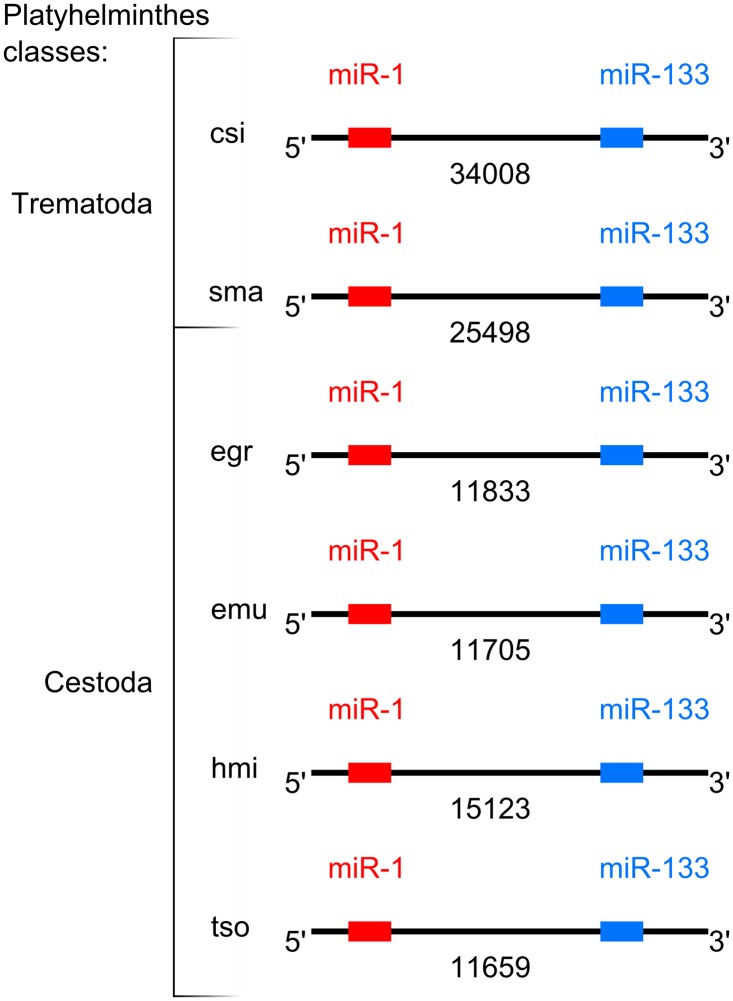
Genomic organization scheme of cluster-like regions miR-1/miR-133 in five flatworms. Designations are the same as in Fig 3.

Our alignment analysis did not show complete conservation over these regions of the five genomes. Remarkably, large spacers were detected between the sites matching the miRNAs, ranging from 11705 bp in *E*. *multilocularis* to 34008 bp in *C*. *sinensis* (Fig 5, S5 Appendix). Hence, we referred to the regions as “cluster-like regions miR-1/miR-133”.

To elucidate the content of the spacers in genomes of five parasitic flatworms, we employed the gene prediction program Fgenesh [67] using *S*. *mansoni*-specific gene-finding parameters and found few unannotated ORFs without significant similarity among the species (S6 Appendix). We then explored the genomic context beyond the cluster-like regions miR-1/miR-133 in the five flatworm species using information from the *C*. *sinensis* database (http://fluke.sysu.edu.cn/CsinGeno/home.php), NCBI (http://www.ncbi.nlm.nih.gov) (for *S*. *mansoni*) and Genedb (http://www.genedb.org/Homepage) (for *E*. *granulosus*, *E*. *multilocularis* and *H*. *microstoma*). We found that miR-133 is located near a gene encoding one of several Mind bomb proteins in all five genomes. We also found that miR-1 mapped near a gene encoding another Mind bomb protein in the genomes of *S*. *mansoni*, *E*. *granulosus* and *E*. *multilocularis* (S7 Table).

Although these miRNA sites were conservatively linked (forming a putative synteny group), the inter-microRNA distances exceeded 10 kb and contained putative genes. Altogether, the features suggested that the expression of these two miRNAs was unlikely as a single transcriptional unit in either genome. Therefore, we concluded that the case under consideration did not adhere to the conservative definition for a miRNA gene cluster [66].

### miR-190 is an intronic miRNA

The mapping of miR-190, which was also identified in the three opisthorchiid species, to the available flatworm genomic sequences showed that this miRNA is located in an intron of the gene encoding the talin protein. Therefore, we could classify this miR-190 as intronic [72]. It is noteworthy that, despite some variability in the nucleotide content of the talin exons surrounding the intronic miRNA (S7 Appendix), the overall protein structure was conserved enough (S8 Appendix) to ensure a reliable comparative analysis of the gene structure (Table 3).

**Table 3:** Structures of Trematoda and Cestoda talin genes with intronic miR-190.

Species	Gene id	Gene length in bp	Exons counts	Number of intron containing miRNA	Intron length in bp
Trematoda					
*C*. *sinensis*	csin001953	70200	37	32^nd^	2574
*S*. *japonicum*	Sjp_0006570	139915	43	40^th^	685
*S*. *mansoni*	Smp_037860	12434	10	7^th^	1237
Cestoda					
*E*. *granulosus*	EgrG_000736000	26856	48	44^th^	225
*E*. *multilocularis*	EmuJ_000736000	26634	43	39^th^	224
*H*. *microstoma*	HmN_000220000	43376	41	37^th^	356
*T*. *solium*	TsM_000902500	27324	44	40^th^	225

The intronic miRNA showed the motives corresponding to both mature miR-190 and miR-190*(S9 Appendix). The alignment depicted the sites with high conservation in either the flatworm class and those with evident inter-class variations, which, nevertheless, likely did not hamper the intronic miRNA’s ability to form the necessary secondary structure and effectively undergo maturation.

## Discussion

Using deep sequencing with SOLiD technology, we have identified 88 novel and 19 conserved miRNAs in three liver flukes of the family Opisthorchiidae—*C*. *sinensis*, *O*. *felineus*, and *O*. *viverrini*. The discovery of the novel opisthorchid-specific miRNAs is interesting, since they could be responsible for some opisthorchid-specific features of their parasitic life style including some pathogenicity features in definitive hosts. Interestingly, the number of the novel species-specific candidate miRNAs identified in the opisthorchiid flukes was larger than that of conserved miRNAs. This may relate to a low coverage of individual novel miRNAs (three reads were mapped to the genome for each new miRNA). Nevertheless, it is worth noting that similar species-specific/conserved miRNA families ratios (miRBase Release 21: June 2014) are also observed for other trematodes—*S*. *japonicum* (28/22) and *S*. *mansoni* (82/22). The same is seen also for free living planarian *S*. *mediterranea* (45/44). The addition of our data on the miRNAs of opisthorchiid flukes likely raises the question as to whether an excess of novel (species-specific) miRNAs compared with conserved (family-, class-, and phylum-specific) miRNAs could relate to a difference in life style (free vs. parasitic), as proposed previously [36]. It seems that even a less significant difference in parasitic style between schistosomes or opisthorchiids is associated with generation of numerous family-, genus-, and species-specific miRNAs. More additional data for flatworms’ miRNAs are needed to elucidate the evolutionary and biological significance of species-specific miRNAs of parasitic flatworms.

The identification of 19 conserved miRNAs in three liver flukes of the family Opisthorchiidae has strengthened the results of previous attempts to explore miRNAs of liver flukes. It should be noted that, in a previous study of *C*. *sinensis*, numerous miRNA-like sequences were found among reads generated with high-throughput sequencing using Solexa/Illumina technology [41]. However, the authors had no opportunity to carry out mapping the miRNA-like sequences on to the *C*. *sinensis* genome to achieve a confident assignment of their miRNA-like sequence sets to miRNA families annotated in miRBase. Therefore, we now provide the results of miRNA-like sequence mapping on to the *C*. *sinensis* genome, thus improving the reliability of miRNA identification for members of the Opisthorchiidae. Furthermore, we provide the results of the miRNA family classification. The occurrence of the 19 conserved miRNAs in organisms of various taxa (including 10 miRNAs out of 34 ones arisen after “bilaterian expansion” [72]) is presented (S8 Table).

We should mention two curious *C*. *sinensis* miRNAs: the reads corresponding to csi-miR-36b were found in our study but were not found by Xu *et al*. [41], and the reads corresponding to miR-10 as indicated by Xu *et al*. [41] were readily mapped in the *C*. *sinensis* genome, but were detected in our study for *O*. *felineus* only. Perhaps these cases need further investigation.

The mapping of 19 conserved miRNAs on to the three genomes available for Trematoda (*C*. *sinensis*, *S*. *mansoni* and *S*. *japonicum*) are presented in Table 4. Interestingly, there was some shortfall in hits for few miRNAs after the mapping of sequencing data. This could be due to the incompleteness of either genome assembly (miR-125 was not found in *C*. *sinensis* genome) or indeed by the species specificity of miRNA genes (we did not find the opisthorchid miR-1 in *S*. *japonicum* genome, we also did not locate opisthorchid miR-36b in either schistosome genome).

**Table 4:** Results of conservative miRNAs mapping onto Trematoda genomes.

miRNA	Genomes		
	*C*. *sinensis*	*S*. *mansoni*	*S*. *japonicum*
bantam	+	+	+
let-7	+	+	+
miR-1	+	+	−
miR-2a	+	+	+
miR-2b	+	+	+
miR-2c	+	+	+
miR-2d	+	+	+
miR-2e	+	+	+
miR-7	+	+	+
miR-10	+	+	+
miR-36a	+	+	+
miR-36b	+	−	−
miR-281	+	+	+
miR-71a	+	+	+
miR-71b	+	+	+
miR-124	+	+	+
miR-125	−	+	+
miR-133	+	+	+
miR-190	+	+	+

Mapped miRNA is designated by plus; unmapped—by minus.

Our analysis of the genomic organization of the opisthorchid miRNA genes confirmed the presence of gene clusters and intronic miRNAs. It is known that the miR-71/miR-2 cluster, which we experimentally proved to be in two copies in opisthorchiids (like in other parasitic trematodes studied) is present as one copy in parasitic cestodes, and five copies in the free-living planarian *S*. *mediterranea* [45]. This variation in the number of miR-71/miR-2 clusters in the genomes of representative flatworms of different classes could not be explained by the biology of the organism or by the reduction of targets for these miRNAs. The parasitic nematode *Ascaris suum* and *Brugia malayi* display one miR-71/miR-2 cluster, while the freeliving *Caenorhabditis* species have either one or no such cluster (miRBase Release 21: June 2014). Therefore, it seems that the miR-71/miR-2 cluster evolution proceeded differently in the Nematoda compared with the Platyhelminthes, and the details of the evolution remains to clarify in further studies.

Both clusters miR-71a/miR-2a/miR-2b/miR-2e and miR-71b/miR-2f/miR-2d/miR-2c were conserved, suggesting their functional importance in all three opisthorchiid species (Fig 3). To date, some miRNAs belonging to miR-71 and miR-2 families are known to have female-biased expression in *S*. *mansoni* [71] and to play an important role in regenerative processes in planarian [73]. Also the miR-2 family miRNAs are probably involved in neural development and maintenance in *Drosophila melanogaster* and *C*. *elegans* [69]. Their detection in exosome-like vesicles in the ESP of the liver fluke *D*. *dendriticum* leads to a speculation about the possible implications of trematode miRNAs in the modulation of parasite-host interactions by a new means of regulating host gene expression [29].

The fact that expressed sequences (reads) corresponding to miR-2f have not been detected in opisthorchiids requires further studies to explain why the expression pattern of this putative miRNA is so strikingly different from that of its neighbors.

In previous papers, the combination of the miR-1/miR-133 miRNA genes was described also as a miRNA cluster for many animal species (see data in miRBase) [74] including flatworms [45]. However, this combination in *Drosophila* genomes has been shown to escape the conserved cluster definition [66]. Hence, due to the distance between the sites corresponding to the miRNAs in flatworms, as well as the capability to predict protein-coding genes in between these sites, we suggest referring to these regions as “cluster-like regions miR-1/miR-133”, which form a putative synteny group.

The next miRNA cluster that should be discussed is let-7/miR-100/miR-125. Its main characters are conserved in almost all Deuterostomia taxa. However, in Protostomia, many variations of its structure have been discovered, while in some animals (Annelida, *Trichinella*, Arthropoda), its general structure is conserved. Important is that the cluster was shown to be disintegrated in flatworms with a complete loss of miR-100 [75]. We can support this conclusion for opisthorchiids also. First, mir-100-like sequences were not detected in the three opisthorchiid species. Second, the combination of let-7/ miR-125 genes is unlikely to exist as a synteny group, as the two miRNA genes map to different chromosomes in *S*. *mansoni* (S9 Table).

The present analysis corroborates the classification of the miR-190 gene as intronic within the talin gene. The intronic nature of the miR-190 gene has been described for many animals [36,45,76]. High conservation of the structural (and maybe functional) association between miR-190 and the talin protein in platyhelminths appears very interesting and is worthy of further elucidation.

Just prior to submission of this manuscript, an article on the *O*. *viverrini* genome was published [49]. In the article, the authors predicted *in silico* 178 conserved miRNA genes. These data will give us the opportunity for a more detailed analysis of *O*. *viverrini* miRNA genes, in particular for a comparison of our data based on miRNA real expression with the results of *in silico* prediction based on genomic sequence analysis.

In conclusion, the present study presents the results of large-scale identification and characterization of miRNAs sets encoded in the genomes of *O*. *felineus*, *O*. *viverrini* and *C*. *sinensis*. This first comprehensive comparative analysis of the miRNA genes of these species allowed us to reveal the conserved and species-specific miRNAs in these sets. For several conserved opisthorchiid miRNAs, the genomic organization was analyzed by comparison with orthologous genes in other platyhelminths. The structures of two miRNA gene clusters were experimentally validated for the three opisthorchiid species. The differences in expression level found for some conserved miRNA among the three species and among the three stages of *O*. *felineus* stimulate studies to more precisely profile the expression of miRNAs. Finally, the present data provide a sound basis for further studies of the molecular mechanisms of host interactions of opisthorchiids and for development of novel methods to control these neglected parasites.

## Supporting Information

S1 Appendix Alignment of miRNA clusters. csi—*C*. *sinensis*, egr—*E*. *granulosus*, emu—E. multilocularis, gsa—*G*. *salaris*, hmi—*H*. *microstoma*, sja—*S*. *japonicum*, sma—*S*. *mansoni*, sme—*S*. *mediterranea*, tso—*T*. *solium*, sec. struc.—secondary structure. Mature miRNA sequences are in bold type and underlined. Clusters of S. mediterranea were obtained from contigs:
297 (gi|124128508|gb|AAWT01093310.1|) (- strand)1403 (gi|124163860|gb|AA WT01057958.1|) (+ strand) (locus 1)5146 (gi|124196975|gb|AA WT01024843.1|) (+ strand) (locus 2)2151 (gi|124194162|gb|AA WT01027656.1|) (+ strand)
Cluster of *C*. *sinensis* was obtained from contig 2339 (gi|353340623|dbj|BADR02002339.1|) (- strand). Cluster of *S*. *mansoni* was obtained from chromosome W (gi|360043576|emb|HE601631.1|) (+ strand). Cluster of *S*. *japonicum* was obtained from contig S000054 (gi|227129020|emb|FN331028.1|) (- strand). Cluster of *H*. *microstoma* was obtained from contig 370001 (gi|528309775|emb|CBLW010002354.1|) (+ strand). Cluster of *E*. *granulosus* was obtained from contig 0003137 (gi|528307253|emb|CBLN010000400.1|) (- strand). Cluster of *E*. *multiloculari*s was obtained from contig 007728 (gi|528775369|emb|CBLO010002090.1|) (- strand). Cluster of *T*. *solium* was obtained from contig 01703 (pathogen_TSM_contig_01703) (+ strand). Cluster of *G*. *salaris* was obtained from scaffold 7180006951238_l_32618_C_75.085_gc_34.438 (+ strand)
(PDF)

S2 Appendix Position of primers target regions in clusters. csi—*C*. *sinensis*. Secondary structure of miRNA precursors is indicated by a parentheses and dots. Mature miRNA sequences including miR-2f are in bold type and underlined.
(PDF)

S3 Appendix Alignment of miR-71a/2a/2b/2e miRNA cluster fragment containing miR-2a, miR-2b and miR-2e. sec. struc.—secondary structure. Mature miRNA sequences are in bold type and underlined.
(PDF)

S4 Appendix Alignment of miR-71a/2a/2b/2e miRNA cluster. sec. struc.—secondary structure. Mature miRNA sequences are in bold type and underlined.
(PDF)

S5 Appendix Alignment of miRNA clusters like regions. csi—*C*. *sinensis*, sma—*S*. *mansoni*, hmi—*H*. *microstoma*, egr—*E*. *granulosus*, emu—*E*. *multilocularis*, tso—*T*. *solium*, sec. struc.—secondary structure. Mature miRNA sequences are in bold type and underlined. Mature miRNA sequences are in bold type and underlined. Cluster of *C*. *sinensis* was obtained from scaffold 198 (gi|353342189|dbj|BADR02000773.1|) (- strand). Cluster of *S*. *mansoni* was obtained from chromosome W (gi|360043576|emb|HE601631.1|) (- strand). Cluster of *H*. *microstoma* was obtained from contig 880003 (gi|528308588|emb|CBLW010003541.1|) (- strand). Cluster of *E*. *granulosus* was obtained from contig 00137 (gi|528306514|emb|CBLN010001139.1|) (+ strand). Cluster of *E*. *multilocularis* was obtained from contig 007760 (gi|528775363|emb|CBLO010002096.1|) (+ strand). Cluster of *T*. *solium* was obtained from contig 00015 (pathogen_TSM_contig_00015) (+ strand).
(PDF)

S6 Appendix Gene prediction in region between miR-1 and miR-133. (PDF)

S7 Appendix Exons surrounding intronic miRNA, and part of protein encoded by them. (PDF)

S8 Appendix Flatworms talin alignment. (PDF)

S9 Appendix Alignment of miR-190 precursors in the context of corresponding introns. (PDF)

S1 Fig Original photo of electrophoretograms. (PDF)

S2 Fig Secondary structure of miR-71a, miR-2a, miR-2b, miR-2e precursors from *C*. *sinensis*, *O*. *felineus*, *O*. *viverrini*. Mature miRNAs are red. Diferensis in sequences of miRNA precursors in red circles.
(PDF)

S1 Table Analysis of technical replicates reproducibility by Spearman's rank correlation coefficient of normalized expression level for several conserved miRNA. (XLSX)

S2 Table Results of raw data analysis. (XLS)

S3 Table List of Opisthorchiidae conserved miRNAs. (XLSX)

S4 Table List of Opisthorchiidae novel miRNAs. (XLSX)

S5 Table Trematodes miR-2f. (XLSX)

S6 Table miR-1, miR-133 and putative miR-1, miR-133. (XLSX)

S7 Table Genes around miR-1 and miR-133 in the flatworms genomes. (XLSX)

S8 Table Distribution of found conserved miRNA familis in bilateria. (XLSX)

S9 Table Location of lin-7 and miR-125 in flatworms genomes according miRBase (Release 21: June 2014 http://mirbase.org/). (XLSX)
