# Supplementary material for: Identification of microRNA Genes in Three Opisthorchiids
Source: PLoS Negl Trop Dis. 2015 Apr 21;9(4):e0003680. doi: 10.1371/journal.pntd.0003680 (PMC4405270; doi:10.1371/journal.pntd.0003680)
Supplement: S1 Appendix — csi—C. sinensis, egr—E. granulosus, emu—E. multilocularis, gsa—G. salaris, hmi—H. microstoma, sja—S. japonicum, sma—S. mansoni, sme—S. mediterranea, tso—T. solium, sec. struc.—secondary structure. Mature miRNA sequences are in bold type and underlined. Clusters of S. mediterranea were obtained from contigs: 297 (gi|124128508|gb|AAWT01093310.1|) (- strand)1403 (gi|124163860|gb|AA WT01057958.1|) (+ strand) (locus 1)5146 (gi|124196975|gb|AA WT01024843.1|) (+ strand) (locus 2)2151 (gi|124194162|gb|AA WT01027656.1|) (+ strand) Cluster of C. sinensis was obtained from contig 2339 (gi|353340623|dbj|BADR02002339.1|) (- strand). Cluster of S. mansoni was obtained from chromosome W (gi|360043576|emb|HE601631.1|) (+ strand). Cluster of S. japonicum was obtained from contig S000054 (gi|227129020|emb|FN331028.1|) (- strand). Cluster of H. microstoma was obtained from contig 370001 (gi|528309775|emb|CBLW010002354.1|) (+ strand). Cluster of E. granulosus was obtained from contig 0003137 (gi|528307253|emb|CBLN010000400.1|) (- strand). Cluster of E. multilocularis was obtained from contig 007728 (gi|528775369|emb|CBLO010002090.1|) (- strand). Cluster of T. solium was obtained from contig 01703 (pathogen_TSM_contig_01703) (+ strand). Cluster of G. salaris was obtained from scaffold 7180006951238_l_32618_C_75.085_gc_34.438 (+ strand) (PDF) [file pntd.0003680.s001.pdf]

S1 Appendix. Alignment of miRNA clusters.

csi – *C. sinensis*, egr – *E. granulosus*, emu – E. multilocularis, gsa – *G. salaris*, hmi – *H. microstoma*, sja – *S. japonicum*, sma – *S. mansoni*, sme – *S. mediterranea*, tso – *T. solium*, sec. struc. – secondary structure. Mature miRNA sequences are in bold type and underlined. Clusters of S. mediterranea were obtained from contigs:

297 (gi|124128508|gb|AAWT01093310.1|) (- strand)

1403 (gi|124163860|gb|AA WT01057958.1|) (+ strand) (locus 1)

5146 (gi|124196975|gb|AA WT01024843.1|) (+ strand) (locus 2)

2151 (gi|124194162|gb|AA WT01027656.1|) (+ strand)

Cluster of *C. sinensis* was obtained from contig 2339 (gi|353340623|dbj|BADR02002339.1|) (- strand)

Cluster of *S. mansoni* was obtained from chromosome W (gi|360043576|emb|HE601631.1|) (+ strand)

Cluster of *S. japonicum* was obtained from contig S000054 (gi|227129020|emb|FN331028.1|) (- strand)

Cluster of *H. microstoma* was obtained from contig 370001 (gi|528309775|emb|CBLW010002354.1|) (+ strand)

Cluster of *E. granulosus* was obtained from contig 0003137 (gi|528307253|emb|CBLN010000400.1|) (- strand)

Cluster of *E. multilocularis* was obtained from contig 007728 (gi|528775369|emb|CBLO010002090.1|) (- strand)

Cluster of *T . solium* was obtained from contig 01703 (pathogen_TSM_contig_01703) (+ strand)

Cluster of *G. salaris* was obtained from scaffold 7180006951238_l_32618_C_75.085_gc_34.438 (+ strand)

```
miRNA name
sec. struc.
sme_miR-71c/2c            ----------------TAATATGTTTGT--TTAAGCT-TAGTAATTATTTCA-TTTCAAGGTAAA---T-TGTCATTTCTTTG-CCAAGAATCACATGTT 75
sme_miR-71a-1/2a-1_l1     ----------------TAAAATTATTGA--AAGCGCCATGTTGGTCATTTCT-TTGCCTGAAAAA---TATATGAATCATATAACTTTGAAT-GTGAATT 77
sme_miR-71a-1/2a-1_l2     ----------------TAAAATTATTGA--AAGCGCCATGTTGGTCATTTCT-TTGCCTGAAAAA---TATATGAATCATATAACTTTGAAT-GTGAATT 77
sme_miR-71a2/2a2          ACATTTTATAATTGTTTAAATTGTTCGAAGTAGCGCCATATTGGTCATTTCT-TTGCC---TAGA---T------TTTAT-TAACAAAGAAT------TT 80
gsa_miR-71a/2a            ----------------CAGAA--ATTGG---AATTTTTCCTCGGTCATTTTAATTGGCACGAAAACTCTATGTAGTTTTCTTCAC-ATGGAT-GTAAGTT 77
csi_miR-71a/2a/2b/2e      ----CCGTGCCCGCGGC-GTACGGTTCA-CGGTTGTCCCACGCGTGCGTCGCTGTACATTTCGTGG----T-ATGTCT-TGTTCCGTTTTAT-----TTT 83
sja_miR-71a/2a/2b/2e      -------------CAAC-ATAGCATTCA-TA--TGTTCTGGTCAGTTTTC-TTCTATATTTGGTAAGTCCT-TTGACTATATGTCGTATCAT-----ACG 76
sma_miR-71a/2a/2b/2e      -------------CGACCAAATCATGAA-C---TGCCCTGAACTTTTTTC-TTATTCATCTGGTAAGCACT-TTGTTTACTTGTCACACTGT-----TCA 76
egr_miR-71a/2c/2b         ----------GTATTGCGGTGCGGTC---TTGGTGTCGTCCT-ATCTCACGCTTTTTCCTTT-CATGTTTT-ATGCGTTTATA-CGCTTCAT-----GTT 78
emu_miR-71a/2c/2b         ----------GTATTGCGGTGCGGTC---TTGGTGTCGTCTT-ATATCACGCTTTTTTCTTT-TATGCTTT-GTGCGTTTATA-CGCTTCAT-----GTT 78
tso_miR-71a/2c/2b         ------------ATTGTGGGGTGGTC---GTTGTGTTGTGTT-TTCTGGTTCTTTTCCCTTCGCCTGCTTTCATGCTTTTATG-CGCCTGAT-----GTT 78
hmi_miR-71a/2c/2b         ----------ATGTTTTTCT-CAGTCAC-TTCGTGCGATCTTGACTTCTCGCTTATTCTTTTGCTT-TTTACTTGTTTTTGTA-AGTCTCGC-----TTT 81
                                                  *                                                         *

miRNA name                                           71a/71c(sme)
sec. struc.                                .((((((((..(((((((..(((((.(((-((------(........----.)))))).))))).-)))))))..)-))))-
sme_miR-71c/2c            TAGAAAGTTTATGTTGTA-GTTTTCT-TGAAAGACATGGGTAGTGAG-ATTATTTAAATTAAATATTTAAAATCTCATTATTCAAGTTTTTCTTATAAG- 171
sme_miR-71a-1/2a-1_l1     TA---ATTCAATTTTAAATGCATGAA-TGAAAGACACGGGTAGTGAG-AT-----AAGCTATATATC----ATCTCATTGCCAATGTCTTT----TAAA- 158
sme_miR-71a-1/2a-1_l2     TA---ATTCAATTTTAAATGCATGAA-TGAAAGACACGGGTAGTGAG-AT-----AAGCTATATATC----ATCTCATTGCCAATGTCTTT----TAAA- 158
sme_miR-71a2/2a2          TA----TTTTTTCTTGGA--TATAAC-TGAAAGACACGGGTAGTGAG-AT--------ATGGATTTTTC--ATCTCTATATTCGTGTCTTT----CATA- 157
gsa_miR-71a/2a            CTC--TGTTATTCTGATACGCTTGA--TGAAAGACATGGGTAATGAGTATC-----ATCTAAGT--------TCTCATTATTCCTGTCCTT----CATAA 156
csi_miR-71a/2a/2b/2e      T----------TATGGTATCCGCGCTGTGAAAGACGATGGTAGTGAG-AT------GATGAGTTT----CCATCTCCCTACCC-CGTCTTTTCC-GGCG- 159
sja_miR-71a/2a/2b/2e      CGA---AAAGATGTGATACTAGTGCTGTGAAAGACGATGGTAGTGAG-AT------GCCAG--TT----GCATCTCGCTTCCC-CGCCTTTCCC-GGTA- 157
sma_miR-71a/2a/2b/2e      TGT---GAAGATGTGATACTAGTGCTGTGAAAGACGATGGTAGTGAG-AT------GCCGA--TT----GCATCTCGCTTCCC-CGCCTTTCCC-GGTA- 157
egr_miR-71a/2c/2b         CGC---GCG--TTTGGCAGTCATCTCGTGAAAGACGATGGTAGTGAG-AT------ACAGAGGAATCGAGCATCTTGCTACTC-CGTCTTTTCCTAAGA- 164
emu_miR-71a/2c/2b         CGC---GCG--TTTGGCAGTCATCTCGTGAAAGACGATGGTAGTGAG-AT------ACAGAGGAATCGAGCATCTTGCTACTC-CGTCTTTTCCTAAGA- 164
tso_miR-71a/2c/2b         CCT---GTG--TTTGGCAGTCATCTCATGAAAGACGATGGTAGTGAG-AT------ACAGAAGAACCAAGCATCTTGCTACTC-CGTCTTTTCCTAAGG- 164
hmi_miR-71a/2c/2b         ACC---GTA-----AGCAGTTATCTTGTGAAAGACGATGGTAGTGAG-AT------ACAGAGGTTT---GCGTCTTGCTACAC-CGTCTTTTCATAAGA- 161
                                           *         ********   **** **** **                       ***   *      *   **

miRNA name                                                                                                         2a/2b(gsa)/2c(cestodes)
sec. struc.               --)))).......               ...-.((((((...------(((..((((((((((((((-(.(((----((....-----...----)-))))
sme_miR-71c/2c            --C-TACTATTTA--TCG-TCATTATTTCTTTTTCAAATACA-----AAAATCATCAATTTGGTCTGTTATAA----ATGGCT-----TT-------TAT 244
sme_miR-71a-1/2a-1_l1     --CGTGTTATTTA--ACGATCGTAATATCAGTT-CATTTATG-----TGAGTT-CCAGTGTGCTATGATGTAT----ATTTCTAAAAATTT-CTATATAT 242
sme_miR-71a-1/2a-1_l2     --CGTGTTATTTA--ACGATCGTAATATCAGTT-CATTTATG-----TGAGTT-CCAGTGTGCTATGATGTAT----ATTTCTAAAAATTTTCTATATAT 243
sme_miR-71a2/2a2          --T---TTATTCACGATGGTCATCATAATTGTT-CACTTAAT-----TGAGTT-CTACGGTGTTGTGATATA-----ATGATTA----TTT-----ATAT 231
gsa_miR-71a/2a            TTCGTTTCATTC----TG--CGTCAGA-CTGTT-CAATGATTGAACCTAAGTTGCAAGCG-GTTGTTATATAATTTCATTTTTA---ATTC--AATATAT 242
csi_miR-71a/2a/2b/2e      --TGGACCATGAG------TCATCATTTCCG-ATCTTTCACA------CCGCAGTCAATATTGGTTG-TAGGC----AATGCA-----ACT----T-TGT 229
sja_miR-71a/2a/2b/2e      --AGAATCACTAG------TCGTCAGTAAT--ACCTTTTGCA------CCGCAGTCAATATTGGCTG-ATGGC----ATTGTT-----TTA----T-TGT 226
sma_miR-71a/2a/2b/2e      --AGAATCACGAG------TCGTCTGCAAT--ACCTCTTGCG------CCGCAGTCAATATTGGCTG-AAGGC----ATTGCT-----TTA----T-TGT 226
egr_miR-71a/2c/2b         --TGCTGCTTTGA------CCATCCCGATTACGGGCGTTGT-------TCGTCGTCAACATTGCCTG-TAGAC----ACGGGT-----TGC----GATGT 235
emu_miR-71a/2c/2b         --TGCTGCTTTGA------CCATCCCGATTACGGGCGTTGT-------TCGTCGTCAACATTGCCTG-TAGAC----ACGGGT-----TGC----GATGT 235
tso_miR-71a/2c/2b         --TGCTGCTTTGA------CCGTCTCGACTGTGGGCGTTGT-------TCGTCGTCAACATTGCCTG-TAGAC----ATGGGT-----TGA----TATGT 235
hmi_miR-71a/2c/2b         --TGCTGCATTAA------TCGTCTGGAATATAGGCTTTGT-------TCGTCGTCAACATTGTCTG-TATAC----ATTGTT-----TCT----TATGT 232
                                              * *                                 *        *           *                   * *
```

```
miRNA name                 2a/2b(gsa)/2c(cestodes)
sec. struc.                )))))))))))))))...)))...))))))                            .....-------------(((--((((((...------.(((
sme_miR-71c/2c             CACAGCCAAAACTGATGATCTTATAAATTTGTC--AAACATCC-CAAT--TTAG-CATTTATG----------ATTTT----TAAACAAGGATTTTTATT 324
sme_miR-71a-1/2a-1_l1      CACAGCCCCGCTTGGAACGCTAATTAATGAAT---AATCATTCATAATATTTAT-TAATTTTG----------ATATT----TTAGAAGACGTTATCAAT 324
sme_miR-71a-1/2a-1_l2      CACAGCCCCGCTTGGAACGCTAATTAATGAAT---AATCATTCATAATATTTAT-TAATTTTG----------ATATT----TTAGAAGACGTTATCAAT 325
sme_miR-71a2/2a2           CACAGCCCCGCTTGGAACGCTAAAATATGAATGTTCATCATTAATTACATTGATATAGTTATACCACATGGCCATATTGGAATTGGTGAGTAT-GTTATC 330
gsa_miR-71a/2a             CACAGCCCTGCTTGGAACGC-AAGTTCTTTCTC--AATC-TTCTCGATCGTCCTCT--TTATA-----------CACT----CTATCGGA------TATT 315
csi_miR-71a/2a/2b/2e       CACAGCCAGTATTGATGAACGGGGCGAAGGACGC--AC-GTC---------CAC--CACTGCG-------------TTA--GGTGGTGCG------CGTC 294
sja_miR-71a/2a/2b/2e       CACAGCCAGTATTGATGAACGGGGTAATAGGCGC--AC-GTC---------CAC--CACTGC----------------A--GGTGGTGCG------CGTC 288
sma_miR-71a/2a/2b/2e       CACAGCCAGTATTGATGAACGGTGTAAAAGGCGC--AC-GTC---------CAC--CACTGC----------------A--GGTGGTGCG------CGTC 288
egr_miR-71a/2c/2b          CACAGCCAATATTGATGAACGAGTGGCGTCTTCT--ACCGTCGGTTTTTT-TGC--TCTTTTG-------------CT---GTCAGTCGG------TGTC 308
emu_miR-71a/2c/2b          CACAGCCAATATTGATGAACGAGTGGCGTCTTCT--ACCGTCGGTTCTTTCTGC--TCTTTTG-------------CT---GTCAGTCGG------TGTC 309
tso_miR-71a/2c/2b          CACAGCCAATATTGATGAACGAGTCACGTCTTTT--ACCATCGGTTTCCT-CGT--GGTTTTA-------------CT---GTCAGTCAG------TGTC 308
hmi_miR-71a/2c/2b          CACAGCCAATATTGATGAACGAGCTAAGTCTTAT--ACCATC----------AT--GACTTAT-------------CT---GTTGGTTCG------TGTT 296
                           *******     **     *                *   *                  *

miRNA name                                                 2b
sec. struc.                (((((((.-((((((.((((.......-))-)).)))))).)))..)))))))--....)))))))-))
sme_miR-71c/2c             TTAAAATTATTTT-AAAT-TT-CAGTAACACTAATAATGGAT--ATTGATGGTTGTTA-ATTAGTATGTATAAAA-------------------GATTAA 399
sme_miR-71a-1/2a-1_l1      TGGAA--TGTTTT-AA-T-TT-GAATA-CATAAATGGCTTTTTAACTGGTGTATTTTA-TTTATCA-GTTTAAAG--TATTTTAATTTAGTTTTGTTCAA 413
sme_miR-71a-1/2a-1_l2      TGGAA--TGTTTT-AA-T-TT-GAATA-CATAAATGGCTTTTTAACTGGTGTACTTTA-TTTATCA-GTTTAAAGAGTTTTTTAATTTAGTTTTGTTCAA 416
sme_miR-71a2/2a2           TTGAATTTATATT-AATTGTT-CAATA-TTTATCTAATTTT---ATTTTTGGA-GTTA-ATTTCCA---AAAAAAG----------------CCTATCAT 403
gsa_miR-71a/2a             CGAAAAATGTTTTGAAACTTT-CGAT--TTGAAATGTTCTT---ATTTAATTTTGCCATATTCGCA-AAAAAAAA-------------------TAGCAT 389
csi_miR-71a/2a/2b/2e       CCAAAGGA-CTGTGAGGCAGTGCCGTC-CT-GTATCACAGCCCTGCTTGGGAC--ACAGGCCACCT-AATTAAG-----------------------CGT 365
sja_miR-71a/2a/2b/2e       TCAAAGGA-CTGTGAGCCAACGTAATTACT-GTATCACAGCCCTGCTTGGGAC--ACAGCCTACCT-GCTTAGT-----------------------CAT 360
sma_miR-71a/2a/2b/2e       TCAAGGGA-CTGTGAAACATCGTAACCATTTGTATCACAGCCCTGCTTGGGAC--ACAGCCTACCT-GCTAAGA-----------------------CAT 361
egr_miR-71a/2c/2b          CCAAGGG--TGGTGATCTACT-CAGTGGGA-GTATCACAGCCCTGCTTGGGAC--ACATGCTGGCA-GCTCGAA-----------------------CGA 378
emu_miR-71a/2c/2b          CCAAGGG--TGGTGATCTACT-CAGTTGGA-GTATCACAGCCCTGCTTGGGAC--ACATGCTGGCA-GCTCAAA-----------------------CGA 379
tso_miR-71a/2c/2b          CCGAGGG--TGGTGATCTACT-TAGTCAGA-GTATCACAGCCCTGCTTGGGAC--ACATGCCGGCA-GCTCGAA-----------------------CGA 378
hmi_miR-71a/2c/2b          CCAAGGG--TCGTGACTTACT-TCGAATCA-GTATCACAGCCCTGCTTGGGAC--ACATGCCAGTA-GCCCAAC-----------------------CTT 366
                              *        * *                   *           *          *

miRNA name                                                                                     2e
sec. struc.                       ..------...((-(..(((((((((((.(((((...(((((.((...----...)).)))))...))))).)))..)-)))))))..)))..
sme_miR-71c/2c             ATAAATTCGATATA-TTGAAAATTCTAATTGGA--AAATATT----TTCAAATTGT------CTTTTATATGAAAATC--TTCG------GAATATGTG- 477
sme_miR-71a-1/2a-1_l1      ATTAATCTAGGGTAATCAATAATTATTTATGAA--CAATCTT----TT--ATTTGC------TTTTTAAATTAAATTC--TTTGTTTAAAGAAT-TATAT 496
sme_miR-71a-1/2a-1_l2      ATTAATCTAGGGTAATCAATAATTGTTTATGAA--CAATCTT----TT--ATTTGC------TTTTTAAATTAAATTC--TTTGTTTAAAGAAT-TAT-- 497
sme_miR-71a2/2a2           AATAATATCGTGCTTTT-----TTGTAACTAAC--TAATTTCGGTGTTTATTTTGCGGAAAGTCCTTAAACGTAATTCAATTTGAGCAAGGCAT-TTTCA 495
gsa_miR-71a/2a             CGTAGTTTAAAGTGAC--ATTTTTCTGAACGAATTCCGTCCTACGACTTGATTTATGCT---CCTTAAAACTATAGTC----CGTGTCCCGCCTAGCTG- 479
csi_miR-71a/2a/2b/2e       CGAAACCTC------CCACC-GTTCTTACCAACTTTGACTGCGTTATACTGTTCC----ATTGCTGTGTATCACAGTCCAAGCTT-TGGTAAGTTTGGGT 453
sja_miR-71a/2a/2b/2e       CATACTGTC------CCACC-GCTCTTACCAACTTAGACTGAGTTATACTGCTACTGTAAGCTTTGTATATCACAGTCCAAGCTT-TGGTAAGTTTTGAT 452
sma_miR-71a/2a/2b/2e       CTCTTTGTC------CCATC-GCTCTTACCAACTTTGACTGAGTTATACTGCTTTTGTAAGCCTTGTATATCACAGTCCAAGCTT-TGGTAAGTTTTGGT 453
egr_miR-71a/2c/2b          CCGAATTTCA-----CCACTTGTTTTGTCCGAG-ATGTCTGGTTTGT--AGGTC----------TGTAGAGTGCA-TCGGGGCTGGCGTTC-ACGCGGGT 458
emu_miR-71a/2c/2b          CCGAATTTCG-----CCACTTGTTTTGTCCAAG-ATGTCTGGTTTGT--AGGTC----------TGTAGGGTGCA-TTGGAGCTGGCGTTC-ACGCGGGT 459
tso_miR-71a/2c/2b          CCGGATTTTG-----CAATTTACTACTCCTGGACATGTCCGTTTTGT--TAGTA----------TATGGGGTGCA-TTGGAGTTGGTGGTT-GTT----- 454
hmi_miR-71a/2c/2b          CATCGCTT-------CTATTTGCTGTGTGCAA-----TCTAGATTGA--TGATTA-----CTCTTCTAAACCATA-CCAAGGTTG-TGGTT-GTATTCGT 444
                                                  *                            *                     *
```

```
miRNA name
sec. struc.
sme_miR-71c/2c             GAAATTTTAAATTGAT------TTGATAAATGTAAATATGGATTATTGCCAAAAATATTTTAAATGAAATTGTATTGAGA-----CCTTGAATGA-TATT 565
sme_miR-71a-1/2a-1_l1      GAAATAT--AATAGTT------TTAG--AATTTCAGAATGTGTTATG---AAAGACTCCTAAAATG----TATATTAACG-----CACGGAGTTACTGTT 574
sme_miR-71a-1/2a-1_l2      GAAATAT--AATAGTT------TTAG--AATTTCAGAATGTGTTATG---AAAGACGCCTAAAATG----TATATTAACG-----CACGGAGTTACTGTT 575
sme_miR-71a2/2a2           GGATTTT--AAGAG---------TGAGAAAGTTATATATATATTGTT-----TATTAATTAACATGCTTCTGC-TTAA--------TTTAAGTCAAAGTT 570
gsa_miR-71a/2a             AAAACAT--GATTCAA------TTATTAA---TGGACATATGCTAAC-CAATCATGAACTAAAATCA--TGATCTCATTG-----GTTCAATATATTTCT 560
csi_miR-71a/2a/2b/2e       GGGATGATCGCCAGTCG----GTTAGGCCGT----TGGTGCGCTGCTGTGGCG----TTCGCGCCGCCAGCCCACGGTTG-GCCTCCTCGTGCTAAC--- 537
sja_miR-71a/2a/2b/2e       GGGTTGATCGACAGCTGC--TGTTCAGTAATGAG-TCATATATTGATGTTACT----TTTACCTCTCAACCACAGCGTGATGTTTTTCTA-ATTGACTGT 544
sma_miR-71a/2a/2b/2e       GGGTTGATCGACAGCTGG--TTTTC-GTA-TATT-TGATA-GTTTTTGTTCCAAAAGTTTCTTTCTTCATT----CTTTA-GTCTTTTCA-GCGAACT-T 540
egr_miR-71a/2c/2b          AGGTTTTTGCTCTGTA---ACGCTGGGCATTGTT-TGGTCTGGGTTCTGAGTGCAGGCTCTCTT-----ACTTGT--TTG---GCCTCCAGGTTGGGCGC 544
emu_miR-71a/2c/2b          AGGTTTTTGCTCTGTA---ACGCTGGGCATTGTT-TGGTCTGGGTTCCGAGTGCAGGCTCTC-------ATCTGC--ATG---GCCTCCAGATTGGGCGC 543
tso_miR-71a/2c/2b          ---TTTTTGTGTCACA---GTACTGTGCCTTG---TAGTGTAATTCCTGAGTGCTGATCTCCGT-----TTCTGTGGTCG---GACGCTGTGGTTTGTGC 537
hmi_miR-71a/2c/2b          GAATCATTTCCTTGAATTCACTCTATCCGATACTATGGCTTTTTACCTAAGTATGGGTGTGATGAAGTGATTTGTGATTG-----CTTAATGTTAATTTA 539
                                                  *

miRNA name
sec. struc.
sme_miR-71c/2c             ----CAAGATTTA---------ATTTTTATTG------T-TAATAGGC--------GATAACA------------ 600
sme_miR-71a-1/2a-1_l1      ----TA----TTG---------ATTCGTATT---------TAA-------------GATTGCATA---------- 600
sme_miR-71a-1/2a-1_l2      ----TA----TTG---------ATTCGTATT---------TAA-------------GATTGCAT----------- 600
sme_miR-71a2/2a2           TCAGCAGCATACAT--------ATTATCATT---------TAG-------------GATA--------------- 600
gsa_miR-71a/2a             CCCATAAAAATCA----------TCAACATCC------AATAGT------------AATATCATTTTT------- 600
csi_miR-71a/2a/2b/2e       --CCGAGTCAACGCAGATATTCAGTG-CTTC-----CGCATGCT-GGTTGC---GCGACCCGTTTTTTGCCGCTA 600
sja_miR-71a/2a/2b/2e       TACGTTATGAATGC-GAATTTTGTTAACGTT-----CAT-TGTT-TGCTTC---GTATTCTTCAGTT-------- 600
sma_miR-71a/2a/2b/2e       TCCTCTGCCAATGCAGAACTACATCGTCTTC-----AGT-CATT-GGC------GAAATGGTGAGTTGATCGC-- 600
egr_miR-71a/2c/2b          CGCTGTGGGTTCGC-----CTGGGTGGCGTC---GTCGCTCGTTCCGCCTCTCGGCGTGCGA--AT--------- 600
emu_miR-71a/2c/2b          TGCTGTGGGTTCGC-----CTGGGTGGCGTC---GTCGCTCGTTCAGCTTC--GCTGTGTCA--ATGCT------ 600
tso_miR-71a/2c/2b          CGCCGTGTAGGCGG------TGGCTTGCCTTTGGGTTGTCTGTTCCGCTGG-CGGTGGCGGTCATCCGT------ 600
hmi_miR-71a/2c/2b          CACGTTTTTTTTGC-----TT---TATCTTT---GT-GATTATTAT-CCGGAGGGGGGGAGGGATTTCGGGGCT- 600
                                                        *
```

Clusters of S. mediterranea were obtained from contig 1374 (gi|124182360|gb|AAWT01039458.1|) (- strand)

Cluster of C. sinensis was obtained from contig 2957 (gi|353340005|dbj|BADR02002957.1|) (+ strand)

Cluster of S. mansoni was obtained from chromosome 5 (gi|350645965|emb|CABG01000032.1|) (- strand)

Cluster of S. japonicum was obtained from contig S000102 (gi|227129067|emb|FN331075.1|) (+ strand)

Cluster of G. salaris was obtained from scaffold 7180006953439_1_202915_C_79.960_gc_34.778 (- strand)

```
miRNA name
sec. struc.
sme_miR-71b/2d/752/13     TTCGA--AATTTTCGCTGAGTAAG---AGTTTGAATTTGTT-TGTATA-TA-------TATGCAATTTTATCTTGTAGATTAGCATTTTGTTGTGTAGGT 86
gsa_miR-71b/2c/2a/2d      CCATGCC-ATTGCTTATAA-CAGAC--ACCA---ATTCATCCTCCATGCTG-CTCCACGATTAAAATCCATGGTGTA-ATCATCGGCCCATATGATCC-- 89
csi_miR-71b/2f/2d/2c      TTAGGCCGGCTGGTC--GATCCGGTATAACATGTGGCCATTGTGTGATCTGTCG---TTGTTTTGGTCCCTCTTGT---CTGCCACTTTTTGATGTTG-- 90
sja_miR-71b/2f/2d/2c      --TGGTTGATAGTTTTAAAGCAAAT-TAGTATATAGTCATTGCGAAGGGTGGCGTGACCATTCGTGTGCGTCATGC---TTGCTGGTCTGTCTAGT---- 90
sma_miR-71b/2f/2d/2c      -TTGGTTAATCATTTTTGACCAGAT-TAGTATATAGGC-TTCTTAAGGGTGGCGAGACTGGTGAAGTGCGCCATGT---CCCTTGGTCTGTCTAGT---- 90
                                            *        *           *         *                *      **               *    *

miRNA name                               71b
sec. struc.                   ..(..-(((((((((((((((.((((.((((.((((.((----((...--...))))..)))).))))))))))))))))))))))-)..)
sme_miR-71b/2d/752/13     GATTCAGTACT-CTTTGAAAGACACAGGTAGTGGGACAGATAT----TATCTAATAAAAGCTTGTC--CTTCTAATGTGTTTTTCGAAAA-CCATTGAAT 178
gsa_miR-71b/2c/2a/2d      ----CGATGATAGTGTGAAAGACTTGCGTAGTGAGACGCTC------TAAAT---GTGCAT--AGCGTCTTCTATCAAGACTTTCGTACCATCGTTCGTC 174
csi_miR-71b/2f/2d/2c      ----CCGTG-TGTCGTGAAAGACTTGAGTAGTGAGACGCTCAT----TTGCT--CATGGATTCGGCGCCTCATACTGAGTCTTTCGTGAT-GTGCCGGCT 178
sja_miR-71b/2f/2d/2c      ----CCGTT-TGCTGTGAAAGACTTGAGTAGTGAGACGCTTATAGCGTTGTTGTCTGCGGTTCGGCGCCTCATACTAAGTCTTTCCCGGC-TCACTGGCC 184
sma_miR-71b/2f/2d/2c      ----CCGTT-TGCCATGAAAGACTTGAGTAGTGAGACGCTTACAGCGTGGTT-TTTTCCCTTCGGCGCCTCATACTGAGTCTTTCCCGGC-TCACTGGCC 183
                              *  *  *    ********    ****** ***          *   *             *  **  **    *  ****

miRNA name                                                                                              2c(gsa)/2d(sme)/2f(trematodes)
sec. struc.                                                                634 ...((..(-(.(((..-----.((((..(((((.(((..(((((....)))..))..))
sme_miR-71b/2d/752/13     CAATCATCAGTTCACTAGTGA-CATTTTGCCTCTTACG-GACATCATTATTTGACTGTCGATAGATATTGGTAACAT-----TATTTTGGTA----GTAT 267
gsa_miR-71b/2c/2a/2d      CGTACGTCTTTTCAGAGATA--AATTCTACCATAAACTTGGCATTAATCATTGGCTGC----AAATAATGTAAGTAG-----CAAA-----------TGT 252
csi_miR-71b/2f/2d/2c      GATGCGTC-GTCCCCAGTCC--AATGCT---TGATGTCCGGTATTGATT-CTGGCTG-----AGATAGCGGCTGGGATTAGCTGCGCAAGCAAAGCCTAT 266
sja_miR-71b/2f/2d/2c      TAACCGTCTGATGTGAATTTGTAATGCTGCACGATACCTGGTGTTGATT-CTGGCTG-----AGTTCGTGGCAGGA------TAAA-GGTT--TGCCTGT 269
sma_miR-71b/2f/2d/2c      TAACCGTCTGAGAAGGATTCGTAATGCTGCACGATACCTGGTGTTGATT-CTGGCTG-----AGATTGAGGCAGGAA---AATAAACAATTGTTGCCTGT 274
                              * **               **  *           *   *   *   ** ***     *  *   *                           * *

miRNA name                2c(gsa)/2d(sme)/2f(trematodes)
sec. struc.               ).)))))..))))...))).))...))....                              .....-(((((((((---((((...(---(.(((((((.
sme_miR-71b/2d/752/13     CACAGCCAAATTTGATGTCCTAAATAAGGTAGTCACCTTCATT------------GATTATAGTTATCTAATTTGAAAACCTCCAAA-------GTGATT 348
gsa_miR-71b/2c/2a/2d      CACAGCCAATCTTGATGTCAAGTTGTTGGCATTGAACTAC---------------AGTCCCATCTA-TTAGG--AC---CGTCCGAGTGCGACTGTGATG 331
csi_miR-71b/2f/2d/2c      CACAGCCAATATTGATGCCCGACAGCATGCACCTGACATCTAGCGTCTCTTTGGAAGTGCCAGCAC-CGGGTTTGT---CATCGATG---GCCTGTGATT 359
sja_miR-71b/2f/2d/2c      CACAGCCAATATTGATACCCGGTGTTGGGCACTTAACTTCTAA----------GATGCCGCCGCGA-CCAGTTCGT---CATCCATG---GATTGTGATT 352
sma_miR-71b/2f/2d/2c      CACAGCCAATATTGATACCCGGCGTTGGGCACTTGACTTCTAA----------GATGTAACCGCGA-CCAGTTCGT---CATCCTTG---GATTGTGATT 357
                          *********  *****  *         * *     *  *                                       * **           *****
```

```
miRNA name                                     752(sme)/2a(gsa)/2d(trematodes)
sec. struc.               ..--((((....))))..-------...---))))))).)).......))))))--))-))))).. 
sme_miR-71b/2d/752/13     GT--GAATAAAAGTTTTATT-----AATTTAATCACAGTCA-GCATTGGTGGTTTGAAAGTAGAGTGATCTTACAATCGAATCAATTGTGTTTGGAAAGC 440
gsa_miR-71b/2c/2a/2d      TT---ATGTCATGTTTGC-------AAT---ATCACAGTCCTGCTTTGGTGAC--GGTGCAATGATGGTA-TACAGC---ACCGC---TGTATTAC-AGC 408
csi_miR-71b/2f/2d/2c      TT--GAGCACATGTTTCT-------AAT---ATCACAGTCCTGCTTAGGTGAC--GA-ACCTGTCTGGT--TG-TGC---GTCGA---TATTCCACCGGT 435
sja_miR-71b/2f/2d/2c      TTCGGACTCCATGTTTCATTAAGTGAAT---ATCACAGTCCTGCTTAGGTGAC--GA-ATTTCGTTGGT--TA-GTC---TTCA----TA--CCA--GGT 432
sma_miR-71b/2f/2d/2c      TTCCGAAATAAAATTTTATGAATAGAAT---ATCACAGTCCTGCTTAGGTGAC--GA-ATTTCGCTGGT--TA-ACC---TTCG----CAAACCA--GGT 439
                           *   *    *  ***         ***   *********  ** * ****    *         ** *  *          *               *

miRNA name                                                                          13(sme)/2d(gsa)/2c(trematodes)
sec. struc.                  ...(-----(((((.((.-------..((((((..((.((((((.((((.........)---))).))))))).))....)-))))))...))))))
sme_miR-71b/2d/752/13     AACAT-----------AATGG------GAGCCTTTGG-CGGCTGTGATATG--ATAATTATATA--CATATCACAGTCATGCTA-AAGAGCTT-----GT 512
gsa_miR-71b/2c/2a/2d      AGCATCTTGCTTAACTGGTGTCTTCATAAACCCTTGCTCGGGTGTGATGTG--TCTAATGATTAATCATATCACAGCCGTGCTTTAAGGGCTTTTGATGC 506
csi_miR-71b/2f/2d/2c      GGCAGCT-----ACCGAGCAC-------ACCCCTTGTTCGACTGTGAAATGCCTTCGTTGTAG---CATATCACAGCCGTGCTT-AAGGGCTTTGTCGGT 519
sja_miR-71b/2f/2d/2c      TTCAG------------GCGC-------AACCCTTGTTCGACTGTGATGTG--ATTGAAATTAGCCCATATCACAGCCGTGCTT-AAGGGCTTCGTCGAA 510
sma_miR-71b/2f/2d/2c      TGTTG------------GCGC-------ATCCCTTGTTCGACTGTGATGTG--TTCGAAGTAAACTCATATCACAGCCGTGCTT-AAGGGCTTCGTCAAA 517
                                                      * ** ***  **   *****  **               ********** * ****  *** ****

miRNA name
sec. struc.               )
sme_miR-71b/2d/752/13     A----AATTGTTACTAATCGTCTCTCGTGCTTCAA----TGATCAGTTTGTCAATTTTAATAATAGATGCGAATTTTGTGTGC-AAAGTTTTATATA 600
gsa_miR-71b/2c/2a/2d      ACCATATACTTCATTAACTGCAAAGTGTTACAAAAAAATAAATTTATTTAACAAAATTTGTCTTTAACACAATCATGTAGTTCTAGAAAGTCAC--- 600
csi_miR-71b/2f/2d/2c      AGTTCGAACGTCGTTTTCATCA--TTATGCTTGAT------ATTTGTGC--TCA--TCTGCAACTCGAAGCGTTTGGCTGTGGGCGTATCCTG---- 600
sja_miR-71b/2f/2d/2c      ACCCGATTCAGCATTTTTGGTAAATTTTTATTTATATTGTCAGTTGTACAACAA--TCTGTTTCATTGAA--TTTTAATCTGCTTGTA--TTGGAT- 600
sma_miR-71b/2f/2d/2c      ACTCTGAACAACACATGTGATATATAGTGGCTTAT-----TA--CATGCAAGTA--TCTGCGTTTTTC----TTTCGTTCTTGTCATACATTGCAG- 600
                          *                          *     *       *    *      *  *                       *
```
