# Supplementary material for: Identification of microRNA Genes in Three Opisthorchiids
Source: PLoS Negl Trop Dis. 2015 Apr 21;9(4):e0003680. doi: 10.1371/journal.pntd.0003680 (PMC4405270; doi:10.1371/journal.pntd.0003680)
Supplement: S2 Appendix — csi—C. sinensis. Secondary structure of miRNA precursors is indicated by a parentheses and dots. Mature miRNA sequences including miR-2f are in bold type and underlined. (PDF) [file pntd.0003680.s002.pdf]

S2 Appendix. Position of primers target regions in clusters.
csi – *C. sinensis*. Secondary structure of miRNA precursors is indicated by a parentheses and dots. Mature miRNA sequences including miR-2f are in bold type and underlined.

csi_miR-71a/2a/2b/2e

>>>>>>>>>>Cl1-F>>>>>>>>>>

.((((((((.
AAAGATATTTTCGCAAGTGATCAATGTTTTCCTCAGTCATTTTATTTGTCCGTGCCCGCGGCGTACGGTTCACGGTTGTCCCACGCGTGCGTCGCTGTACATTTCGTGGTATGTCTTGTTCCGTTTTATTTTTTATGGTATCCGCGCTG**T** 150

>>>>clust1-for1>>>>>

.(((((((..(((((.((((((.........)))))).))))).)))))))..))))))))........ .....(((((...(((..((((((((((((((((.(((((.......)))))))))))))))))))))...)))
GAAAGACGATGGTAGTGAGATGATGAGTTTCCATCTCCCTACCCCGTCTTTTCCGGCGTGGACCATGAGTCATCATTTCCGATCTTTCACACCGCAGTCAATATTGGTTGTAGGCAATGCAACTTTG**TCACAGCCAGTATTGATGAACGG** 300

>>>>clust1-for2>>>>

...)))))) .....(((((((((....((((((((((.((((((.((((.......)))).)))))).)))..))))))))....))))))))) .....(((..((((((((((.(((((..
GGCGAAGGACGCACGTCCACCACTGCGTTAGGTGGTGCGCGTCCCAAAGGACTGTGAGGCAGTGCCGTCCT**GTATCACAGCCCTGCTTGGGACACA**GGCCACCTAATTAAGCGTCGAAACCTCCCACCGTTCTTACCAACTTTGACTGCG 450

.(((((.((......)).)))))...))))).)))..))))))))..)))..
TTATACTGTTCCATTGCTGTG**TATCACAGTCCAAGCTTTGGT**AAGTTTGGGTGGGATGATCGCCAGTCGGTTAGGCCGTTGGTGCGCTGCTG 542

<<<<<clust1-rev<<<< <<<<<<Cl1-R<<<<<<

csi_miR-71b/2f/2d/2c

>>>>>>>clust2-for>>>>>>>

..(..((((((((((((((.((((.((((.((((.((((......))))..)))).))))))))))))))))))))))..) ...((..((.(((...((((..(((((
CCGTGTGTCG**TGAAAGACTTGAGTAGTGAGA**CGCTCATTTGCTCATGGATTCGGCGCCTCATACTGAGTCTTTCGTGATGTGCCGGCTGATGCGTCGTCCCCAGTCCAATGCTTGATGTCCGGTATTGATTCTGGCTGAGATAGCGGCTG 150

.(((..(((((....))..)))..))).))))))..))))...))).))...)).... .....(((((((((((((...((.(((((((...((((....)))).....))))))).))..
GGATTAGCTGCGCAAGCAAAGCCTA**TCACAGCCAATATTGATGCC**CGACAGCATGCACCTGACATCTAGCGTCTCTTTGGAAGTGCCAGCACCGGGTTTGTCATCGATGGCCTGTGATTTTGAGCACATGTTTCTAA**TATCACAGTCCTG** 300

<<<<<<<

....))))))))))))).. ...((((((.((...((((((..((.((((((.((((..........)))).))))))).))....))))))...))))))))
CTTAGGTGACGAACCTGTCTGGTTGTGCGTCGATATTCCACCGGTGGCAGCTACCGAGCACACCCCTTGTTCGACTGTGAAATGCCTTCGTTGTAG**CATATCACAGCCGTGCTTAAGGG**CTTTGTCGGTA 430

<clust2-rev<
