# Supplementary material for: Identification of microRNA Genes in Three Opisthorchiids
Source: PLoS Negl Trop Dis. 2015 Apr 21;9(4):e0003680. doi: 10.1371/journal.pntd.0003680 (PMC4405270; doi:10.1371/journal.pntd.0003680)
Supplement: S3 Appendix — sec. struc.—secondary structure. Mature miRNA sequences are in bold type and underlined. (PDF) [file pntd.0003680.s003.pdf]

S3 Appendix. Alignment of miR-71a/2a/2b/2e miRNA cluster fragment containing miR-2a, miR-2b and miR-2e.

sec. struc. – secondary structure. Mature miRNA sequences are in bold type and underlined.

```
miRNA name        miR-2a
sec. struc.       )))))))))))))))))...)))...))))))             .....(((((((((.
C. sinensis       TCACAGCCAGTATTGATGAACGGGGCGAAGGACGCACGTCCACCACTGCGTTAGGTGGTG 60
O. felineus       TCACAGCCAGTATTGATGAACGGGGCGAAGGACGCACGTCCACCACTGCGTTAGGTGGTG 60
O. viverrini      TCACAGCCAGTATTGATGAACGGGGCGAAGGACGCACGTCCACCACTGCGTTAGGTGGTG 60
S. japonicum      TCACAGCCAGTATTGATGAACGGGGTAATAGGCGCACGTCCACCACTGC---AGGTGGTG 57
S. mansoni        TCACAGCCAGTATTGATGAACGGTGTAAAAGGCGCACGTCCACCACTGC---AGGTGGTG 57
                  *********************** *  *  * *****************   ********

miRNA name                                             miR-2b
sec. struc.       ...((((((((((.((((((.((((......--.)))).))))))).)))..))))))..
C. sinensis       CGCGTCCCAAAGGACTGTGAGGCAGTGCCGT--CCTGTATCACAGCCCTGCTTGGGACAC 118
O. felineus       CGCGTCCCAAGGGACTGTGAGGCAGTGCCAT--CCTGTATCACAGCCCTGCTTGGGACAC 118
O. viverrini      CGCGTCCCAAAGGACTGTGAGGCAGTGCCAT--CCTGTATCACAGCCCTGCTTGGGACAC 118
S. japonicum      CGCGTCTCAAAGGACTGTGAGCCAACGTAATTACT-GTATCACAGCCCTGCTTGGGACAC 116
S. mansoni        CGCGTCTCAAGGGACTGTGAAACATCGTAACCATTTGTATCACAGCCCTGCTTGGGACAC 117
                  ****** *** *********  **  *         ************************

miRNA name
sec. struc.       ..)))))))))               .....(((..(((((((((((.(((((...((((
C. sinensis       AGGCCACCTAATTAAGCGTCGAAACCTCCCACCGTTCTTACCAACTTTGACTGCGTTATA 178
O. felineus       AGGCCACCTAATTAATCGTCGAAACCTCCCACCGTTCTTACCAACTTTGACTGCGTTATA 178
O. viverrini      AGGCCACCTAATTAAGCGTCGAAACCTCCCACCGTTCTTACCAACTTTGACTGCGTTATA 178
S. japonicum      AGCCTACCTGCTTAGTCATCATACTGTCCCACCGCTCTTACCAACTTAGACTGAGTTATA 176
S. mansoni        AGCCTACCTGCTAAGACATCTCTTTGTCCCATCGCTCTTACCAACTTTGACTGAGTTATA 177
                  ** * ****  * *  * **      ***** ** ************ ***** ******

miRNA name                            miR-2e
sec. struc.       (.((......)----).)))))...))))).)))..)))))
C. sinensis       CTGTTCCATTG----CTGTGTATCACAGTCCAAGCTTTGGT 215
O. felineus       CTGTTCCATTG----CTGTGTATCACAGTCCAAGCTTTGGT 215
O. viverrini      CTGTTCCATTG----CTGTGTATCACAGTCCAAGCTTTGGT 215
S. japonicum      CTGCTACTGTAAGCTTTGTATATCACAGTCCAAGCTTTGGT 217
S. mansoni        CTGCTTTTGTAAGCCTTGTATATCACAGTCCAAGCTTTGGT 218
                  *** *    *      *** *********************
```

Supplemental file 11: alignment of miR-71b/2f/2d/2c miRNA cluster fragmen containing miR-71a, miR-2f and miR-2d

sec. struc. – secomd structure

Mature miRNA sequences including miR-2f are in bold type and underlined

```
miRNA name        miR-71b
sec. struc.       (((((((((((.((((.((((.((((.((-------((......))))..)))).)))))))
C. sinensis       TGAAAGACTTGAGTAGTGAGACGCTCAT-------TTGCTCATGGATTCGGCGCCTCATA 53
O. felineus       TGAAAGACTTGAGTAGTGAGACGCTCAT-------TTACTCGCGGATTCCGCGCCTCATA 53
O. viverrini      TGAAAGACTTGAGTAGTGAGACGCTCAT-------TTGCTCACGGTTTCGGCGCCTCATA 53
S. japonicum      TGAAAGACTTGAGTAGTGAGACGCTTATAGCGTTGTTGTCTGCGG-TTCGGCGCCTCATA 59
S. mansoni        TGAAAGACTTGAGTAGTGAGACGCTTACAGCGTGGTTTTTTCCC--TTCGGCGCCTCATA 58
                  ************************* *        **         *** **********

miRNA name
sec. struc.       )))))))))))))))))..)
C. sinensis       CTGAGTCTTTCGTGATGTGCCGGCTGATGCGTC-GTCCCCAGTCC--AATGCT---TGAT 107
O. felineus       CTGAGTCTTTCGTGATGTGCCGGCTGATGCGTC-GTCCCCAGTCC--AATGCT---TGAT 107
O. viverrini      CTGAGTCTTTCGTGATGTGCCGGCTGATGCGTC-GTCCCCAGTCC--AATGCT---TGAT 107
S. japonicum      CTAAGTCTTTCCCGGCTCACTGGCCTAACCGTCTGATGTGAATTTGTAATGCTGCACGAT 119
S. mansoni        CTGAGTCTTTCCCGGCTCACTGGCCTAACCGTCTGAGAAGGATTCGTAATGCTGCACGAT 118
                  ** ********  *     * ***  *  **** *       *    ******    ***

miRNA name                                                                  miR-2f
sec. struc.             ...((..((.(((...((((..(((((.(((..(((((....))..)))..)))
C. sinensis       GTCCGGTATTGATTCTGGCTGAGATAGCGGCTGGGATTAGCTGCGCAAGCAAAGCCTATC 167
O. felineus       GTCCGGTATTGATTCTGGCTGAGATAGCGGCTGGGATTAGCTGCGCAAGCAAAGCCTATC 167
O. viverrini      GTCCGGTATTGATTCTGGCTGAGATAGCGGCTGGGATTAGCTGCGCAAGCAAAGCCTATC 167
S. japonicum      ACCTGGTGTTGATTCTGGCTGAGTTCGTGGCAGGA------TAAA-GGTT--TGCCTGTC 170
S. mansoni        ACCTGGTGTTGATTCTGGCTGAGATTGAGGCAGGAA---AATAAACAATTGTTGCCTGTC 175
                    * *** *************** * * *** **       *           **** **

miRNA name        miR-2f
sec. struc.       .)))))..))))...))).))...))....
C. sinensis       ACAGCCAATATTGATGCCCGACAGCATGCACCTGACATCTAGCGTCTCTTTGGAAGTGCC 227
O. felineus       ACAGCCAATATTGATGCCCGACAGCATGCACCTGACATCTAGCGTTTTTTTGGAAGTACC 227
O. viverrini      ACAGCCAATATTGATGCCCGACAGCATGCACCTGACATCTAGCGTTTCTTTAGAAGTACC 227
S. japonicum      ACAGCCAATATTGATACCCGGTGTTGGGCACTTAACTTCTA----------AGATGCCGC 220
S. mansoni        ACAGCCAATATTGATACCCGGCGTTGGGCACTTGACTTCTA----------AGATGTAAC 225
                  *************** ****       **** * ** ****           ** *   *

miRNA name                                                                  miR-2d
sec. struc.       .....(((((((((((((...((.(((((((...--((((....))))..-------...
C. sinensis       AGCACCGGGTTTGTCATCGATGGCCTGTGATTTT--GAGCACATGTTTCT-------AAT 278
O. felineus       AGCACCGGGTTTGTCATCGGTGGCCTGTGATTTT--GAGCACATGTTTCT-------AAT 278
O. viverrini      AGCACCCGGTTTGTCATCGATGGCCTGTGATTTT--GAGCACATGTTTCT-------AAT 278
S. japonicum      CGCGACCAGTTCGTCATCCATGGATTGTGATTTTCGGACTCCATGTTTCATTAAGTGAAT 280
S. mansoni        CGCGACCAGTTCGTCATCCTTGGATTGTGATTTTCCGAAATAAAATTTTATGAATAGAAT 285
                   **  *  *** ******  ***  *********  **    *  ***         ***

miRNA name        miR-2d
sec. struc.       ))))))).))......)))))
C. sinensis       ATCACAGTCCTGCTTAGGTGA 299
O. felineus       ATCACAGTCCTGCTTAGGTGA 299
O. viverrini      ATCACAGTCCTGCTTAGGTGA 299
S. japonicum      ATCACAGTCCTGCTTAGGTGA 301
S. mansoni        ATCACAGTCCTGCTTAGGTGA 306
                  *********************
```
