# Supplementary material for: Identification of microRNA Genes in Three Opisthorchiids
Source: PLoS Negl Trop Dis. 2015 Apr 21;9(4):e0003680. doi: 10.1371/journal.pntd.0003680 (PMC4405270; doi:10.1371/journal.pntd.0003680)
Supplement: S4 Appendix — sec. struc.—secondary structure. Mature miRNA sequences are in bold type and underlined. (PDF) [file pntd.0003680.s004.pdf]

S4 Appendix. Alignment of miR-71a/2a/2b/2e miRNA cluster.

sec. struc. – secondary structure. Mature miRNA sequences are in bold type and underlined.

```
miRNA name                                                   miR-71a
sec. struc.                              .(((((((((..(((((((..(((((.((((((....
C. sinensis       --TCCGTTTTATTTTTT-ATGGTATCCGCGCTGTGAAAGACGATGGTAGTGAGATGATGA 57
O. felineus       ---GCTTTTTATTTTTTTATGGTATCCGCGCTGTGAAAGACGATGGTAGTGAGATGATGA 57
O. viverrini      --TGCGTTTTATTTTTT-ATGGTATTCGCGCTGTGAAAGACGATGGTAGTGAGATGATGA 57
S. japonicum      CATACGCGAAAAGAT---GTGATACTAGTGCTGTGAAAGACGATGGTAGTGAGATGCCAG 57
S. mansoni        TGTTCATGTGAAGAT---GTGATACTAGTGCTGTGAAAGACGATGGTAGTGAGATGCCGA 57
                      *     *   *    ** **   * ***************************

miRNA name
sec. struc.       .....)))))).))))).)))))))..)))))))))........       .....((((
C. sinensis       GTTTCCATCTCCCTACCCCGTCTTTTCCGGCGTGGACCATGAGTCATCATTTCCGATCTT 117
O. felineus       GTTTCCATCTCCCTACCCCGTCTTTTCCGGCGTGGACCATGAGTCATCATTTCCGATCTT 117
O. viverrini      GTTTCCATCTCCCTACCCCGTCTTTTCCGGCGTGGACCATGAGTCATCATTTCCGATCTT 117
S. japonicum      --TTGCATCTCGCTTCCCCGCCTTTCCCGGTAAGAATCACTAGTCGTCAGTAAT-ACCTT 114
S. mansoni        --TTGCATCTCGCTTCCCCGCCTTTCCCGGTAAGAATCACGAGTCGTCTGCAAT-ACCTC 114
                    ** ****** ** ***** **** ****   * * **  **** **       * **

miRNA name                                                           miR-2a
sec. struc.       ((...(((..(((((((((((((((.(((((.......))))))))))))))))))))..
C. sinensis       TCACACCGCAGTCAATATTGGTTGTAGGCAATGCAACTTTGTCACAGCCAGTATTGATGA 177
O. felineus       TCACACCGCAATCAATATTGGTTGTAGGCAATGCAACTTTGTCACAGCCAGTATTGATGA 177
O. viverrini      TCACACCGCAGTCAATATTGGTTGTAGGCAGTGCAATTTTGTCACAGCCAGTATTGATGA 177
S. japonicum      TTGCACCGCAGTCAATATTGGCTGATGGCATTGTTTTATTGTCACAGCCAGTATTGATGA 174
S. mansoni        TTGCGCCGCAGTCAATATTGGCTGAAGGCATTGCTTTATTGTCACAGCCAGTATTGATGA 174
                  *  * ***** ********** **  **** **     **********************

miRNA name
sec. struc.       .)))...))))))             .....(((((((((....((((((((((.(((((
C. sinensis       ACGGGGCGAAGGACGCACGTCCACCACTGCGTTAGGTGGTGCGCGTCCCAAAGGACTGTG 237
O. felineus       ACGGGGCGAAGGACGCACGTCCACCACTGCGTTAGGTGGTGCGCGTCCCAAGGGACTGTG 237
O. viverrini      ACGGGGCGAAGGACGCACGTCCACCACTGCGTTAGGTGGTGCGCGTCCCAAAGGACTGTG 237
S. japonicum      ACGGGGTAATAGGCGCACGTCCACCACTGC---AGGTGGTGCGCGTCTCAAAGGACTGTG 231
S. mansoni        ACGGTGTAAAAGGCGCACGTCCACCACTGC---AGGTGGTGCGCGTCTCAAGGGACTGTG 231
                  **** *  *  * *****************   ************** *** ********

miRNA name                          miR-2b
sec. struc.       (.((((......--.)))).)))))).)))..)))))))....)))))))))
C. sinensis       AGGCAGTGCCGT--CCTGTATCACAGCCCTGCTTGGGACACAGGCCACCTAATTAAGCGT 295
O. felineus       AGGCAGTGCCAT--CCTGTATCACAGCCCTGCTTGGGACACAGGCCACCTAATTAATCGT 295
O. viverrini      AGGCAGTGCCAT--CCTGTATCACAGCCCTGCTTGGGACACAGGCCACCTAATTAAGCGT 295
S. japonicum      AGCCAACGTAATTACT-GTATCACAGCCCTGCTTGGGACACAGCCTACCTGCTTAGTCAT 290
S. mansoni        AAACATCGTAACCATTTGTATCACAGCCCTGCTTGGGACACAGCCTACCTGCTAAGACAT 291
                  *  **  *         ************************** * ****  * *  * *

miRNA name
sec. struc.              .....(((..(((((((((((.(((((...(((((.((......)----).))
C. sinensis       CGAAACCTCCCACCGTTCTTACCAACTTTGACTGCGTTATACTGTTCCATTG----CTGT 351
O. felineus       CGAAACCTCCCACCGTTCTTACCAACTTTGACTGCGTTATACTGTTCCATTG----CTGT 351
O. viverrini      CGAAACCTCCCACCGTTCTTACCAACTTTGACTGCGTTATACTGTTCCATTG----CTGT 351
S. japonicum      CATACTGTCCCACCGCTCTTACCAACTTAGACTGAGTTATACTGCTACTGTAAGCTTTGT 350
S. mansoni        CTCTTTGTCCCATCGCTCTTACCAACTTTGACTGAGTTATACTGCTTTTGTAAGCCTTGT 351
                  *      ***** ** ************ ***** ********* *    *      ***

miRNA name         miR-2e
sec. struc.       )))...)))))).)))..)))))))..)))..
C. sinensis       GTATCACAGTCCAAGCTTTGGTAAGTTTGGGTGGGATGATCGCC 395
O. felineus       GTATCACAGTCCAAGCTTTGGTAAGTTTGGGTGGGATGATCGCC 395
O. viverrini      GTATCACAGTCCAAGCTTTGGTAAGTTTGGGTGGGATGATCGCC 395
S. japonicum      ATATCACAGTCCAAGCTTTGGTAAGTTTTGATGGGTTGATCGAC 394
S. mansoni        ATATCACAGTCCAAGCTTTGGTAAGTTTTGGTGGGTTGATCGAC 395
                   *************************** * **** ****** *
```
