# Supplementary material for: Identification of microRNA Genes in Three Opisthorchiids
Source: PLoS Negl Trop Dis. 2015 Apr 21;9(4):e0003680. doi: 10.1371/journal.pntd.0003680 (PMC4405270; doi:10.1371/journal.pntd.0003680)
Supplement: S5 Appendix — csi—C. sinensis, sma—S. mansoni, hmi—H. microstoma, egr—E. granulosus, emu—E. multilocularis, tso—T. solium, sec. struc.—secondary structure. Mature miRNA sequences are in bold type and underlined. Mature miRNA sequences are in bold type and underlined. Cluster of C. sinensis was obtained from scaffold 198 (gi|353342189|dbj|BADR02000773.1|) (- strand). Cluster of S. mansoni was obtained from chromosome W (gi|360043576|emb|HE601631.1|) (- strand). Cluster of H. microstoma was obtained from contig 880003 (gi|528308588|emb|CBLW010003541.1|) (- strand). Cluster of E. granulosus was obtained from contig 00137 (gi|528306514|emb|CBLN010001139.1|) (+ strand). Cluster of E. multilocularis was obtained from contig 007760 (gi|528775363|emb|CBLO010002096.1|) (+ strand). Cluster of T. solium was obtained from contig 00015 (pathogen_TSM_contig_00015) (+ strand). (PDF) [file pntd.0003680.s005.pdf]

S5 Appendix. Alignment of miRNA clusters like regions.

csi – *C. sinensis*, sma – *S. mansoni*, hmi – *H. microstoma*, egr – *E. granulosus*, emu – *E. multilocularis*, sec. struc. – secondary structure

Mature miRNA sequences are in bold type and underlined.

Cluster of *C. sinensis* was obtained from scaffold 198 (gi|353342189|dbj|BADR02000773.1|) (- strand)

Cluster of *S. mansoni* was obtained from chromosome W (gi|360043576|emb|HE601631.1|) (- strand)

Cluster of *H. microstoma* was obtained from contig 880003 (gi|528308588|emb|CBLW010003541.1|) (- strand)

Cluster of *E. granulosus* was obtained from contig 00137 (gi|528306514|emb|CBLN010001139.1|) (+ strand)

Cluster of *E. multilocularis* was obtained from contig 007760 (gi|528775363|emb|CBLO010002096.1|) (+ strand)

Cluster of *T. solium* was obtained from contig 00015 (pathogen_TSM_contig_00015) (+ strand)

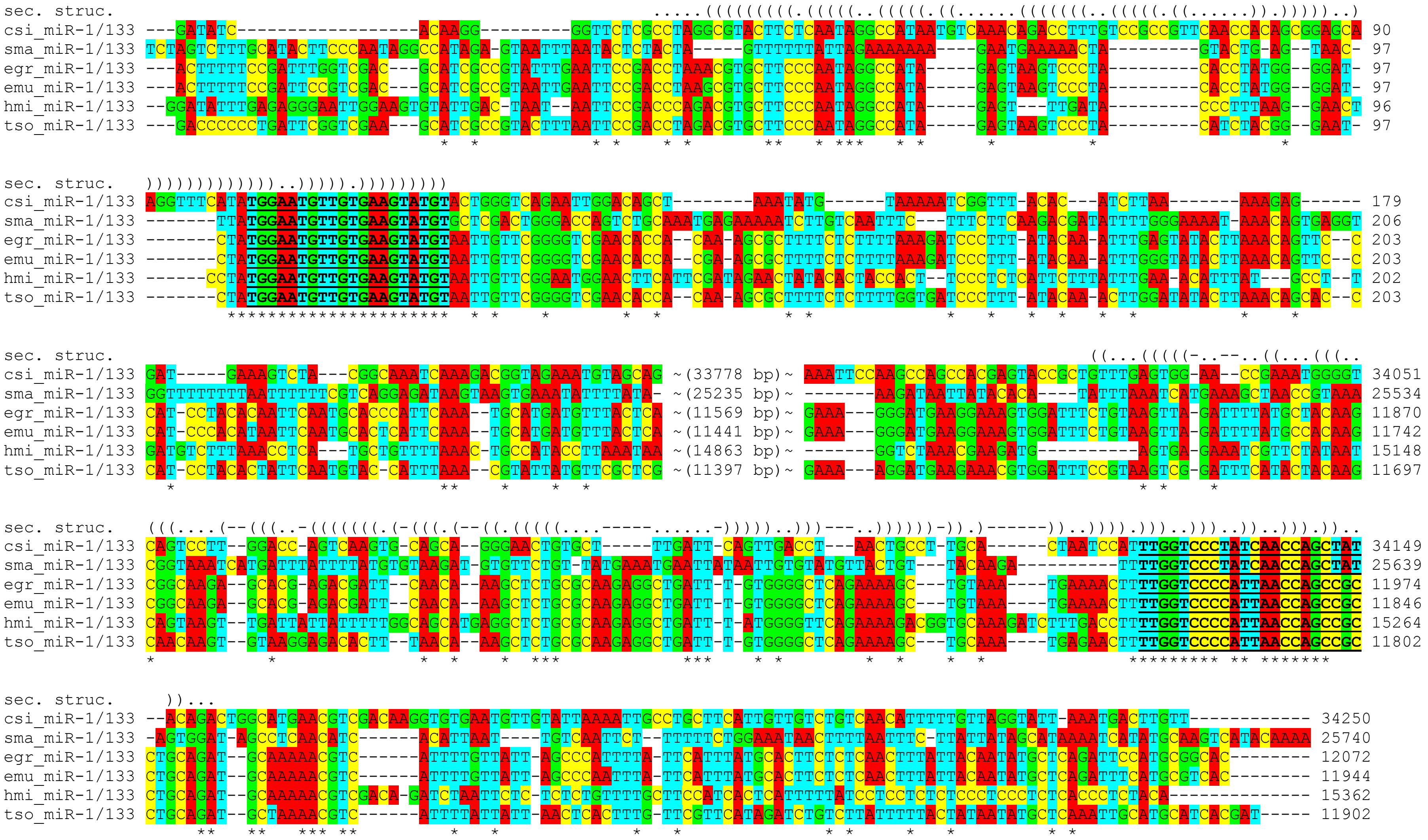
sec. struc.
csi_miR-1/133
sma_miR-1/133
egr_miR-1/133
emu_miR-1/133
hmi_miR-1/133
tso_miR-1/133
90
97
97
97
96
97
179
206
203
203
202
203
~(33778 bp)~
~(25235 bp)~
~(11569 bp)~
~(11441 bp)~
~(14863 bp)~
~(11397 bp)~
34051
25534
11870
11742
15148
11697
34149
25639
11974
11846
15264
11802
34250
25740
12072
11944
15362
11902
