# Supplementary material for: Identification of microRNA Genes in Three Opisthorchiids
Source: PLoS Negl Trop Dis. 2015 Apr 21;9(4):e0003680. doi: 10.1371/journal.pntd.0003680 (PMC4405270; doi:10.1371/journal.pntd.0003680)
Supplement: S6 Appendix — (PDF) [file pntd.0003680.s006.pdf]

S6 Appendix. Gene prediction in region between miR-1 and miR-133

Gene prediction in region between miR-1 and miR-133 in *C. sinensis* genome

Length of sequence: 34008
Number of predicted genes 1: in +chain 1, in -chain 0.
Number of predicted exons 1: in +chain 1, in -chain 0.
Positions of predicted genes and exons: Variant 1 from 1, Score:7.841427

G	Str	Feature	Start	End	Score	ORF	Len
1	+	TSS	33256		-2.00		
1	+	1 CDSo	33380 -	33631	9.10	33380 - 33631	252
1	+	PolA	33679		1.26		

Predicted protein(s):
>FGENESH:[mRNA] 1 1 exon (s) 33380 - 33631 252 bp, chain +
ATGCGTGCAGTTCACTTTCCAGCGAAACGTCTGAAACAAGATCAACGCAGAGAGATTCCG
GTACATGGAATTTTGCGATGCAGCTCAGAATCCCCATCTGAAACCGTATTATCCGGTGTA
GTTAGAAAACCGGGGACCGCGGGCAGAACATCCGGCGGTCGACTGAAGAAAGCGCCAGTT
GAACAAGGTGCAGATAAACGGTATTTGTTCAACTTACACAAGTATGCACAGGTATACTCA
ATAATATATTAG
>FGENESH: 1 1 exon (s) 33380 - 33631 83 aa, chain +
MRAVHFPAKRLKQDQRREIPVHGILRCSSESPSETVLSGVVRKPGTAGRTSGGRLKKAPV
EQGADKRYLFNLHKYAQVYSIIY

Gene prediction in region between miR-1 and miR-133 in *S. mansoni* genome

Length of sequence: 25498
Number of predicted genes 2: in +chain 1, in -chain 1.
Number of predicted exons 8: in +chain 2, in -chain 6.
Positions of predicted genes and exons: Variant 1 from 1, Score:37.246964

G	Str	Feature	Start	End	Score	ORF	Len

1	+		TSS	514		-1.92				
1	+	1	CDSf	1309 -	1354	3.36	1309 -	1353	45	
1	+	2	CDSl	6721 -	6740	-5.03	6723 -	6740	18	
1	+		PolA	6857		1.88				
2	-		PolA	7756		1.88				
2	-	1	CDSl	8114 -	8185	0.10	8114 -	8185	72	
2	-	2	CDSi	12428 -	12464	-4.20	12428 -	12463	36	
2	-	3	CDSi	17148 -	17205	-0.43	17150 -	17203	54	
2	-	4	CDSi	20437 -	20483	-6.57	20438 -	20482	45	
2	-	5	CDSi	23596 -	23619	3.37	23598 -	23618	21	
2	-	6	CDSf	24831 -	24847	-0.73	24833 -	24847	15	
2	-		TSS	25130		-4.81				

Predicted protein(s):
>FGENESH:[mRNA] 1 2 exon (s) 1309 - 6740 66 bp, chain +
ATGTTTACCATATTTGAAGATAATTCACAGCAGCCTAAGCAAACTATTGGTGGAGAAGAT
TTATAG
>FGENESH: 1 2 exon (s) 1309 - 6740 21 aa, chain +
MFTIFEDNSQQPKQTIGGEDL
>FGENESH:[mRNA] 2 6 exon (s) 8114 - 24847 255 bp, chain -
ATGCAAGTTATTGAACAAATATTATTATGCTTAGCCTACAATTTTCAAAATAGTTTAACA
AATTCATATTTTAGTTCATACTACTTTAAAACAAAACAAACTAATAAAAAGAATAAGTTT
ATTTTAAAAAAGTGTATTACTAACAAGTTTAATAATTTTAAACGTGTGCAAACTCAAGTG
CAGTGTGCATTACTTAAAGAGTTAAAAATAAATGAATTATCTAAAATCATGTTTAAATTC
ATAGTCAATAGTTGA
>FGENESH: 2 6 exon (s) 8114 - 24847 84 aa, chain -
MQVIEQILLCLAYNFQNSLTNSYFSSYYFKTKQTNKKNKFILKKCITNKFNNFKRVQTQV
QCALLKELKINELSKIMFKFIVNS

Gene prediction in region between miR-1 and miR-133 in *H. microstoma* genome

Length of sequence: 15123

Number of predicted genes 3: in +chain 1, in -chain 2.
Number of predicted exons 6: in +chain 2, in -chain 4.
Positions of predicted genes and exons: Variant 1 from 1, Score:21.828844

G	Str	Feature	Start	End	Score	ORF	Len
1	-	PolA	60		2.62		
1	-	1 CDSl	90 -	120	-8.41	90 - 119	30
1	-	2 CDSf	4016 -	4125	1.52	4018 - 4125	108
1	-	TSS	4290		1.49		
2	+	TSS	5110		-5.80		
2	+	1 CDSf	5271 -	5372	8.35	5271 - 5372	102
2	+	2 CDSl	7571 -	7735	-3.63	7571 - 7735	165
2	+	PolA	7739		2.62		
3	-	PolA	9165		0.42		
3	-	1 CDSl	9201 -	9314	-2.19	9201 - 9314	114
3	-	2 CDSf	12154 -	12159	2.27	12154 - 12159	6
3	-	TSS	13000		0.09		

Predicted protein(s):
>FGENESH:[mRNA] 1 2 exon (s) 90 - 4125 141 bp, chain -
ATGAGCATACTTATCTTCGCTCTTGTAAACCAACATCTACCTCTATTTCTATTCACTTGC
TCTAGAAATAAAGTTTCAGCAAAACGCTCTCAAAAATATCGTGAATTGAGGTATGGCAGT
TTAAAACAGCATGAGGTTTAA
>FGENESH: 1 2 exon (s) 90 - 4125 46 aa, chain -
MSILIFALVNQHLPLFLFTCSRNKVSAKRSQKYRELRYGSLKQHEV
>FGENESH:[mRNA] 2 2 exon (s) 5271 - 7735 267 bp, chain +
ATGGTTGGCCAAGCACATCACGGAATAGGCGTAGAATCCATAACCTCGGACTTAGCAATT
GCCTATTGGCAAGACTTATTAAACGAGGTATGGAGACAAACGCAATTTATGCGTTCCTGC
GATTTGCTGCCTACATTTTGCCAAATTGAACCGCTTCCCAATCTACATCCCCCTCTTACA
CTTGCAGGGAAAGCAAATACCGCTCCGCGAAAATGTCTAATTAAAAGACGATTAAGACAA
ACTACTGATAAACTTAATCCCATTTGA

>FGENESH: 2 2 exon (s) 5271 - 7735 88 aa, chain +
MVGQAHHGIGVESITSDLAIAYWQDLLNEVWRQTQFMRSCDLLPTFCQIEPLPNLHPPLT
LAGKANTAPRKCLIKRRLRQTTDKLNPI
>FGENESH:[mRNA] 3 2 exon (s) 9201 - 12159 120 bp, chain -
ATGGAGGGTATAATTTTGAGGAAAAATGCACACATATTGAATTGTGCAGGTGTAAAGTTA
ATTGAGGCGATTGCAAATTGTATAGTTCATGAAGTGTTTCCTTATTCAAAGCACCCCTGA
>FGENESH: 3 2 exon (s) 9201 - 12159 39 aa, chain -
MEGIILRKNAHILNCAGVKLIEAIANCIVHEVFPYSKHP

Gene prediction in region between miR-1 and miR-133 in *E. granulosus* genome

Length of sequence: 11833
Number of predicted genes 1: in +chain 1, in -chain 0.
Number of predicted exons 4: in +chain 4, in -chain 0.
Positions of predicted genes and exons: Variant 1 from 1, Score:1.270266

G	Str	Feature	Start	End	Score	ORF	Len
1	+	1 CDSf	820 -	973	4.61	820 - 972	153
1	+	2 CDSi	4041 -	4197	3.52	4043 - 4195	153
1	+	3 CDSi	7100 -	7259	0.01	7101 - 7259	159
1	+	4 CDSi	11650 -	11727	4.14	11650 - 11727	78

Predicted protein(s):
>FGENESH:[mRNA] 1 4 exon (s) 820 - 11727 549 bp, chain +
ATGGGAGCGGACGGGGAAGAACTTAACAGTGCAATAGCTCCTTCAATGCACGTTCTCATG
CACGATCAAGCACTGAACGGCGACAGTGTAACTACAGCCTTTGCAACCCATTCGCAGCGA
AGAGGGGAGCGAGATGAAAGTGAAGTGGGAGGAGGGTTAAAGACTGCGAGCGTAGCTTTG
GAGAGGGCGAAACCTCAAACGTGGATTAATGGAAGCTCCTCAAAGCGTAGCATAGCTAGA
ATAAAGTCAGAATTTAATGGAACGATAAAAAATGGAAATAAACACCTGCTTATTAACCAA
AATCTTCCACATATTACTACAACCAACGGCGTTCTATCTCGAATAGTTGGATACGCTGGT
GAAAGACACCGCTATGATCTCCTTTCCCAATATTTTTTGTCTAAGAGGTACATTCATTTG
CACACTAGTGAGGACCGTATGCTATTTTTACTGTTGGCTTTTTACCAAAAGGGGTCAATT
CGTAATGCACCGTGGAAGAGATCTTCTATCTGTACGATGGAGGAAAGGGATGAAGGAAAG

TGGATTTCT
>FGENESH: 1 4 exon (s) 820 - 11727 183 aa, chain +
MGADGEELNSAIAPSMHVLMHDQALNGDSVTTAFATHSQRRGERDESEVGGGLKTASVAL
ERAKPQTWINGSSSKRSIARIKSEFNGTIKNGNKHLLINQNLPHITTTNGVLSRIVGYAG
ERHRYDLLSQYFLSKRYIHLHTSEDRMLFLLLAFYQKGSIRNAPWKRSSICTMEERDEGK
WIS

Gene prediction in region between miR-1 and miR-133 in *E. multilocularis* genome

Length of sequence: 11705
no reliable predictions

Gene prediction in region between miR-1 and miR-133 in *T. solium* genome

Length of sequence: 11660
Number of predicted genes 2: in +chain 2, in -chain 0.
Number of predicted exons 2: in +chain 2, in -chain 0.
Positions of predicted genes and exons: Variant 1 from 1, Score:8.071529

G	Str	Feature	Start	End	Score	ORF		Len
1	+	TSS	1497		-4.80			
1	+	1 CDSo	1546 -	1815	8.39	1546 -	1815	270
1	+	PolA	2062		1.26			
2	+	TSS	6789		-0.90			
2	+	1 CDSo	6948 -	7271	6.54	6948 -	7271	324
2	+	PolA	8092		1.26			

Predicted protein(s):
>FGENESH:[mRNA] 1 1 exon (s) 1546 - 1815 270 bp, chain +
ATGAGTACCCAAATTATCAAGGGAATAAGGCGTTTGACTAGACACACGCAAACAGTGAGG
AGGAATTGGGGTCAGATGGCTGCGCTACATAACGCTACATCTCCGTTTTGCGGAAAACTC
GTCGATGGTGATCCGAAAAGAAGTTACAAAATGAAAGAAAACTTCGGCTGGGCTTCAACT
AGAACAAAATCCACTGAGGCATACAGAGTAGAGAGAAATAAAAAGGGTGAACCGGTCGTC

TGTACAGCGTGTGTAATCAACGTCGTGTGA
>FGENESH: 1 1 exon (s) 1546 - 1815 89 aa, chain +
MSTQIIKGIRRLTRHTQTVRRNWGQMAALHNATSPFCGKLVDGDPKRSYKMKENFGWAST
RTKSTEAYRVERNKKGEPVVCTACVINVV
>FGENESH:[mRNA] 2 1 exon (s) 6948 - 7271 324 bp, chain +
ATGAAGCTTATTACAGAGCTTAAAGTGCCTCCGAGCAGGATGGGATTAACATCCACGAGT
AGCGTTAATAAGGCCTTTGACTTAATGAGCTGTCACATTTCACATCGACTCGCAGGTGGC
ATCACGTTTATTTGCTGGGGCAATACGTACATTGTCAAAATGCTTCATTTATTGGAAGTG
TTACCACAACAAGAGGCATTCCATCTCGATCAATCGGGTACGCTGGTGAATTGCAACGAT
GCGATCCCATTTCGCAATACCTTCTGTCTAAGGAGTACATTTATTTGCACACAAATGAGA
ACCGTATGCTATTTTTACGATTAG
>FGENESH: 2 1 exon (s) 6948 - 7271 107 aa, chain +
MKLITELKVPPSRMGLTSTSSVNKAFDLMSCHISHRLAGGITFICWGNTYIVKMLHLLEV
LPQQEAFHLDQSGTLVNCNDAIPFRNTFCLRSTFICTQMRTVCYFYD
