# Supplementary material for: Identification of microRNA Genes in Three Opisthorchiids
Source: PLoS Negl Trop Dis. 2015 Apr 21;9(4):e0003680. doi: 10.1371/journal.pntd.0003680 (PMC4405270; doi:10.1371/journal.pntd.0003680)
Supplement: S7 Appendix — (PDF) [file pntd.0003680.s007.pdf]

S7 Appendix. Exons surrounding intronic miRNA, and part of protein encoded by them.

C. sinensis

32nd exon

TTGGCCCTGGTTCTACAAACCGTGGTTTGTACGTCCGGCCCATCTGCTACCCGTTTCGACCACAGAAAGCGATTCCGGAGCAGGACGAG
GATAGGAGTGGCGACGAGGCTGAATTCCGTATTGCAACGGCTATCGACCAGTTAAAGTCTATACTGGATTGCGAG

33rd exon

CCACCCAGTGACCCTACGATCCTTCAGTCTGCGCACTCGGTGGCTGTTGCAACCCAAAGCCTGATTCACACAGCTAGGTCTATTGGATT
GGCCCCACCGTCGGATGCAGTGGACCGTGCTCGGCGTGCTGGCACTTCTCACCATGGTGCCTCGCTATGGCGAACCGAACTCACTGTGG
CCTTGGCAACACAGGATATGTGCCAGCTAGCCCAACTGTGTTCAGCCGTTCTTCAGGAGGAGAATGCACATCCTGACTCGAAAGATCT
ATTTGGCACCTCCACACGTCTTGTTCCGTCCAAGGAGCGTCTGTTGGCCGCTGTACGTCGGGTTGCCGCTGCTTCTGCTCAGCTCCTGAT
GTCCGCCAAATCCAGAAGGTCCAAATGTTCTTCCGCGGATATTCAAAAGTTGCAG

>csi

VGPGSTNRGLYVRPICYPFRPQKAIPEQDEDRSGDEAEFRIATAIDQLKSILDCEPPSDPTILQSAHSVAVATQSLIHTARSIGLAPPSDAVDRAR
RAGTSHHGASLWRTELTVALATQDMCQLAQLCSAVLQEENAHPDSKDLFGTSTRLVPSKERLLAAVRRVAAASAQLLMSAKSRRSKCSSA
DIQKLQ

S. japonicum

40th exon

GTGGTGGTAGTGGCTCAGGATCATCACATAAAAGTATTTTTATCCGCCCTACATGTTATCCTCTTCAAGTGTCACATGATCAAGGTGGT
GAACAAATTATTAACGATGAAGCTGAATGTCGGATTGTGGATGCTATCAATCAACTTAATTTATCGCTGGAAAAATGC

41st exon

TTAGTAGATAATAAATTAGCTCAATCTCTTCTTGTTTCGGCTCGATCAGTGGCTGTGTCTGTTCAAAATCTCATCAGAACCGCAAGGACT
GTTGTTTCCGTACCACCAAACGATGCGGTGGAGAGAGCTCGGACAGCAGAAAGTGCTTCGCAAAAGAGTACTAGTTCGGCTGTTCTAT
GGCGTACTGAACTCAATGTATCTCTTGCCACCCAACACTTATGTCAGTTGAGCAAACTGTGTTCCGAAACAGTACCGTCCTCAACCTCC
ACGAAACTTGAGAACAACACTGGGGAGCTATCAGGAGGATTGATTCTGTCGCGTGAAAGGCTTCTGGCTGCTGTGCGTCAAGTGGCGA
CTGCGGGAGCCCAGTTGCTCTTAGCAGCCAAATTGAGGAAAGAAGCTCTAATGTCTTCTGATATCCGGAAATTAAAAG

>sja

GGSGSGSSHKSIFIRPTCYPLQVSHDQGGEQIINDEAECRIVDAINQLNLSLEKCLVDNKLAQSLLVSARSVAVSVQNLIRTARTVVSVPPNDA
VERARTAESASQKSTSSAVLWRTELNVSLATQHLCQLSKLCSETVPSSTSTKLENNTGELSGGLILSRERLLAAVRQVATAGAQLLLAAKLRK
EALMSSDIRKLK

S. mansoni

7th exon

GTGGCAGTTCAAAATCACACAAAAGTATTTTCATTCGTCCCACATGTTACCCTCTTCAAATATCGTCTGAGCAAACCACTGAGCAAACT
ATGAACGATGAAGCAGAATGTCGAATTGTCGATGCTATTAATCAGCTTAATTCATCGTTGGAAAAATGC

8th exon

TCGGCGGACAATAAATTAGCTCAATCTCTTCTTGTCACCGCGCGATCAGTCGCTGTATCTGTTCAAAATCTTATCAGAACTGCAAGGAC
TGTTATTTCCGTACCCCCAAGCAATGCTGTCGACAGAGCTCGGACGGCAGAAGGTGCTTCACAAAAGAGTACTAGCTCAGCTGCTCTTT
GGCGTACTGAACTTAATGTATCGCTTGCTACTCAACACTTGTGTCAACTGTCTAAATTATGTTCGGAAACAGTACCATCCATAACCTCG
CAAAAATTGGAGAATAACGCCGGGGAGTTATCAGGAGGATTAATTTTATCACGCGAAAGACTTCTGGCTGCTGTGCGTCAAGTGGCAA
CTGCAGGAGCTCAGTTACTCTTAGCAGCTAAATTAAGAAAAGAAGCTTTAATGTCTGCTGATATTCGAAAGTTGAAA

>sma

GSSKSHKSIFIRPTCYPLQISSEQTTEQTMNDEAECRIVDAINQLNSSLEKCSADNKLAQSLLVTARSVAVSVQNLIRTARTVISVPPSNAVDRA
RTAEGASQKSTSSAALWRTELNVSLATQHLCQLSKLCSETVPSITSQKLENNAGELSGGLILSRERLLAAVRQVATAGAQLLLAAKLRKEAL
MSADIRKLK

E. granulosus

44th exon

ATGGCTCCAACAGAGCCACAAGTAGCCAGATCAGTTGTCTGCAATCCCAGCTCGATTCAACAGGTCGTCTGTTGACTGGAACTGAGGC
TGCGCTAGAAGCCAGTAGACGAATTGATGTTGCTTTAGGCCTACTGGAAGGTGATTTTTATCAG

45th exon

AGCATGCAGAGGTCGACCAATCAGGATGTTGTACATCACACCCAACCAATCCTGAGTATGGCTCGTGCAATTACCCTAGCAACACGGG
AATTCGTACAGACTGTCCAATTAGCTTCCACCTCCAAAGCCACGGCTAATTTTGGTCGACTGG

>egr

GSNRATSSQISCLQSQLDSTGRLLTGTEAALEASRRIDVALGLLEGDFYQSMQRSTNQDVVHHTQPILSMARAITLATREFVQTVQLASTSKA
TANFGRL

E. multilocularis

39th exon

ATGGCTCCTACAGAGCCACGAGTAGCCAGATCAGTTGTCTGCAATCCCAGCTCGATTCAACAGGTCGTCTGTTGACTGGAAATGAGGCT
GCGCTAGAAGCCAGTAGACGAATTGATGTTGCTTTAGGCCTACTGGAAGGTGATTTTTATCAG

40th exon

AGCATGCAGAGGACGACCAATCAGGATGTTGTACATCACACCCAACCAATCCTGAGTATGGCTCGTGCAGTTACCCTAGCAACACGGG
AATTCGTACAGACTGTCCAATTAGCTTCCACCTCCAAAGCCGCGGCTAATTTTGGTCGACTG

>emu

GSYRATSSQISCLQSQLDSTGRLLTGNEAALEASRRIDVALGLLEGDFYQSMQRTTNQDVVHHTQPILSMARAVTLATREFVQTVQLASTSK
AAANFGRL

H. microstoma

37th exon

ATGGATTTAGCAAGTCTATTAGTATTCGAACTAGCTGCCTTCAATCTCAATTAGACACAAAGGTTTTTCTTATGAGCGGTGCAGAAGCT
GCCATTGAAGCAATTCACCGGATTGATATCGCTCTGGGTCTGCTGGAATCGGATCTCAACAAG

ex38th exon

AGCAAGCAAGAACCGATCAATCAGGATATTGTGGTTCATACGCAACCTATATTGAGCTTAGCTCGATCAACTACTTTAGCTATACGAGA
ATTTGTTCAAACAGTCCAATTGGCGTTGGCCTCTCGAGAATCGACTAATCTCGGCCGATTA

>hmi

GFSKSISIRTSCLQSQLDTKVFLMSGAEAAIEAIHRIDIALGLLESDLNKSKQEPINQDIVVHTQPILSLARSTTLAIREFVQTVQLALASRESTNL
GRL

T. solium

40th exon

ATGGCTCCTACAGGGCTACAACCAGTCAGCTCAGTTGTCTACAATCCCAGCTCGATTCAACAGGTCGCCTGCTAACTGGGACCGAGGCT
GCATTGGAAGCCAGTAGACGAATCGATGCTGCCCTAGGCTTACTGGAGGGCGATCTCTATCAG

41st exon

AGTATGCAAAAATCAATCAATCAGGATGTCGCACATCACACACAACCAATCCTAAGCATGGCCCGTGCAGTTGCCCTAGCAACACGGG
AATTCGTGCAGACTGTCCAATTAGCTTTCACCTCCAAGACCTCTGCAAATTTGGGTCGATTGG

>tso

GSYRATTSQLSCLQSQLDSTGRLLTGTEAALEASRRIDAALGLLEGDLYQSMQKSINQDVAHHTQPILSMARAVALATREFVQTVQLAFTSK
TSANLGRL

```
sja GGSGSGSSHKSIFIRPTCYPL--QVSHDQGGEQIINDEAECRIVDAINQLNLSLE
sma ---GSSKSHKSIFIRPTCYPL--QISSEQTTEQTMNDEAECRIVDAINQLNSSLE
csi --VGPGSTNRGLYVRPICYPFRPQKAIPEQDEDRSGDEAEFRIATAIDQLKSILD
       *...:::.:::** ***:  * :  :  *:  .**** **. **:**:  *:

sja KCLVDNKLAQSLLVSARSVAVSVQNLIRTARTVVSVPPNDAVERARTAESASQKS
sma KCSADNKLAQSLLVTARSVAVSVQNLIRTARTVISVPPSNAVDRARTAEGASQKS
csi ---CEPPSDPTILQSAHSVAVATQSLIHTARSIGLAPPSDAVDRARRAG----TS
        :     ::* :*:****:.*.**:***::  .**.:**:*** *     .*

sja TSSAVLWRTELNVSLATQHLCQLSKLCSETVPSSTSTKLENNTGELSGGLILSRE
sma TSSAALWRTELNVSLATQHLCQLSKLCSETVPSITSQKLENNAGELSGGLILSRE
csi HHGASLWRTELTVALATQDMCQLAQLCSAVLQEENAHPDSKDLFGTSTRLVPSKE
      .* ******.*:****.:***::*** .: . .:   .::    *  *: *:*

sja RLLAAVRQVATAGAQLLLAAKLRKEALMSSDIRKLK
sma RLLAAVRQVATAGAQLLLAAKLRKEALMSADIRKLK
csi RLLAAVRRVAAASAQLLMSAKSRRSKCSSADIQKLQ
    *******:**:*.****::** *:.   *:**:**:
```

```
egr GSNRATSSQISCLQSQLDSTGRLLTGTEAALEASRRIDVALGLLEGDFYQSMQRS
emu GSYRATSSQISCLQSQLDSTGRLLTGNEAALEASRRIDVALGLLEGDFYQSMQRT
tso GSYRATTSQLSCLQSQLDSTGRLLTGTEAALEASRRIDAALGLLEGDLYQSMQKS
hmi GFSKSISIRTSCLQSQLDTKVFLMSGAEAAIEAIHRIDIALGLLESDLNKSKQEP
    *  :: : : ********:.  *::* ***:** :*** ******.*: :* *..

egr TNQDVVHHTQPILSMARAITLATREFVQTVQLASTSKATANFGRL
emu TNQDVVHHTQPILSMARAVTLATREFVQTVQLASTSKAAANFGRL
tso INQDVAHHTQPILSMARAVALATREFVQTVQLAFTSKTSANLGRL
hmi INQDIVVHTQPILSLARSTTLAIREFVQTVQLALASRESTNLGRL
     ***:. *******:**: :** ********** :*: ::*:***
```

```
csi --VGPGSTNRGLYVRPICYPFRPQKAIPEQDEDRSGDEAEFRIATAIDQLK-SIL
sja GGSGSGSSHKSIFIRPTCYPL--QVSHDQGGEQIINDEAECRIVDAINQLN-LSL
sma ---GSSKSHKSIFIRPTCYPL--QISSEQTTEQTMNDEAECRIVDAINQLN-SSL
egr ---G---SNRATSSQISCL----QSQLDSTGRLLTGTEAALEASRRIDVAL-GLL
emu ---G---SYRATSSQISCL----QSQLDSTGRLLTGNEAALEASRRIDVAL-GLL
hmi ---G---FSKSISIRTSCL----QSQLDTKVFLMSGAEAAIEAIHRIDIAL-GLL
tso ---G---SYRATTSQLSCL----QSQLDSTGRLLTGTEAALEASRRIDAAL-GLL
       *     :.   :  *     *           . **  .    *:      *

csi ---D--------------C--EPPSD--PTILQSA------HSVAVATQSLIHTARSIGLAPP
sja E--K--------------CLVDNKLA--QSLLVSA------RSVAVSVQNLIRTARTVVSVPP
sma --EKC--------------SADNKLA--QSLLVTA------RSVAVSVQNLIRTARTVISVPP
egr --E-G-----D--------FYQSMQ---RSTNQ---------DVVHHTQPILSMARAITLA-T
emu --E-G-----D--------FYQSMQ---RTTNQ---------DVVHHTQPILSMARAVTLA-T
hmi E-SDL--N-------------KSKQE---PI----N-----QDIVVHTQPILSLARSTTLA-I
tso EGD-L---------------YQSMQ---KSINQ---------DVAHHTQPILSMARAVALA-T
                         .       .            .:.  .* ::  **:   .

csi SDAVDRARRA-GT----SHH------GASLWRTELTVALATQDMCQLAQLCSAVLQEENAHPD
sja NDAVERARTA-ES---ASQKSTS---SAVLWRTELNVSLATQHLCQLSKLCSETVPSSTSTKL
sma SNAVDRARTAEGA----SQKSTS---SAALWRTELNVSLATQHLCQLSKLCSETVPSITSQKL
egr REFVQTVQLA-ST----SKA--------------------TANFGRL----------------
emu REFVQTVQLA-ST----SKA--------------------AANFGRL----------------
hmi REFVQTVQLA-LA----SRE--------------------STNLGRL----------------
tso REFVQTVQLA-FT----SKT--------------------SANLGRL----------------
     : *: .: *  :    *:                     : .: :*

csi SKDLFGTSTRLVPSKERLLAAVRRVAAASAQLLMSAKSRRSKCSSADIQKLQ
sja ENNTGELSGGLILSRERLLAAVRQVATAGAQLLLAAKLRKEALMSSDIRKLK
sma ENNAGELSGGLILSRERLLAAVRQVATAGAQLLLAAKLRKEALMSADIRKLK
egr ----------------------------------------------------
emu ----------------------------------------------------
hmi ----------------------------------------------------
tso ----------------------------------------------------
```
