# Supplementary material for: Identification of microRNA Genes in Three Opisthorchiids
Source: PLoS Negl Trop Dis. 2015 Apr 21;9(4):e0003680. doi: 10.1371/journal.pntd.0003680 (PMC4405270; doi:10.1371/journal.pntd.0003680)
Supplement: S8 Appendix — (PDF) [file pntd.0003680.s008.pdf]

S9 Appendix. Flatworms talin alignment.

Species	Accession number (gene)	Protein name	Database
Clonorchis sinensis	CLF_105939	talin	UniProt
Echinococcus granulosus	EgrG_000736000	talin	UniProt
Echinococcus multilocularis	EmuJ_000736000	talin	UniProt
Hymenolepis microstoma	HmN_000220000	apolipoprotein A I binding protein (similar to talin)	UniProt
Schistosoma japonicum	Sjp_0006570	talin	GeneDB
Schistosoma mansoni	Smp_037860	putative talin	UniProt
Taenia solium	TsM_000902500	talin	GeneDB

C. sinensis vs *S. japonicum*

```
Score   Expect  Method  Identities     Positives      Gaps
2767 bits(7172)      0.0    Compositional matrix adjust.  1513/2497(61%) 1848/2497(74%) 120/2497(4%)
Query  20    NLGGVGATIVQHQRLPPPRRVGPKDGFVNGALEETI-QPKMDDSIQMATGY-MQTNG-WS-HQ--NLHTD-RTDFVDGQ----WAERPMSPDNFMMGGSHTFHYQEITPAQRALLVTIIE  128
             N+    ATIVQHQR+P   R   KD  VNG +E+   Q    DS    +G+ + TNG WS HQ  NL  D  +  +DGQ       R MSPDNFM GG+H FHYQE++PAQ+ LLVTI E
Sbjct  436   NVQAERATIVQHQRVPVRGR--GKDQLVNGVVEKPFDQYHKTDS----SGHQIHTNGVWSIHQQNNLIGDENSTVIDGQSMLTTTTRQMSPDNFMTGGTHIFHYQEMSPAQKFLLVTINE  549

Query  129   DVETLQAAKEQLYLSPDEDR-IIGIGSDEASKRWLSESIGASQAKVTDEVGAMNAAVAQALRSANRAEAYDSTGMSQVG--LGVSQDPGDDLMMMQQSFRVVTIHFPAFVDDVKRVAVLR  245
             D+ETLQAAK+ L + P  +R ++ +G+DEASKRWLSES+GASQAKVTDEVGAMNAAVAQALRSANRAE+ D   + Q G  L  S+DPGDDLMMMQQSFRVVTIHFPAFVDD+KRVAVLR
Sbjct  550   DLETLQAAKDNLDIGPPTERHLLNVGTDEASKRWLSESMGASQAKVTDEVGAMNAAVAQALRSANRAESAD-INVVQHGQSLDQSRDPGDDLMMMQQSFRVVTIHFPAFVDDLKRVAVLR  668

Query  246   REVGAIRADQTGEPVIAAQSEESSQNLLSAARHVADAFTDLLESARPLATGQPREEV--VTDESTIRSQAVTT------GPVSSSSRKAIIDAASRVGEASNDLLRHVMHEGDGEEDFEA  357
             RE G ++ADQTG+P+IA ++EES+QNLLSAARHVADAFTDLL+SARPLA GQ  EE+   +D+    ++  VTT       GPVSSSSRKAII+AASRVG ASNDLLRHVMH GD ++ F
Sbjct  669   RESGVLKADQTGQPIIAEKTEESTQNLLSAARHVADAFTDLLQSARPLAMGQTHEEIYPTSDDGNYQANRVTTTQQAQQGPVSSSSRKAIIEAASRVGAASNDLLRHVMHGGDDDDLFNT  788

Query  358   SSLMLTEEERLYKDQLLSLAKAVANTTASLVVKAKNLATQTSLDPEAQQHVIAAATQTGLCTSQLVACTKVLAPTIYQPSCQQQLSEAAREVSWAVDGVVQASRAAGARTADQPVQVQQP  477
             + L+LT+EER+Y+DQLL LAKAVAN TA LVVKAK LA+Q + DPEAQQ VIA+ TQTGLCTSQLVACTKVLAPTI+QP+CQQQLSEA REV+WAVDGVVQASRAA     +D P  VQQ
Sbjct  789   NYLILTDEERIYQDQLLGLAKAVANATAGLVVKAKVLASQITSDPEAQQRVIASVTQTGLCTSQLVACTKVLAPTIHQPTCQQQLSEAGREVTWAVDGVVQASRAAIHVPSDDPQMVQQ-  907

Query  478   VRTAVAETEMAATEVRDALDQLNAHLSKASAKTSSGDALDNFQLAYDQLQQYHTHDGQRMVASARRMAQATAQMIADIKAQAELAADDPDRQSRLFAAAKQLADATTILIASAKVCSTNP  597
                ++ ETE AATEVRDALDQLNAHL K S K    DAL+NFQ AY+ L+QY+T DGQRMVASARR+AQATA MIADIKAQAE + DD DRQ+RLF AAKQLADATT LI +AK CS+NP
Sbjct  908   ---SIIETETAATEVRDALDQLNAHLLKGSYKGYQSDALNNFQTAYEDLKQYYTTDGQRMVASARRLAQATALMIADIKAQAEESGDDSDRQNRLFLAAKQLADATTNLINAAKSCSSNP  1024

Query  598   ENPVTQEELRQAAESLNLIVHSAAADLLHRRLIRNLQAAARAAVTGATQLVNVSQVAAKRSRGNTYQVISDGKLVNGLIPRTVISIRESRACPDDPLTQVELISACERFVLPCESLVRSS  717
             +N    Q ELR AA+ LN + +SA   +    RLI NLQ+AARA VTGATQLVN+SQ A K SRGN+YQ+ +D K V  LIP+TVI+IRESRA PDDP+T +ELI+ACERFV PC ++VRSS
Sbjct  1025  DNIRLQTELRDAADELNTLAYSAGGEQFQTRLIHNLQSAARATVTGATQLVNISQAAIKYSRGNSYQLQNDTKNVTNLIPKTVITIRESRANPDDPMTLLELINACERFVQPCTAMVRSS  1144

Query  718   RSVAPTVSDPTTQAALDNSTSQLSGAIEVLRTCLARLTPLTRQMQVDGALARLARLAVEAAAIETGLKQGTLAPLPDEKIDDCFNLLGTSVQDANVSTHELHSIVETMAQQQRDMMSRSD  837
             +S+APT+SDP+ QAAL+NS SQL+ A+E L  CL +   PL R  Q+DGAL RLARL+ E AA+ET L QGTLA +P++KI+DC +LL  SV+D    S    L SI+ TMA+     +++ +
Sbjct  1145  KSLAPTLSDPSIQAALENSNSQLASALETLIACLIKAAPLARHFQLDGALTRLARLSDEVAAMETSLIQGTLASIPEQKIEDCCHLLNVSVRDGTESAKNLDSIISTMAKMDSTSLNQLE  1264
```

```
Query  838   VCGVPASMLATAIAGLVHATRGIVAHDAYDAADAGRSGAVGPSSDSTRLAVTQDTRQAVQLAYELVKAARDARFAYDDGQMEALATVVSHSEQLTVQLLDTLGRCLRGLPGHREINEATN  957
             + G  A  LA+A+ GLVHATRGI+A     +   G SG    +++STR AV +DTRQAVQLAYELV+AA+D R A D  Q    ATV SHS+QLT QL++TL RCLR LPGHREI+EAT+
Sbjct  1265  LAGQSADHLASAMTGLVHATRGIIADQNSLSYIGGSSGINTTAAESTRRAVIRDTRQAVQLAYELVRAAKDTRIACDAKQPANAATVQSHSQQLTAQLIETLTRCLRSLPGHREISEATH  1384

Query  958   LIEKRREDLVQLSHEAPTR-RVRWVEPSVYERAQSELTRAAVEFNQATGDLLSSYSPGAFRRTTRRFTGAYDTLTVKGVDLCM-SGVPPNEVPLPEPPPINRDLVDGLVNVSNHSYALMT  1075
             LIE+RR  L+Q S    T    R+V+P  Y+R Q++LT+AAVEFNQATGDLLSSYSPG FRRTTRRFTGAYD LT KG++L M S    N       P +N DL++GL+NVS+HSY LM
Sbjct  1385  LIEQRRLQLIQYSQPNQTSLSARFVQPDEYQRKQTDLTQAAVEFNQATGDLLSSYSPGTFRRTTRRFTGAYDHLTDKGIELSMCSEFSSNNQ--QSGPAVNSDLLNGLINVSDHSYVLMN  1502

Query  1076  EAGRVCARPDEQEIRDRFQTAARDVTESISQLLTICTSGVTAEQRDCEVALRRLEALRPLLENPNRPVNQKTYCECVDEVARSITPLADSLRTMSNAAKEKHTQEFGAAVRQCSNSMCQL  1195
             EA RVC +P+ + +R +FQ+AARDVTESISQLLT+CTSGV+ +QR+CE A+RRL++LRPLL NPNRP+NQ +Y +CVD VA+ + PLA+SLR+MS +A+E+   EF +AVRQCS+S+CQL
Sbjct  1503  EASRVCGQPEGENLRGKFQSAARDVTESISQLLTVCTSGVSPDQRECEAAIRRLDSLRPLLLNPNRPLNQHSYYDCVDTVAKCLEPLAESLRSMSTSAREQKPNEFSSAVRQCSDSVCQL  1622

Query  1196  IEETAQAAYLIGLADP----------------RSEPGRPSLVDPQLFMRSQREIQQVCDAICDPSITNRQIITLSTEMARSAKTLCEACSAVSAQTNNPDARHQLNALTRETMQSITALI  1299
             +EETAQ AYLIGLAD                  S PG  S++D  LF++SQ+EIQ  CDAIC+P++ NRQ+I+LSTEMARSAK LCEACS +S+ T N DAR +LN LT++TMQSIT+LI
Sbjct  1623  VEETAQTAYLIGLADRFENRRVSGATSMSSNISSPPGGSSMIDLDLFIQSQKEIQSACDAICEPNVNNRQVISLSTEMARSAKILCEACSQLSSHTTNIDARQRLNNLTKQTMQSITSLI  1742

Query  1300  QRRGNVSTAAAGSQLAEVNGEWDEANRQVTLANARGVSSNVARLVHMVTQGPQF----IGQAARVTDEAREAQQPVCSAGLSSLNAAQAVLRAAQTLITNARLGQPEQAFLSFSTASKEL  1415
             Q RG  ST A      +VNGE+ E+NRQ TL NA+ VS+NVA LV ++T  PQF    I Q  RVT   +E Q+PVC AGL SLNA QAVLR AQ L++ +R+G  E +F++FS+ASKEL
Sbjct  1743  QHRG--STVA-----TDVNGEFSESNRQQTLINAQTVSANVANLVKLITTEPQFTDLTISQ-ERVTSTIKETQRPVCVAGLESLNATQAVLRTAQNLLSTSRMGPSENSFMAFSSASKEL  1854

Query  1416  SESIKALAAAMREHAPGQLECQRVLQNINGLLQELQRAKMASMEGRLQPRRELNEEGFQKQLATCCRALLDAAPNVGRAARSEAEQLGHSVRVVDSYLPGITSSAIAAASRSPLSSTQLV  1535
             S S               Q ECQ V  NI  LL ELQ A++ASME RL PRRELNEEGFQ+QLAT  RALLD+AP+VGRAA SEAEQLG ++   DSYLPG+ S +IAAASRSP+ STQLV
Sbjct  1855  SNS--------------QRECQHVFNNIKKLLHELQDARIASMENRLPPRRELNEEGFQRQLATSVRALLDSAPSVGRAASSEAEQLGRTILTWDSYLPGLVSGSIAAASRSPMPSTQLV  1960

Query  1536  YLEHASTILEAADQLVRVARDAGGNPRATHLHPHLDEAVRGLMESCEDLLTALDDVASRQGHVSTLIDTINRSLAQTEEIIQVPVDARFADYQARLLRIARHMEQLTQAIQLRARQPPSD  1655
             YL+HA T+LEAA QL+ VA+D+GGNPRA HL   L+E+VRGL++SCE+ LT LDDVASRQGHVSTLIDT+NR+ AQ EEIIQVP+DARF+DYQ R+LRIARHM+QL QAIQLRARQP SD
Sbjct  1961  YLDHACTVLEAAGQLITVAKDSGGNPRAIHLQDSLNESVRGLVDSCEEFLTILDDVASRQGHVSTLIDTLNRAQAQAEEIIQVPMDARFSDYQTRMLRIARHMDQLNQAIQLRARQPASD  2080

Query  1656  GELAPLAHTLAQEYQEMCQTCKGAAATLPDARQADELRGAVRAVGMATAELVQATTAARIRAYDS-HGDQQMLLSRNQGIAYLDTRAEALDSKLRDLIALLDTQGPNTQACLQSASTVSG  1774
             GE+APLAH L QEYQEMCQTCKGAAATLPD  QAD+LR AVR VG A   L+QATT +R   Y S  G Q +LLSRN+ IA LD RAE LDSKLR+LI LL+T GP TQACLQSASTVSG
Sbjct  2081  GEIAPLAHNLTQEYQEMCQTCKGAAATLPDVNQADQLRSAVRGVGTACISLIQATTTSRSDVYGSTTGGQNLLLSRNKNIAELDRRAEFLDSKLRNLINLLETHGPGTQACLQSASTVSG  2200

Query  1775  IIADLDTTILFASSGTLHGPVHDDVDALLGPDDYRPPYALPGAGEAIADRG--------SRFGNREYPE----EGFGTVRDSIVRTARALVDDTQSLVSGTGEDQTRLATTAHVAVERVT  1882
             II+DLDTTILFASSGTLH PV DD DAL   +++         +   +A  G         SR G+R  PE    E F  +R+SI RTARALVDDTQSLVSGTGEDQ RLA+TAH AVER+T
Sbjct  2201  IISDLDTTILFASSGTLHPPVRDDFDALWADNEFGHSRLTDRSDHYLALSGNTDGTGIPSRAGSR--PEMHNAENFTPLRESIARTARALVDDTQSLVSGTGEDQARLASTAHTAVERIT  2318

Query  1883  QLADVVKRGAAVIGPGQPDTQVEVLSACRDVATGLRDVLLAASQTPGRDASDPVHEQVRNNVQLTLSNIGALLQKVKTIADDENRGIQALVSAAKYCRDQAAQLAPRPEPESLLTPVSSR  2002
             QLADVVK+GAAVIGPGQPDTQVE+L++CRD++  LR+V L++S+  GR + DPV+++VR+N QL +SNIG LLQ +++I +DE RGI++L  AA YCR+QA  L    +PE +LTP SSR
Sbjct  2319  QLADVVKQGAAVIGPGQPDTQVELLTSCRDISNALRNVFLSSSKAHGRPSLDPVYDEVRSNAQLVISNIGQLLQTLRSIEEDERRGIRSLELAATYCREQAKHLPSSRDPEGILTPPSSR  2438

Query  2003  RSAPSVANVSAVSGSSSLIARYLAPDDLARAASGPVQTAVSKAILAGNTQTQRDVLSTAATTRDVVTDLVSAATALLRCPEAVTETRTACVASTKGLAEEFAGLLDALKAVNSKPADHER  2122
             +S     + S  SGSSSLIARYLAPDDLARAASGPVQ+AVSKAILA NTQTQRD++   +TTRD VTDLV+AA  LLR  +  +ETR++C   T+    E  GL    K   S P   E
Sbjct  2439  KSG--AYSTSVASGSSSLIARYLAPDDLARAASGPVQSAVSKAILANNTQTQRDIVQATSTTRDAVTDLVAAARCLLRYSDIPSETRSSCAIVTR----EXXGLCTPRK---SGP---EN  2546

Query  2123  IGNAARRIADLSHTLLSQLDNLRESPRLVMYFRASSPEWRDVAEKFIGRHVAYHHHYVPTYTI---VGPGSTNRGLYVRPICYPFRPQKAIPEQDEDRSGDEAEFRIATAIDQLK-SILD  2238
             + N ARRIAD+SH+L++ +DNLRE PRL+MYFRASSPEWRDVAEKFIGRHVAYHHHYV  Y +    G GS+++ +++RP CYP   Q +  + E    DEAE RI  AI+QL  S+
Sbjct  2547  VSNTARRIADISHSLINLVDNLREGPRLIMYFRASSPEWRDVAEKFIGRHVAYHHHYVSNYALGGGSGSGSSHKSIFIRPTCYPL--QVSHDQGGEQIINDEAECRIVDAINQLNLSLEK  2664

Query  2239  C--EPPSDPTILQSAHSVAVATQSLIHTARSIGLAPPSDAVDRARRAGT----SHHGASLWRTELTVALATQDMCQLAQLCSAVLQEENAHPDSKDLFGTSTRLVPSKERLLAAVRRVAA  2352
             C  +     ++L SA SVAV+ Q+LI TAR++   PP+DAV+RAR A +    S   A LWRTEL V+LATQ +CQL++LCS  +    +    +    S  L+ S+ERLLAAVR+VA
```

```
Sbjct  2665  CLVDNKLAQSLLVSARSVAVSVQNLIRTARTVVSVPPNDAVERARTAESASQKSTSSAVLWRTELNVSLATQHLCQLSKLCSETVPSSTSTKLENNTGELSGGLILSRERLLAAVRQVAT  2784

Query  2353  ASAQLLMSAKSRRSKCSSADIQKLQVAGQLVKEATDRLSGVLHQAN--FTCDSTGLYVITPMPATLQNVIETHSKIHSHQSELDVLQQRLAQLQDNQ  2447
             A AQLL++AK R+    S+DI+KL+ AGQ VKE+TD L+  + +++   T   T +   T    +L   I T ++I S Q EL+ L+  LA+LQ  Q
Sbjct  2785  AGAQLLLAAKLRKEALMSSDIRKLKAAGQAVKESTDLLAEAVQKSDAISTTQRTEITWPTYTNQSLLQGIHTKARIQSQQIELEALESHLARLQQTQ  2881
```

C. sinensis vs *S. mansoni*

```
Score   Expect  Method  Identities     Positives      Gaps
491 bits(1265) 4e-158  Compositional matrix adjust.  286/557(51%)   380/557(68%)   20/557(3%)
Query  1903  QVEVLSACRDVATGLRDVLLAASQTPGRDASDPVHEQVRNNVQLTLSNIGALLQKVKTIADDENRGIQALVSAAKYCRDQAAQLAPRPEPESLLTPVSSRRSAPSVANVSAVSGSSSLIA  2022
             +VE+L++CRDVAT LR V L++ +  GR + DP++++VR+N Q+ +SN+G LLQ +K++ +DE RGI++L  AA YCR+QA  L    +PE LLTP SSR+S P     S  SGSSSLIA
Sbjct  5     KVELLTSCRDVATALRSVFLSSGKVQGRPSLDPIYDEVRSNAQVVISNVGQLLQTLKSVEEDERRGIRSLELAANYCREQAKLLPSSKDPEGLLTPNSSRKSGPRST--SVASGSSSLIA  122

Query  2023  RYLAPDDLARAASGPVQTAVSKAILAGNTQTQRDVLSTAATTRDVVTDLVSAATALLRCPEAVTETRTACVASTKGLAEEFAGLLDALKAVNSKP--ADHERIGNAARRIADLSHTLLSQ  2140
             RYLAPDDLARAA GPVQ+AVSKAILA NTQ  RD++ T++ TRD V DLV+AA  LLR  +   ETR++C   T+ LAEEFA LLDALK     P  +  E + N ARRIAD+SH+L++
Sbjct  123   RYLAPDDLARAAGGPVQSAVSKAILANNTQIHRDIVQTSSATRDAVADLVAAAKCLLRYSDIPPETRSSCAIVTRELAEEFAALLDALKNGLYIPRKSGPENVSNIARRIADISHSLINL  242

Query  2141  LDNLRESPRLVMYFRASSPEWRDVAEKFIGRHVAYHHHYVPTYTIVGPGSTNRGLYVRPICYPFRPQKAIPEQDEDRSGDEAEFRIATAIDQLKSILD-CEPPSD--PTILQSAHSVAVA  2257
             +D+LRE PRL+MYFRASSPEWRDVAEKFIGRHVAYHHHYV  Y + G   +++ +++RP CYP   Q +  +  E    DEAE RI  AI+QL S L+ C   +    ++L +A SVAV+
Sbjct  243   VDSLREGPRLIMYFRASSPEWRDVAEKFIGRHVAYHHHYVSNYALGGSSKSHKSIFIRPTCYPL--QISSEQTTEQTMNDEAECRIVDAINQLNSSLEKCSADNKLAQSLLVTARSVAVS  360

Query  2258  TQSLIHTARSIGLAPPSDAVDRARRA-GTSHH---GASLWRTELTVALATQDMCQLAQLCSAVLQEENAHPDSKDLFGTSTRLVPSKERLLAAVRRVAAASAQLLMSAKSRRSKCSSADI  2373
              Q+LI TAR++   PPS+AVDRAR A G S      A+LWRTEL V+LATQ +CQL++LCS  +    +     +    S  L+ S+ERLLAAVR+VA A AQLL++AK R+    SADI
Sbjct  361   VQNLIRTARTVISVPPSNAVDRARTAEGASQKSTSSAALWRTELNVSLATQHLCQLSKLCSETVPSITSQKLENNAGELSGGLILSRERLLAAVRQVATAGAQLLLAAKLRKEALMSADI  480

Query  2374  QKLQVAGQLVKEATDRLSGVLHQANFTCDSTG---LYVITPM---PATLQNVIETHSKIHSHQSELDVLQQRLAQLQ  2444
             +KL+ AGQ VKE+TD L+  + + +   +S+    + +  P     A LQ  I T ++I S Q EL+ L+ RLA L+
Sbjct  481   RKLKTAGQAVKESTDLLAETVQRGDIVSNSSTSPQIEITWPTYTNQALLQG-IHTKARIQSQQMELEALESRLASLK  556
```

C. sinensis vs *E. granulosus*

```
Score   Expect  Method  Identities     Positives      Gaps
1734 bits(4491)       0.0    Compositional matrix adjust.  1063/2421(44%) 1503/2421(62%) 185/2421(7%)
Query  100   DNFMMGGSHTFHYQEITPAQRALLVTIIEDVETLQAAKEQLYLSPDEDRI-IGIGSDEASKRWLSESIGASQAKVTDEVGAMNAAVAQALRSANRAEAYDSTGMSQVGLGVSQ--DPGDD  216
             D+F+ GG+  +  + +TPAQRAL VTI E++  L+  K++L + P  +R+ + +G DEAS+RWL ES+G ++ +V DE+GAMNAAVAQA+RSANRA   DS G   V  GV++  DP DD
Sbjct  546   DDFITGGTQYYVCKTMTPAQRALHVTIEENINALEEGKQRLDIGPPAERVLVNLGHDEASRRWLYESMGVTEVRVADEMGAMNAAVAQAVRSANRA---DSAG-GGVHFGVTEVTDPSDD  661

Query  217   LMMMQQSFRVVTIHFPAFVDDVKRVAVLRREVGAIRA-DQTGEPV---IAAQSEESSQNLLSAARHVADAFTDLLESARPLATGQPREEVVTDESTIRSQAVTT--GPVSSSSRKAIIDA  330
             LM +QQSFRVVT+HFPAFVD+VKRVAVLRRE G +RA D+  E +   I  QSE ++QNLL AAR VAD+FT LL++A PL T Q      TD +       VT      V+ SSRKAI++A
Sbjct  662   LMRVQQSFRVVTVHFPAFVDNVKRVAVLRRESGLLRAEDEPDERLRLDITDQSEANAQNLLGAARTVADSFTALLKAATPLTTRQ------TDYNLDTVNYVTEQGSQVAGSSRKAILEA  775

Query  331   ASRVGEASNDLLRHVMH-----EGD-----GEEDFEASSLMLTEEERLYKDQLLSLAKAVANTTASLVVKAKNLATQTSLDPEAQQHVIAAATQTGLCTSQLVACTKVLAPTIYQPSCQQ  440
             A+RVGEASND LR+V+      EG+      E  ++A  L L      Y+D+LL LAK VAN+TA LVVKAKNLATQTSLDP+ QQ V+ AAT TGLCTSQLVACTKVLAPTI+Q SCQQ
Sbjct  776   ANRVGEASNDFLRYVLQDTEVLEGEIVPEVDERAYQADLLAL------YQDKLLELAKEVANSTAVLVVKAKNLATQTSLDPDHQQLVVMAATNTGLCTSQLVACTKVLAPTIHQASCQQ  889

Query  441   QLSEAAREVSWAVDGVVQASRAAGARTADQPVQVQQP----VRTAVAETEMAATEVRDALDQLNAHLSKASAKTSSGDALDNFQLAYDQLQQYHTHDGQRMVASARRMAQATAQMIADIK  556
             QLSE+  EV+ AVD VV+ +R AG + A   + + +P    + +AV + E AATEVR +LD+LNAHL + S +   GD LD FQ AYD+L+Q    DG R+VA+ARR+Q TAQMI+D+K
Sbjct  890   QLSESTCEVATAVDDVVRVARGAG-QAAHDTINLSEPEVHEIDSAVMQVESAATEVRTSLDRLNAHLLRGSIRPYQGDTLDLFQQAYDELRQ--ETDGVRLVAAARRLSQVTAQMISDLK  1006

Query  557   AQAELAADDPDRQSRLFAAAKQLADATTILIASAKVCSTNPENPVTQEELRQAAESLNLIVHSAAADLLHRRLIRNLQAAARAAVTGATQLVNVSQVAAKRSRGNTYQVISDGKLVNGLI  676
```

```
                QAE    DP+RQ+RLFAAAKQLADATT LI  AK+CS+NP++   Q +L+ AA++L ++ +++AA+LL+ R+IR+LQAAARA V+ +  L+  S+ AA +SR N Y ++ + K V+ L+
Sbjct  1007   VQAETVEGDPERQTRLFAAAKQLADATTELINQAKICSSNPDSLTPQADLKHAADNLVVVAYASAAELLNARVIRSLQAAARAVVSASNNLITTSRYAANKSRANNYHILINEKAVSDLL  1126

Query  677    PRTVISIRESRACPDDPLTQVELISACERFVLPCESLVRSSRSVAPTVSDPTTQAALDNSTSQLSGAIEVLRTCLARLTPLTRQMQVDGALARLARLAVEAAAIETGLKQGTLAPLPDEK  796
              P+ V +IR  R  P+DPL Q+ELI ACE  + PC  L RS RS+ PT+ DP  Q+ALDNSTSQ+  A+E L+ CLAR++P+ RQ+Q+DGALA L R   EA  +E   K GTL  LPD+K
Sbjct  1127   PKMVSAIRFIRRDPEDPLAQLELICACEHAIQPCGQLARSCRSMVPTIGDPAVQSALDNSTSQMVVAVETLKACLARVSPIARQLQMDGALASLLRTVREAEELEDLSKAGTLTALPDDK  1246

Query  797    IDDCFNLLGTSVQDANVSTHELHSIVETMAQ-----QQRDMMSRSDVCGVPASMLATAIAGLVHATRGIVAHDAYDAADAGRSGAVGPSSDSTRLAVTQDTRQAVQLAYELVKAARDARF  911
              ++D + +L  S++DA  +T+E+ S V+++ Q     Q  +     D  G  A+ LA A++ L+ +TR  +  +      +G S A                 R A QLAY+LV  AR
Sbjct  1247   VEDRYRILKVSIRDAQNATNEVTSAVDSLEQDYAAPQSPNCDQGEDWLGSSANRLAGAMSDLLRSTRSCLVSEDRPIDSSGASAA----------------RTAAQLAYQLVCEAR----  1346

Query  912    AYDDGQMEALATVVSHSEQ------------LTVQLL-DTLGRC-----LRGLPGHREINEATNLIEKRREDLVQLSHEAPTRRVRWVEPSVYERAQSELTRAAVEFNQATGDLLSSYSP  1013
                   ++EAL   VS  +Q             +  +L+ D L R      L+ LPG +E++EA++L+ +RR+DLV+ + + PT     +E +  ER Q+E   AAVEFNQAT DL S Y+P
Sbjct  1347   -----KLEALQPTVSSPDQQADVLESNRNVAINCKLIGDNLNRALFECLLKALPGQKELSEASDLVGRRRKDLVRFA-DNPTVFEYPIEETRVERTQTEFAGAAVEFNQATADLTSCYTP  1460

Query  1014   GAFRRTTRRFTGAYDTLTVKGVDLCMSGVPPNEVPLPEPPPINRDLVDGLVNVSNHSYALMTEAGRVCARPDEQEIRDRFQTAARDVTESISQLLTICTSGVTAEQRDCEVALRRLEALR  1133
                FRR++ RF GAYD+L  KG+ L  +  P +         P    L+ GLV VS+ S  ++ +A +VC++P+ + +R +   AAR VT+SIS LL++  SGV     DC+  LRRLEA+R
Sbjct  1461   SNFRRSSVRFAGAYDSLVDKGLQLSRAKSPKD--------PTGPQLITGLVEVSDRSSEMLEDAKQVCSQPEAEPLRQKLHLAARSVTDSISHLLSVSVSGVVPGVADCDAELRRLEAMR  1572

Query  1134   PLLENPNRPVNQKTYCECVDEVARSITPLADSLRTMSNAAKEKHTQEFGAAVRQCSNSMCQLIEETAQAAYLIGLADPRSEPGRPSLVDPQLFMRSQREIQQVCDAICDPSITNRQIITL  1253
              PLLE P+RP NQ TY  CV   A+S+ PL+D +R   +  K   +T+ F + +R+ ++ +CQ++EET+QAAYLIG+A P SEPGR   VD   F R QR+I+ +C A+  P +T RQ++ L
Sbjct  1573   PLLEYPDRPTNQHTYQLCVSSFAQSLAPLSDGIRGTLDGIKSHNTEVFTSNIRKVTDCLCQVVEETSQAAYLIGVAHPTSEPGRGGPVDIAFFERLQRDIKNICRAMKSPEVTERQVVNL  1692

Query  1254   STEMARSAKTLCEACSAVSAQTNNPDARHQLNALTRETMQSITALIQRRGNVSTAAAGSQLAEVNGEWDEANRQVTLANARGVSSNVARLVHMVTQGPQFIGQAARVTDEAREAQQPVCS  1373
              + +M+ + ++L +AC+ ++AQ NN +AR +L  L  ++++S++ LIQR                + +W +   R++T  N   V+ N+ RLV  +T   +F G+ AR++ EAR AQQP+C
Sbjct  1693   ANDMSANVRSLRDACNTIAAQANNQEARRRLQNLADDSVKSMSELIQR----------------SADWSDEGRRITANNIHAVNGNIDRLVSFITSISEFGGEPARISSEARSAQQPICL  1796

Query  1374   AGLSSLNAAQAVLRAAQTLITNARL-GQPEQ-AFLSFSTASKELSESIKALAAAMREHAPGQLECQRVLQNINGLLQELQRAKMASMEGRLQPRRELNEEGFQKQLATCCRALLDAAPNV  1491
              AG + L + + V+ A++ ++  A    G+P   AF +F+ AS++L+++ K L A + E  PGQ ECQR L NI  LL EL+R KMA M+G   PR + NEEGF KQL    RA+ D A  V
Sbjct  1797   AGQACLESGRGVILASKHMLQTAETSGEPSNTAFGAFTAASRDLTDNTKHLLAVLNEQGPGQAECQRALLNIRRLLHELERDKMAMMDGVFTPRHDSNEEGFLKQLTISARAVRDMATPV  1916

Query  1492   GRAARSEAEQLGHSVRVVDSYLPGITSSAIAAASRSPLSSTQLVYLEHASTILEAADQLVRVARDAGGNPRATHLHPHLDEAVRGLMESCEDLLTALDDVASRQGHVSTLIDTINRSLAQ  1611
              GR A SEAE+LGH+VR +D  LPG+ SSA+ AASR+P SS QL+Y+EH  T+LE+ +QL+  A+ AGGNPR+  +    + + V+  ++SCE+LL+A++ VAS+Q  ++ L+ +  S A
Sbjct  1917   GRGATSEAERLGHAVRELDLLLPGLVSSALGAASRAPNSSAQLIYVEHTRTVLESVEQLISAAKQAGGNPRSDPIREAVGDGVKAQIDSCEELLSAIEGVASQQSFMAKLMAVLEESRAL  2036

Query  1612   TEEIIQVPVDARFADYQARLLRIARHMEQLTQAIQLRARQPPS--DGELAPLAHTLAQEYQEMCQTCKGAAATLPDARQADELRGAVRAVGMATAELVQATTAARIRAYDSHGDQQMLLS  1729
               ++  + P DA F DYQ+RLLR+ARHMEQ T+A    AR+P      E+  L    LAQ+Y E+C   + A  TL D  +A++LR AV+ +G  T  L+ AT    +    S  D +
Sbjct  2037   VDKPGRAPYDAHFTDYQSRLLRLARHMEQQTEATVRAARRPQESISQEMTGLVQNLAQDYAEICMISRDACETLRDPHEAEKLRWAVKNLGQTTRGLIMAT----VNVASSRPDTSL---  2149

Query  1730   RNQGI------AY---LDTRAEALDSKLRDLIALLDTQGPNTQACLQSASTVSGIIADLDTTILFASSGTLHGPVHDDVDALLGPDDYRPPYALPGAGEAIADRGSRFGNREYPEEGFGT  1840
               N+G       AY   LD  A A++ +LR+LIALL+ QGP TQACLQ ASTVSGIIADLDTTILFASSGTLH P+ DD D       +R  Y+      A++     FG RE  +  F +
Sbjct  2150   -NRGSVGAKPPAYLRSLDFSAGAMNDRLRELIALLEMQGPGTQACLQGASTVSGIIADLDTTILFASSGTLHPPIRDDADVHQLEAQHR--YS------ALSSGYDDFG-RE-AQGNFIS  2258

Query  1841   VRDSIVRTARALVDDTQSLVVSGTGEDQTRLATTAHVAVERVTQLADVVKRGAAVIGPGQPDTQVEVLSACRDVATGLRDVLLAASQTPGRDASDPVHEQVRNNVQLTLSNIGALLQKVKT  1960
              +R+SI RTA ALV DT+SLV   GEDQTRLA +  +AV  ++QLADVVK+GAA+IGPGQ D+QV +L A +D A  LR  LLAASQ  GR  +DP  +++        S++  LL+ V +
Sbjct  2259   IRESIHRTADALVQDTRSLVMCAGEDQTRLAGSTQMAVRNISQLADVVKQGAALIGPGQSDSQVLILGAAKDAAVALRSTLLAASQVQGRPKTDPAFKEMETYEGSVESSVAFLLKSVDS  2378

Query  1961   IADDENRGIQALVSAAKYCRDQAAQLAPRPEPESLLTPVSSRRSAPSVANVSAVSGSSSLIARYLAPDDLARAASGPVQTQTAVSKAILAGNTQTQRDVLSTAATTRDVVTDLVSAATALLR  2080
              I  +  RG +AL++ A  CR    QLA R  P S  T   +        +  + +S +SSL++RYLAPD+L+RA  GP++T + +A+   +T+ Q D L  A T R  V DLV+A   L R
Sbjct  2379   IDMESTRGTKALIATAHLCR----QLANR-MPSSAATAGQAVSLNDLGSATTLLSTTSSLVSRYLAPDELSRATEGPLRTTMDRAMATASTRRQDDALIFANTARHAVVDLVNACKNLQR  2493
```

```
Query  2081  CPEAVTETRTACVASTKGLAEEFAGLLDALKAV--NSKPADHERIGNAARRIADLSHTLLSQLDNLRESPRLVMYFRASSPEWRDVAEKFIGRHVAYHHHYVPTYTIVGPGSTNRGLYVR  2198
              PE   E R     + + +  E A LLDA+K V   + P D  R+  +A +I  LS+ L+  +D +R++PRLVMYFR S+PEWRDVAEK++GRH+A+HHHY       G   +NR    +
Sbjct  2494  QPEIKEEFRKGSADTVRKVVLETAELLDAVKCVIAEANPNDLNRVSASAGKITTLSNQLMRLIDRMRDNPRLVMYFRVSNPEWRDVAEKYLGRHIAFHHHYESCSDYDG---SNRATSSQ  2610

Query  2199  PICYPFRPQKAIPEQDEDRSGDEAEFRIATAIDQLKSILDCEPPSDPTILQSA-----HSVAVATQSLIHTARSIGLAPPSDAVDRARRAGTSHHGASLWRTELTVALATQDMCQLAQLC  2313
                C     Q  +       +G EA    +  ID    +L+ +        QS        V   TQ ++  AR+I LA   + V   + A TS   A+  R +L+VALAT+ +C+L +LC
Sbjct  2611  ISCL----QSQLDSTGRLLTGTEAALEASRRIDVALGLLEGD------FYQSMQRSTNQDVVHHTQPILSMARAITLA-TREFVQTVQLASTSKATANFGRLDLSVALATEQLCELTRLC  2719

Query  2314  SAVLQEENAHPDSKDLFGTSTRLVPSKERLLAAVRRVAAASAQLLMSAKSRRSKCSSADIQKLQVAGQLVKEATDRLSGVLHQAN--FTC----DSTGLYVITPMP--ATLQNVIETHSK  2425
              S    E    P +  L      R++PS+ERLL AVR+VA AS +LL + +S  ++ +SA++  +Q A ++VKE TD++S ++    +  F+         G  + TP+P  +  Q    + ++
Sbjct  2720  SETRSE---GPYTAPL-DDPIRVMPSRERLLNAVRQVATASGELLYAVQS-NAQITSANMTAVQTAARVVKEQTDKMSTMVRNKDPIFSHLPSQSGLGASIATPIPIVSPQQEGTNSSAQ  2834

Query  2426  IHSHQSELDVLQQRLAQLQDN  2446
              IH+ Q+ LD L+  + +L  N
Sbjct  2835  IHTEQAALDDLRNEVVRLNRN  2855
```

C. sinensis vs E. multilocularis

```
Score   Expect Method Identities     Positives      Gaps
1727 bits(4474)       0.0    Compositional matrix adjust.  1070/2502(43%) 1524/2502(60%) 210/2502(8%)
Query  29    VQHQRLPPPRRVGPKDGFVNGALEETIQPKMDDSIQMATG-----YMQTNGWSHQNLHTDRTDFVDGQWAERPMSPDNFMMGGSHTFHYQEITPAQRALLVTIIEDVETLQAAKEQLYLS  143
             ++ QRL  P++  P    +NG  ++ +    D+++    G     Y+Q        +     R     D          + D+F+ GG+  +  + +TPAQRAL VTI E++  L+  K++L +
Sbjct  480   IEKQRLQQPQQALPP--LLNGTAQQMVVNDDDNNMVGYIGTSERRYIQAGSIPTLSSEIGRIKVDD--------TTDDFITGGTQYYVCKTMTPAQRALHVTIEENINALEEGKQRLDIG  589

Query  144   PDEDRII-GIGSDEASKRWLSESIGASQAKVTDEVGAMNAAVAQALRSANRAEAYDSTGMSQVGLGVSQ--DPGDDLMMMQQSFRVVTIHFPAFVDDVKRVAVLRREVGAIRA-DQTGEP  259
             P  +R++  +G DEAS+RWL ES+G ++ +V DE+GAMNAAVAQA+RSANRA   DS G   V  GV++  DP DDLM +QQSFRVVT+HFPAFVD+VKRVAVLRRE G +RA D+  E
Sbjct  590   PPAERVLANLGHDEASRRWLDESMGVTEVRVADEMGAMNAAVAQAVRSANRA---DSAG-GGVHFGVTEVTDPSDDLMRVQQSFRVVTVHFPAFVDNVKRVAVLRRESGLLRAEDEPDER  705

Query  260   V---IAAQSEESSQNLLSAARHVADAFTDLLESARPLATGQPREEVVTDESTIRSQAVTTG--PVSSSSRKAIIDAASRVGEASNDLLRHVMH-----EGD-----GEEDFEASSLMLTE  364
             +   I  QSE ++QNLL AAR VAD+FT+LL++A PL T Q       TD +       VT     V+ SSRKAI++AA+RVGEASND LR+V+       EG+       E  ++A  L L
Sbjct  706   LRLDITDQSEANAQNLLGAARTVADSFTELLKAATPLTTRQ------TDYNLDTVNYVTEQGLQVAGSSRKAILEAANRVGEASNDFLRYVLQDTEVLEGEIVPEVDERAYQADLLAL--  817

Query  365   EERLYKDQLLSLAKAVANTTASLVVKAKNLATQTSLDPEAQQHVIAAATQTGLCTSQLVACTKVLAPTIYQPSCQQQLSEAAREVSWAVDGVVQASRAAGARTADQPVQVQQP----VRT  480
                 Y+D+LL LAK VAN+TA LVVKAKNLATQTSLD + QQ V+ AAT TGLCTSQLVACTKVLAPTI+Q SCQQQLSE+  EV+ +VD VV+ +R AG + A   +    +P    + +
Sbjct  818   ----YQDKLLELAKEVANSTAVLVVKAKNLATQTSLDLDHQQLVVMAATNTGLCTSQLVACTKVLAPTIHQASCQQQLSESTYEVATSVDDVVRVARGAG-QAAHDTINPSEPEVHEIDS  932

Query  481   AVAETEMAATEVRDALDQLNAHLSKASAKTSSGDALDNFQLAYDQLQQYHTHDGQRMVASARRMAQATAQMIADIKAQAELAADDPDRQSRLFAAAKQLADATTILIASAKVCSTNPENP  600
             AV + E AATEVR +LD+LNAHL + S +   GD LD FQ AYD+L+Q    DG R+VA+ARR+Q TAQMI+D+K QAE    DP+RQ+RLFAAAKQLADATT LI  AK+CS+NP++
Sbjct  933   AVMQVESAATEVRTSLDRLNAHLLRGSIRPYQGDTLDLFQQAYDELRQ--ETDGVRLVAAARRLSQVTAQMISDLKMQAETVEGDPERQTRLFAAAKQLADATTELINQAKICSSNPDSL  1050

Query  601   VTQEELRQAAESLNLIVHSAAADLLHRRLIRNLQAAARAAVTGATQLVNVSQVAAKRSRGNTYQVISDGKLVNGLIPRTVISIRESRACPDDPLTQVELISACERFVLPCESLVRSSRSV  720
               Q +L+ AA++L ++ +++AA+LL+ R+IR+LQAAARA V+ +  L+  S+ AA +SR N Y ++ + K V+ L+P+ V +IR  R  P+DPL Q+ELI ACE  + PC  L RS RS+
Sbjct  1051  TPQADLKHAADNLVVVAYASAAELLNARVIRSLQAAARAVVSASNNLITTSRYAANKSRANNYHILINEKTVSDLLPKIVSAIRFIRRDPEDPLAQLELICACENAIQPCNQLARSCRSM  1170

Query  721   APTVSDPTTQAALDNSTSQLSGAIEVLRTCLARLTPLTRQMQVDGALARLARLAVEAAAIETGLKQGTLAPLPDEKIDDCFNLLGTSVQDANVSTHELHSIVETMAQ-----QQRDMMSR  835
              PT+ DP  Q+ALDNSTSQ+  A+E L+ CLAR++P+ RQ+Q+DGALA L R   EA  +E   K GTL  LPD+K++D + +L  S++DA  +T+E+ S V+++ Q     Q  +
Sbjct  1171  VPTIGDPAVQSALDNSTSQMVVAVETLKACLARVSPIARQLQMDGALASLLRTVREAEELEDLAKAGTLTALPDDKVEDRYRILKVSIRDARNATNEVTSAVDSLEQDYAAPQSPNCDQG  1290

Query  836   SDVCGVPASMLATAIAGLVHATRGIVAHDAYDAADAGRSGAVGPSSDSTRLAVTQDTRQAVQLAYELVKAARDARFAYDDGQMEALATVVSHSEQ---------------------LTVQ  934
              D  G  A+ LA A++ L  + R  +  +      +G  A                R A QLAY+LV  AR         ++EAL   VS  +Q                     L
Sbjct  1291  EDWLGSSANRLAGAMSDLSRSIRSCLVSEDRPIDSSGAFAA----------------RTAAQLAYQLVCEAR---------KLEALQPTVSSPDQQADVLESNRNVAINCKLIGDNLNCA  1385
```

```
Query  935   LLDTLGRCLRGLPGHREINEATNLIEKRREDLVQLSHEAPTRRVRWVEPSVYERAQSELTRAAVEFNQATGDLLSSYSPGAFRRTTRRFTGAYDTLTVKGVDLCMSGVPPNEVPLPEPPP  1054
             LL+ L   L+ LPG +E++EA++L+ +RR+DLV+ + + PT     +E +  ER Q+E   AAVEFNQAT DL S Y+P  FRR++ RF GAYD+L  KG+ L  +  P +        P
Sbjct  1386  LLECL---LKALPGQKELSEASDLVGRRRKDLVRFA-DNPTVFEYPIEETRVERTQTEFAGAAVEFNQATADLTSCYTPSNFRRSSVRFAGAYDSLVNKGLQLSRAKSPKD--------P  1493

Query  1055  INRDLVDGLVNVSNHSYALMTEAGRVCARPDEQEIRDRFQTAARDVTESISQLLTICTSGVTAEQRDCEVALRRLEALRPLLENPNRPVNQKTYCECVDEVARSITPLADSLRTMSNAAK  1174
                 L+ GLV VS+ S  ++ +A +VC++P+ + +R +   AAR VT+SIS +L+I  SGV      DC+  LRRLEA+RPLLE P RP NQ TY  CV   A+S+ PL+D +R   +  +
Sbjct  1494  TGPQLITGLVEVSDRSSEMLEDAKQVCSQPEAEPLRQKLHLAARSVTDSISHILSISVSGVVPGVADCDAELRRLEAMRPLLEYPGRPTNQHTYQLCVSSFAQSLAPLSDGIRGTLDGVR  1613

Query  1175  EKHTQEFGAAVRQCSNSMCQLIEETAQAAYLIGLADPRSEPGRPSLVDPQLFMRSQREIQQVCDAICDPSITNRQIITLSTEMARSAKTLCEACSAVSAQTNNPDARHQLNALTRETMQS  1294
               +T+ F + +R+ ++ +C ++EET+QAAYLIG+A P SEPGR   VD   F R QR+I+ +C A+  P +T RQ++ L+ +M+ + ++L +AC+ ++AQ +N +AR +L  L  ++++S
Sbjct  1614  SHNTEVFTSNIRKVTDCLCHVVEETSQAAYLIGVAHPTSEPGRGGPVDIAFFERLQRDIKNICRAMKSPEVTERQVVNLANDMSANVRSLHDACNTIAAQASNQEARRRLQNLADDSVKS  1733

Query  1295  ITALIQRRGNVSTAAAGSQLAEVNGEWDEANRQVTLANARGVSSNVARLVHMVTQGPQFIGQAARVTDEAREAQQPVCSAGLSSLNAAQAVLRAAQTLITNARL-GQPEQ-AFLSFSTAS  1412
             ++ LIQ                + +W +   R++T  N   V+ N+ RLV  +T    +F G+ AR++ EAR AQQP+C AG + L + + V+ A++ ++  A    G+P   AF +F+ AS
Sbjct  1734  MSELIQH----------------SADWSDEGRRITANNIHAVNGNIDRLVSFITSISEFGGEPARISSEARSAQQPICLAGQACLESGRGVILASKHMLQTAETSGEPSNTAFGAFTAAS  1837

Query  1413  KELSESIKALAAAMREHAPGQLECQRVLQNINGLLQELQRAKMASMEGRLQPRRELNEEGFQKQLATCCRALLDAAPNVGRAARSEAEQLGHSVRVVDSYLPGITSSAIAAASRSPLSST  1532
             ++L+++ K L A + E  PGQ ECQR L NI  LL EL+R KMA M+G   PR + NEEGF KQL     RA+ D A +VGR A SEAE+LGH+VR +D  LPG+ SSA+ AASR+P SS
Sbjct  1838  RDLTDNTKHLLAVLNEQGPGQAECQRTLLNIRRLLHELERDKMAMMDGVFTPRHDSNEEGFLKQLTISTRAVRDMATSVGRGATSEAERLGHAVRELDLLLPGLVSSALGAASRAPNSSA  1957

Query  1533  QLVYLEHASTILEAADQLVRVARDAGGNPRATHLHPHLDEAVRGLMESCEDLLTALDDVASRQGHVSTLIDTINRSLAQTEEIIQVPVDARFADYQARLLRIARHMEQLTQAIQLRARQP  1652
             QL+Y+EH  T+LE+ +QL+  A+ AGGNPR+  +   +D+ V+  ++SCE+LL+A++ VAS+Q  ++ L+  +  S A  ++  + P DA F DYQ+RLLR+ARHMEQ T+A    AR+P
Sbjct  1958  QLIYVEHTRTVLESVEQLISAAKQAGGNPRSDPIREAVDDGVKAQIDSCEELLSAIEGVASQQSFLAKLMAVLEESRALVDKPGRAPYDAHFTDYQSRLLRLARHMEQQTEATVRAARRP  2077

Query  1653  --PSDGELAPLAHTLAQEYQEMCQTCKGAAATLPDARQADELRGAVRAVGMATAELVQATTAARIRAYDSHGDQQMLLSRN----QGIAY---LDTRAEALDSKLRDLIALLDTQGPNTQ  1763
                +  E+  L   LAQ+Y E+C    + A  TL D  +A++LR AV+ +G  T  L+ AT    +    S  D     SR     +  AY   LD  A A++ +LR+LIALL+ QGP TQ
Sbjct  2078  QESTSQEMTGLVQNLAQDYAEICMISRDACETLRDPHEAEKLRWAVKNLGQTTRGLIMAT----VNVSSSRPDPS--FSRGSVGPKPPAYLRSLDFSAGAMNDRLRELIALLEMQGPGTQ  2191

Query  1764  ACLQSASTVSGIIADLDTTILFASSGTLHGPVHDDVDALLGPDDYRPPYALPGAGEAIADRGSRFGNREYPEEGFGTVRDSIVRTARALVDDTQSLVSGTGEDQTRLATTAHVAVERVTQ  1883
             ACLQ ASTVSGIIADLDTTILFASSGTLH P+ DD D       +R     PG  +         FG RE  +  F ++R+SI RTA ALV DT+SLV G GEDQTRLA +   AV  ++Q
Sbjct  2192  ACLQGASTVSGIIADLDTTILFASSGTLHPPIRDDADVHQLEAHHRYSALSPGYDD--------FG-RE-AQGNFISIRESIHRTADALVQDTRSLVMGAGEDQTRLAGSTQKAVRNISQ  2301

Query  1884  LADVVKRGAAVIGPGQPDTQVEVLSACRDVATGLRDVLLAASQTPGRDASDPVHEQVRNNVQLTLSNIGALLQKVKTIADDENRGIQALVSAAKYCRDQAAQLAPRPEPESLLTPVSSRR  2003
             LADVVK+GAA+IGPGQ D+QV +L A +D A  LR  LLAASQ  GR +DP  +++         S++  LL+ V +I  +  RG +AL++ A  CR    QLA R     + +  ++ +
Sbjct  2302  LADVVKQGAALIGPGQSDSQVLILGAAKDAAVALRSTLLAASQVQGRPKTDPAFKEMEAYEGSVESSVTFLLKSVDSIDMESTRGTKALIATAHLCR----QLANR-----MPSSAATAK  2412

Query  2004  SAPSVANVSAV----SGSSSLIARYLAPDDLARAASGPVQTAVSKAILAGNTQTQRDVLSTAATTRDVVTDLVSAATALLRCPEAVTETRTACVASTKGLAEEFAGLLDALKAV--NSKP  2117
              A S+ ++ +     S +SSL++RYLAPD+L+RA  GP++T + +A+   +T+ Q D L  A T R  V DLV+A   L R PE   E R     + + +  E A LLDA+K V   + P
Sbjct  2413  QAVSLNDLGSATTLLSTTSSLVSRYLAPDELSRATEGPLRTTMDRAMATASTRRQDDALIFANTARHAVVDLVNACKNLQRQPEIKEEFRKGSSDTVRKVVLETAELLDAVKCVIAEANP  2532

Query  2118  ADHERIGNAARRIADLSHTLLSQLDNLRESPRLVMYFRASSPEWRDVAEKFIGRHVAYHHHYVPTYTIVGPGSTNRGLYVRPICYPFRPQKAIPEQDEDRSGDEAEFRIATAIDQLKSIL  2237
              D  R+  +A +I  +S  L+  +D +R++PRLVMYFR S+PEWRDVAEK+ GRH+A+HHHY        G   +R    +  C     Q  +        +G+EA    +  ID    +L
Sbjct  2533  NDLNRVSASAGKITTMSSQLMRLIDRMRDNPRLVMYFRVSNPEWRDVAEKYLGRHIAFHHHYESCSDYDG---SYRATSSQISCL----QSQLDSTGRLLTGNEAALEASRRIDVALGLL  2645

Query  2238  DCEPPSDPTILQSA-----HSVAVATQSLIHTARSIGLAPPSDAVDRARRAGTSHHGASLWRTELTVALATQDMCQLAQLCSAVLQEENAHPDSKDLFGTSTRLVPSKERLLAAVRRVAA  2352
             + +         QS        V    TQ ++  AR++ LA     + V    + A TS    A+    R +L+VALAT+  +C+L   +LCS     E      P  +  L      R++PS+ERLL  AVR+VA
Sbjct  2646  EGD------FYQSMQRTTNQDVVHHTQPILSMARAVTLA-TREFVQTVQLASTSKAAANFGRLDLSVALATEQLCELTRLCSETRSE---GPYTAPL-DDPVRVMPSRERLLNAVRQVAT  2754

Query  2353  ASAQLLMSAKSRRSKCSSADIQKLQVAGQLVKEATDRLSGVLHQAN--FTC--DSTGL--YVITPMPATLQNVIETHS--KIHSHQSELDVLQQRLAQLQDN  2446
             AS +LL + +S  S+ +SA++  +Q A + VKE TD++S ++   +  F+     +GL   + TP+P        +T+S  +IH+ Q  LD LQ  + +L  N
```

```
Sbjct  2755  ASGELLYAVQS-NSQITSANMTAVQTAARAVKEQTDKMSTMVRNKDPIFSHLPSQSGLEASIATPIPIVSPQQEDTNSSAQIHTEQEALDDLQNEVERLNRN  2855
```

C. sinensis vs H. microstoma

```
Score  Expect Method Identities     Positives      Gaps
1696 bits(4392)       0.0    Compositional matrix adjust.  1030/2357(44%) 1468/2357(62%) 155/2357(6%)
Query  98    SPDNFMMGGSHTFHYQEITPAQRALLVTIIEDVETLQAAKEQLYLSPDEDRI-IGIGSDEASKRWLSESIGASQAKVTDEVGAMNAAVAQALRSANRAEAY-DSTGMSQVGLGVSQDPGD  215
             S D+F  GG+  +  +++TPAQRAL +TI E++  LQ  K++L + P  +R+ I +G DEAS+RWL ES+GA++ +V DE+GAMNAAVAQA+RSANRA+A   S G +  G+    DP D
Sbjct  533   STDDFCTGGTQYYICKKMTPAQRALHITIEENISALQEGKQRLDIGPPTERVLINLGHDEASRRWLDESMGATEVRVADEMGAMNAAVAQAVRSANRADAVVRSVGPTMYGVAEITDPAD  652

Query  216   DLMMMQQSFRVVTIHFPAFVDDVKRVAVLRREVGAIRA-DQTGEPV---IAAQSEESSQNLLSAARHVADAFTDLLESARPLATGQPREEVVTDESTIRSQAVTTGPVSSSSRKAIIDAA  331
             DLM MQQSFRVVT+HFPAFVD+VKRVAVLRRE   +RA D+  E V   I  +SE ++QNLL AAR VAD+FT+LL++A PL T Q   ++  D     ++     P   SSRKAI++AA
Sbjct  653   DLMRMQQSFRVVTVHFPAFVDNVKRVAVLRRESALLRAEDEPDERVRHGITDESEANAQNLLGAARTVADSFTELLKAATPLTTRQTEYDL--DVVNYVTEQGVQAP--GSSRKAILEAA  768

Query  332   SRVGEASNDLLRHVMHEG---DGE----ED---FEASSLMLTEEERLYKDQLLSLAKAVANTTASLVVKAKNLATQTSLDPEAQQHVIAAATQTGLCTSQLVACTKVLAPTIYQPSCQQQ  441
             +RVGEASND LR+VM +    DGE    ED   ++A  L +      ++D+LL LAK VAN+TA LVVKAKNLATQT+LD E QQ V+  ATQTGLCTSQLVACTKVLAPTI+Q SCQQQ
Sbjct  769   NRVGEASNDFLRYVMQDSEVTDGEIVPEEDEGVYQADQLAI------FQDKLLELAKEVANSTAVLVVKAKNLATQTNLDSEHQQMVVMNATQTGLCTSQLVACTKVLAPTIHQASCQQQ  882

Query  442   LSEAAREVSWAVDGVVQASRAAGARTADQ----PVQVQQPVRTAVAETEMAATEVRDALDQLNAHLSKASAKTSSGDALDNFQLAYDQLQQYHTHDGQRMVASARRMAQATAQMIADIKA  557
             LSE+AR+VS AVD VV+ +R++G    D+       VQ+ V  AV E E AA EVR +LD+LNAHL   SA+   GD+LD FQ AYD LQQ    DG R+VA+ARR+Q TAQMI ++K
Sbjct  883   LSESARDVSNAVDSVVRTARSSGQTAHDRMDLSDADVQE-VNAAVIEVEDAAVEVRTSLDRLNAHLLSGSARPYQGDSLDLFQQAYDALQQ--ETDGIRLVAAARRLSQVTAQMINNLKV  999

Query  558   QAELAADDPDRQSRLFAAAKQLADATTILIASAKVCSTNPENPVTQEELRQAAESLNLIVHSAAADLLHRRLIRNLQAAARAAVTGATQLVNVSQVAAKRSRGNTYQVISDGKLVNGLIP  677
             QAE    DP+RQ+RLFAAAKQLAD+TT LI  AKVCS++P++PV +  L+ +A+ L ++ +++AA+LL+ R+IR+LQAAARA V+ +  L+  S+VA+ +SR N Y + SD + VN L+P
Sbjct  1000  QAETLEGDPERQTRLFAAAKQLADSTTDLINQAKVCSSDPDSPVRKAALKHSADDLVVVAYASAAELLNARVIRSLQAAARAVVSASNNLITTSRVASSKSRSNNYHMASDERAVNDLLP  1119

Query  678   RTVISIRESRACPDDPLTQVELISACERFVLPCESLVRSSRSVAPTVSDPTTQAALDNSTSQLSGAIEVLRTCLARLTPLTRQMQVDGALARLARLAVEAAAIETGLKQGTLAPLPDEKI  797
             + V +IR  R  P+DPL Q+ELI  C++ V PC  L RS R + PT+SD   Q ALD S+SQ+  A+E L+ CLAR +P+ RQ+Q+DGALA L R   EA  IE   K GTL  LPD+++
Sbjct  1120  KMVSAIRFIRRDPEDPLAQLELICTCKQAVQPCSQLARSCRQIVPTISDAALQNALDGSSSQMVVAVETLKACLARASPIARQLQMDGALASLLRTVREAEEIEKLAKAGTLTALPDDRV  1239

Query  798   DDCFNLLGTSVQDANVSTHELHSIVETMAQ-----QQRDMMSRSDVCGVPASMLATAIAGLVHATRGIVAHDAYDAADAGRSGAVGPSSDSTRLAVTQDTRQAVQLAYELVKAARDARFA  912
             +D +  L   V+D   S  E+ S V+T+AQ      Q  +      D  G  A+ LA+ ++ LV +TR  +A +      DA  +  V               R + QLAY+LV  AR
Sbjct  1240  EDRYQTLKVGVRDTQNSISEVLSAVDTLAQDYASPQSPNRDQSEDWLGSSANRLASGVSDLVRSTRSCLAAEER-PVDASLASCV---------------RSSAQLAYQLVCEARKLEPV  1343

Query  913   YD-----DGQMEALAT---VVSHSEQLTVQLLDTLGRC-LRGLPGHREINEATNLIEKRREDLVQLSHEAPTRRVRWVEPSVYERAQSELTRAAVEFNQATGDLLSSYSPGAFRRTTRRF  1023
                    D Q + L +    V ++ + +   L  TL  C L+ LPG +E++EA+NL+++RR+DLV+ S E P+      +E +  E+ QSE T AAV+ NQAT DL S Y+P  F +++ RF
Sbjct  1344  QQANLTPDQQFDVLESNRNVNANCKLVGDNLNRTLSECLLKALPGQKELSEASNLVQRRRKDLVRFSEE-PSVFEYPIEETRIEKIQSEFTGAAVDLNQATADLTSCYTPVNFCKSSVRF  1462

Query  1024  TGAYDTLTVKGVDLCMSGVPPNEVPLPEPPPINRDLVDGLVNVSNHSYALMTEAGRVCARPDEQEIRDRFQTAARDVTESISQLLTICTSGVTAEQRDCEVALRRLEALRPLLENPNRPV  1143
              GAYD L  KG+ L  +    +        P +  L+ GLV+VS+ S  ++  A ++C++P+ + +R R   AAR VT+SIS LL++  SGV     DC+ ALRRLE++RPLLE P+RPV
Sbjct  1463  AGAYDNLVDKGIQLSRTRSSKD--------PSSNQLISGLVDVSDRSDEMLESAKQLCSQPEAEPLRQRLLGAARSVTDSISHLLSVSASGVIPGVADCDAALRRLESMRPLLEYPDRPV  1574

Query  1144  NQKTYCECVDEVARSITPLADSLRTMSNAAKEKHTQEFGAAVRQCSNSMCQLIEETAQAAYLIGLADPRSEPGRPSLVDPQLFMRSQREIQQVCDAICDPSITNRQIITLSTEMARSAKT  1263
             N   Y  CV   A+S+TPL++ +R   +  K  +T+ F + VR+ ++S+CQL EET+QAAYLIG++ P SEPGR  L+D   F R QR+I+ +C +I  P +T R++++L+ +M+ + ++
Sbjct  1575  NSHIYQTCVHACAQSLTPLSEGIRGTLDGVKSHNTEVFTSNVRRVTDSLCQLAEETSQAAYLIGVSHPTSEPGRVGLIDIAFFERLQRDIRNICRSISSPQVTEREVVSLANDMSANMRS  1694

Query  1264  LCEACSAVSAQTNNPDARHQLNALTRETMQSITALIQRRGNVSTAAAGSQLAEVNGEWDEANRQVTLANARGVSSNVARLVHMVTQGPQFIGQAARVTDEAREAQQPVCSAGLSSLNAAQ  1383
             L +AC+++ A++ NP+A+ +L AL+ +  QS++ LIQR                + +W+   R+ T  +A  V++N+ RL++ ++   +F  + +R+  E +  QQP+C+AG + L + +
Sbjct  1695  LRDACNSIRAKSTNPEAQRKLQALSNDATQSMSDLIQR----------------SADWSDEGRRATNNHAHAVNTNIDRLMNYLSSTNEFTSEPSRMGGEIKAVQQPICAAGQACLESGK  1798

Query  1384  AVLRAAQTLITNAR-LGQP-EQAFLSFSTASKELSESIKALAAAMREHAPGQLECQRVLQNINGLLQELQRAKMASMEGRLQPRRELNEEGFQKQLATCCRALLDAAPNVGRAARSEAEQ  1501
```

```
                V+ A++ ++  A    QP + AF +F+ AS++L+++ K L + +   APGQ ECQR L NI  LL EL+R KM+ M+G   PR +  EEGF KQL+   RAL D AP VG  A SEAE+
Sbjct  1799  GVILASKHMLQTAESKAQPSDTAFAAFTAASRDLTDNTKHLLSVLNAQAPGQAECQRALLNIRRLLHELERDKMSIMDGAFAPRHQATEEGFLKQLSVNSRALRDIAPQVGHGATSEAEK  1918

Query  1502  LGHSVRVVDSYLPGITSSAIAAASRSPLSSTQLVYLEHASTILEAADQLVRVARDAGGNPRATHLHPHLDEAVRGLMESCEDLLTALDDVASRQGHVSTLIDTINRSLAQTEEIIQVPVD  1621
             LGH+VR +D  LPG+ SSA+ AASRS   S Q+ YLEH  T++E+A+QLV  A+ AGGNPR+ +L   +++ V   + SCE+LL+A++ ++S+Q  ++ L+  ++ S    ++  ++P D
Sbjct  1919  LGHTVRELDLLLPGLVSSALGAASRSSNPSAQMTYLEHTRTVIESAEQLVSAAKQAGGNPRSDNLRVTVNDCVNAQVASCEELLSAIEGISSQQNFINKLMAILDESQNLVDKPGRIPSD  2038

Query  1622  ARFADYQARLLRIARHMEQLTQAIQLRARQP--PSDGELAPLAHTLAQEYQEMCQTCKGAAATLPDARQADELRGAVRAVGMATAELVQATTAARIRAYDSHGDQQMLLSRN------QG  1733
             A F DYQ+RLLR+ RHMEQ T+     AR+P   +  E+  L  +LAQ+Y E+C   + A  T+ D  +A++LR AV+ +G  T  L+ AT    + A  S  D  L  +
Sbjct  2039  AHFTDYQSRLLRLVRHMEQQTEVTVRTARRPQETASQEITGLVQSLAQDYAEICMISRDACETIRDPHEAEKLRWAVKNLGQTTRGLILAT----VNAASSKSDTSSLTRKGALVRPPAS  2154

Query  1734  IAYLDTRAEALDSKLRDLIALLDTQGPNTQACLQSASTVSGIIADLDTTILFASSGTLHGPVHDDVDALLGPDDYRPPYALPGAGEAIADRGSRFGNREYPEEGFGTVRDSIVRTARALV  1853
             I  LD  A A++ +LR+LIALL+ QGP TQACLQ ASTVSGIIADLDTTILFASSGTLH P+ DDVD  +    + +  Y+    +G    DR +         +  F ++R+SI RTA ALV
Sbjct  2155  IRSLDFSAGAMNERLRELIALLEAQGPGTQACLQGASTVSGIIADLDTTILFASSGTLHPPIRDDVD--MQNLESQQRYSAISSGYDAFDREA--------QSHFISIRESIHRTADALV  2264

Query  1854  DDTQSLVSGTGEDQTRLATTAHVAVERVTQLADVVKRGAAVIGPGQPDTQVEVLSACRDVATGLRDVLLAASQTPGRDASDPVHEQVRN--NVQLTLSNIGALLQKVKTIADDENRGIQA  1971
              DT+SLV G GEDQTRLA +  +AV  ++QLADVVK+GAA+IGPGQ D+QV++LS  +D A  LR+ LL A+Q       DPV  +V+   N   T SN   LL+ V  +  + NRG +A
Sbjct  2265  QDTRSLVLGAGEDQTRLAGSTQMAVRNISQLADVVKQGAALIGPGQSDSQVQILSTAKDTAVALRNTLLTAAQVQSGSKKDPVFNEVKTYENEVGTCSN--RLLKAVDAVDMEANRGSKA  2382

Query  1972  LVSAAKYCRDQAAQLAPRPEPESLLTPVSSRRSAPSVANVSA----VSGSSSLIARYLAPDDLARAASGPVQTAVSKAILAGNTQTQRDVLSTAATTRDVVTDLVSAATALLRCPEAVTE  2087
             L+S A  CR    QLA R        P     SAPS  ++ +    +S +SSL++RYL PD+LARAA GP++  V KA+ A +TQ Q D L  A T R  + DLV+    L +  E  E
Sbjct  2383  LISTAHLCR----QLASR-------MP----NSAPSFNDLGSATTLMSTTSSLVSRYLTPDELARAAEGPLRAIVDKAVTAASTQRQDDALVCANTARHAILDLVNTCKNLQKQQEIKEE  2487

Query  2088  TRTACVASTKGLAEEFAGLLDDALKAV----NSKPADHERIGNAARRIADLSHTLLSQLDNLRESPRLVMYFRASSPEWRDVAEKFIGRHVAYHHHYVPTYTIVGPGSTNRGLYVRPICYP  2203
              R     + K +  E A L+D++K V    N+K  D  R+ N+A +I  LS  L+  +D +R+SPRLVMYFR +SPEWRDVAEK+GRH+A+H+HY    +       ++ + +R  C
Sbjct  2488  FRKQSGDTVKKIVLETAELMDSVKGVIAGENTK--DSSRVSNSAAKIGALSSQLMRLIDKMRDSPRLVMYFRVNSPEWRDVAEKYLGRHIAFHNHY---ESCTEQDGFSKSISIRTSCLQ  2602

Query  2204  FRPQKAIPEQDEDRSGDEAEFRIATAIDQLKSILDCEPPSDPTILQSAHSVAVATQSLIHTARSIGLAPPSDAVDRARRAGTSHHGASLWRTELTVALATQDMCQLAQLCSAVLQEENAH  2323
              +    +        +  EA  RI  A+  L+S L+ +   +P        + V TQ ++  ARS  LA   + V   + A  S    +L R  L+VALA + +C L++LCS    E
Sbjct  2603  SQLDTKVFLMSGAEAAIEAIHRIDIALGLLESDLN-KSKQEPI----NQDIVVHTQPILSLARSTTLA-IREFVQTVQLALASRESTNLGRLNLSVALAIEQLCDLSRLCSETRSEGPYT  2716

Query  2324  PDSKDLFGTSTRLVPSKERLLAAVRRVAAASAQLLMSAKSRRSKCSSADIQKLQVAGQLVKEATDRLSGVLHQANFT  2400
                 D     TR++PS++RLL AVR+VA+AS +LL + +S   + SS ++  +Q A + +KE TDR+S ++   + T
Sbjct  2717  APVDD----PTRVMPSRQRLLNAVRQVASASGELLCAVQS-NPQISSTNLASVQAAARAIKEQTDRMSQLIRNKDPT  2788
```

C. sinensis vs T. solium

```
Score   Expect Method Identities     Positives      Gaps
1721 bits(4457)       0.0    Compositional matrix adjust.  1064/2416(44%) 1500/2416(62%) 166/2416(6%)
Query  100   DNFMMGGSHTFHYQEITPAQRALLVTIIEDVETLQAAKEQLYLSPDEDRI-IGIGSDEASKRWLSESIGASQAKVTDEVGAMNAAVAQALRSANRAEAYDSTGMSQVGLGVSQ--DPGDD  216
             D+F+ GG+  +   + +TPAQRAL VTI E++  L+  K++L + P  +R+ + +G DEAS+RWL ES+G ++ +V DE+GAMNAAVAQA+RSANR    DS G   V  GV++  DP DD
Sbjct  610   DDFITGGTQYYVCKTMTPAQRALHVTIEENISALEEGKQRLDIGPPTERVLVNLGHDEASRRWLDESMGVTEVRVADEMGAMNAAVAQAVRSANRV---DSMGGGGVRFGVAEVTDPSDD  726

Query  217   LMMMQQSFRVVTIHFPAFVDDVKRVAVLRREVGAIRA-DQTGEPV---IAAQSEESSQNLLSAARHVADAFTDLLESARPLATGQPREEVVTDESTIRSQAVTTGPVSSSSRKAIIDAAS  332
             LM MQQSFRVVT+HFPAFVD+VKRVAVLRRE G +RA D+  E +   I  QSE ++QNLL AAR+VAD+FT+LL++A PL T Q       D  T+         V+ SSRKAI++AA+
Sbjct  727   LMRMQQSFRVVTVHFPAFVDNVKRVAVLRRESGLLRAEDEPDERLRHDITDQSEANAQNLLGAARNVADSFTELLKAATPLTTRQTD----YDLDTVNYLTEQGSQVAGSSRKAILEAAN  842

Query  333   RVGEASNDLLRHVMHEGDGEEDFEASSLMLTEEER--------LYKDQLLSLAKAVANTTASLVVKAKNLATQTSLDPEAQQHVIAAATQTGLCTSQLVACTKVLAPTIYQPSCQQQLSE  444
             RVGEASND LR+V+ + +  E      ++   +ER        LY+D+LL LAK VAN+TA LVVKAKNLATQT+LDP+ QQ V+ +AT TGLCTSQLVACTKVLAPTI+Q SCQQQLSE
Sbjct  843   RVGEASNDFLRYVLQDTEVVE----GEIVPEVDERAYHVDLLALYQDKLLELAKEVANSTAVLVVKAKNLATQTNLDPDHQQLVVISATNTGLCTSQLVACTKVLAPTIHQASCQQQLSE  958
```

```
Query  445   AAREVSWAVDGVVQASRAAGARTADQPVQVQQP----VRTAVAETEMAATEVRDALDQLNAHLSKASAKTSSGDALDNFQLAYDQLQQYHTHDGQRMVASARRMAQATAQMIADIKAQAE  560
             +A EV+ AVD VV+ +R AG    D  + + +P    V +AV E E AATEVR +LD+LNAHL + S +   GD LD FQ AYD+L+Q    DG R+VA+ARR++Q TAQMI+D+K QAE
Sbjct  959  SACEVATAVDDVVRIARGAGQAVHDT-IGLSEPEIREVSSAVMEVENAATEVRTSLDRLNAHLVRGSIRPYQGDTLDLFQQAYDELRQ--ETDGVRLVAAARRLSQVTAQMISDLKVQAE  1075

Query  561   LAADDPDRQSRLFAAAKQLADATTILIASAKVCSTNPENPVTQEELRQAAESLNLIVHSAAADLLHRRLIRNLQAAARAAVTGATQLVNVSQVAAKRSRGNTYQVISDGKLVNGLIPRTV  680
                 DP+RQ+RLFAAAKQLADATT LI  AK CS++P++   Q +L+ AA+ L ++ +++AA+LLH R+IR+LQAAARA V+ +  L+  S  AA +SR N Y +++D K V+ L+P+ V
Sbjct  1076 SVEGDPERQTRLFAAAKQLADATTDLINQAKTCSSDPDSLTRQADLKHAADDLVVVAYASAAELLHARVIRSLQAAARAVVSASNNLITTSHYAANKSRANNYHILTDEKAVSDLLPKLV  1195

Query  681   ISIRESRACPDDPLTQVELISACERFVLPCESLVRSSRSVAPTVSDPTTQAALDNSTSQLSGAIEVLRTCLARLTPLTRQMQVDGALARLARLAVEAAAIETGLKQGTLAPLPDEKIDDC  800
              +IR  R  PDDPL Q+ELI ACE  + PC  L RS RS+ PT+ D   Q+ALDNSTSQ+  A+E L+ CLAR +P+ RQ+Q+DGALA L R   EA  +E   K GTL  LPD+K++D
Sbjct  1196 SAIRFIRRDPDDPLAQLELIYACEHAIQPCGQLARSCRSMVPTIGDSAVQSALDNSTSQMVVAVETLKACLARASPIARQLQMDGALASLLRTVREAEELEDLAKAGTLTALPDDKVEDR  1315

Query  801   FNLLGTSVQDANVSTHELHSIVETMAQ-----QQRDMMSRSDVCGVPASMLATAIAGLVHATRGIVAHDAYDAADAGRSGAVGPSSDSTRLAVTQDTRQAVQLAYELVKAARDAR-----  910
             + +L  S++DA  +T+E+ S V+++ Q     Q  +     D  G  A+ LA A++ L+ +TR  +  +        G S A                  A QLAY+LV  AR
Sbjct  1316 YRILKVSIRDAQNATNEVTSAVDSLEQDYATPQSPNCDQGEDWLGSSANRLAGAMSDLLRSTRSCLVTEDRPLDSGGASTACA----------------AAQLAYQLVCEARKLEALQPS  1419

Query  911   FAYDDGQMEALAT---VVSHSEQLTVQLLDTLGRC-LRGLPGHREINEATNLIEKRREDLVQLSHEAPTRRVRWVEPSVYERAQSELTRAAVEFNQATGDLLSSYSPGAFRRTTRRFTGA  1026
              +  D Q + L +   V  + + +   L   L  C L+ LPG +E++EA++L+++RR+DLV+ + + PT     +E +  ER QSE T AAVEFNQAT DL S Y+P  FRR++ RF GA
Sbjct  1420 VSSPDQQADVLESNRNVAVNCKLIGENLNRALFECLLKALPGQKELSEASDLVDRRRKDLVRFA-DNPTVFEYPIEETRVERTQSEFTGAAVEFNQATADLTSCYTPSNFRRSSVRFAGA  1538

Query  1027  YDTLTVKGVDLCMSGVPPNEVPLPEPPPINRDLVDGLVNVSNHSYALMTEAGRVCARPDEQEIRDRFQTAARDVTESISQLLTICTSGVTAEQRDCEVALRRLEALRPLLENPNRPVNQK  1146
             Y+TL  KG+ L  +  P +         P    L++GLV VS+ S  ++ +A +VC++P+ + +R +   AAR VT+SIS LL++   SGV      DC+  LRRLEA+RPLLE P+RP NQ
Sbjct  1539 YETLVDKGLQLSRAKSPKD--------PTGPQLINGLVEVSDRSSEMLEDAKQVCSQPEAEPLRQKLHLAARSVTDSISHLLSVSVSGVVPGVADCDAELRRLEAMRPLLECPDRPTNQH  1650

Query  1147  TYCECVDEVARSITPLADSLRTMSNAAKEKHTQEFGAAVRQCSNSMCQLIEETAQAAYLIGLADPRSEPGRPSLVDPQLFMRSQREIQQVCDAICDPSITNRQ----IITLSTEMARSAK  1262
             TY  CV   A+S+ PL+D +R   +  +  +T  F + +R+ ++ +CQL+EET+QAAYL+G+A P SEPGR + VD  LF R QR+IQ +C A+  P +T R+    ++ L+ +M+ + +
Sbjct  1651 TYQLCVTSFAQSLVPLSDGIRGTLDGIRSHNTDVFTSNIRKVTDCLCQLVEETSQAAYLVGVAHPTSEPGRGTPVDIALFERLQRDIQSICRAMKSPEVTEREDRARVVNLANDMSANVR  1770

Query  1263  TLCEACSAVSAQTNNPDARHQLNALTRETMQSITALIQRRGNVSTAAAGSQLAEVNGEWDEANRQVTLANARGVSSNVARLVHMVTQGPQFIGQAARVTDEAREAQQPVCSAGLSSLNAA  1382
             +L +AC+A++AQ  + +AR +L  L  ++M+S++ LIQR                + +W +  R+VT+ N   V+ N+ RLV  +T   +F G+ AR++ EAR AQQP+C+AG + L +
Sbjct  1771 SLRDACNALAAQVTDQEARRKLQNLADDSMRSMSELIQR----------------SADWSDEGRRVTVTNIHTVNGNIDRLVSFITSISEFGGEPARISSEARNAQQPICAAGQACLESG  1874

Query  1383  QAVLRAAQTLITNARL-GQP-EQAFLSFSTASKELSESIKALAAAMREHAPGQLECQRVLQNINGLLQELQRAKMASMEGRLQPRRELNEEGFQKQLATCCRALLDAAPNVGRAARSEAE  1500
             + V+ A++ ++  A   GQP + AF +F+ AS++L+ + K L A + E  PGQ ECQR L NI  LL EL+R KMA M+G   PR + NEEGF KQL    RA+ D A  VGR A SEAE
Sbjct  1875 RGVILASKHMLQTAETNGQPSDTAFGAFTAASRDLTTNTKHLLALLNEQGPGQAECQRALLNIRRLLHELERDKMAMMDGVFTPRHDSNEEGFLKQLTLSARAVRDTATPVGRGATSEAE  1994

Query  1501  QLGHSVRVVDSYLPGITSSAIAAASRSPLSSTQLVYLEHASTILEAADQLVRVARDAGGNPRATHLHPHLDEAVRGLMESCEDLLTALDDVASRQGHVSTLIDTINRSLAQTEEIIQVPV  1620
             +LGH+VR +D  LPG+ S+ + AASR+P SS QL Y+EH  T+LE+ +QLV  A+ AGGNPR+  +   +D+ V+  ++SCE+L++A++ VAS+Q  ++ L+  +  S A  ++  + P
Sbjct  1995 RLGHAVRELDLLLPGLVSNTLGAASRAPNSSAQLTYVEHTRTVLESVEQLVSAAKQAGGNPRSDPIREAVDDGVKAQIDSCEELISAIEGVASQQSFIAKLMAVLEESRALVDKPGRAPY  2114

Query  1621  DARFADYQARLLRIARHMEQLTQAIQLRARQP--PSDGELAPLAHTLAQEYQEMCQTCKGAAATLPDARQADELRGAVRAVGMATAELVQATTAARIRAYDSHGDQQMLLSRNQGIA---  1735
             DA F DYQ+RLLR+ARHMEQ T+A    AR+P  P++ E+  L   LAQ+Y E+C   + A  TL D  +A++LR AV+ +G  T  L+ AT    +    S  D    LSR  G+
Sbjct  2115 DAHFTDYQSRLLRLARHMEQQTEATVRAARRPQEPTNQEMTGLVQNLAQDYAEICMISRDACETLRDPHEAEKLRWAVKNLGQTTRGLILAT----VNVAASRPDPS--LSRG-GVGAKP  2227

Query  1736  -----YLDTRAEALDSKLRDLIALLDTQGPNTQACLQSASTVSGIIADLDTTILFASSGTLHGPVHDDVDALLGPDDYRPPYALPGAGEAIADRGSRFGNREYPEEGFGTVR--------  1842
                   LD  A A++ +LR+LIALL+ QGP TQACLQ ASTVSGIIADLDTTILFASSGTLH P+ DD D       +R  Y+      AI+     FG RE  +  F ++R
Sbjct  2228 PACLRSLDFSAGAMNDRLRELIALLEVQGPGTQACLQGASTVSGIIADLDTTILFASSGTLHPPIRDDADVHQLEAQHR--YS------AISSGYDDFG-RE-AQNNFISIRTSSHVIMP  2337

Query  1843  --DSIVRTARALVDDTQSLVSGTGEDQTRLATTAHVAVERVTQLADVVKRGAAVIGPGQPDTQVEVLSACRDVATGLRDVLLAASQTPGRDASDPVHEQVRNNVQLTLSNIGALLQKVKT  1960
               +SI RTA ALV DT+SLV G GEDQ RLA +  +AV  ++QLADVVK+GA +IGPGQ D+QV +L A +D A  LR  LLAASQ  GR  +DPV ++++        S++  LL+ V +
Sbjct  2338 SSESIHRTADALVQDTRSLVIGAGEDQARLAGSTQMAVRNISQLADVVKQGAGLIGPGQSDSQVLILGAAKDTAVALRSTLLAASQVQGRPETDPVIKEMQMYESNVESSVAFLLKSVDS  2457
```

```
Query  1961  IADDENRGIQALVSAAKYCRDQAAQLAPRPEPESLLTPVSSRRSAPSVANV-SAVSGSSSLIARYLAPDDLARAASGPVQTAVSKAILAGNTQTQRDVLSTAATTRDVVTDLVSAATALL  2079
             I  +  RG +AL++ A  CR    QLA R    S +TP     S   + +  + +S +SSL++RYLAPD+LAR   GP++  V KA+ A +T+ Q D L +A T R  V DLV+A   L
Sbjct  2458  IDMESTRGTKALIATAHLCR----QLANR--MPSCVTPTGQAVSLNDLGSATTLLSTTSSLVSRYLAPDELARTTEGPLRMTVDKALGAASTRRQDDALISANTARHAVVDLVNACKNLQ  2571

Query  2080  RCPEAVTETRTACVASTKGLAEEFAGLLDALKAV--NSKPADHERIGNAARRIADLSHTLLSQLDNLRESPRLVMYFRASSPEWRDVAEKFIGRHVAYHHHYVPTYTIVGPGSTNRGLYV  2197
             R E   E R     + + +  E A LLDA+K V   + P D  R   +A +I  +S+ L+  +D +R++PRLVMYFR SSPEWRDVAEK++GRH+A+HH Y    + +    + R
Sbjct  2572  RQQEIKEELRKGGADTVRKVVLETAELLDAVKCVVAEANPNDINRASASAAKITAMSNHLMCLIDRMRDNPRLVMYFRVSSPEWRDVAEKYLGRHIAFHHQY---ESCIERDGSYRATTS  2688

Query  2198  RPICYPFRPQKAIPEQDEDRSGDEAEFRIATAIDQLKSIL--DCEPPSDPTILQSAHSVAVATQSLIHTARSIGLAPPSDAVDRARRAGTSHHGASLWRTELTVALATQDMCQLAQLCSA  2315
             +  C     Q  +       +G EA    +  ID    +L  D       +I Q    VA  TQ ++  AR++ LA   + V   + A TS   A+L R +L+VALAT+ +C+L +LCS
Sbjct  2689  QLSCL----QSQLDSTGRLLTGTEAALEASRRIDAALGLLEGDLYQSMQKSINQ---DVAHHTQPILSMARAVALA-TREFVQTVQLAFTSKTSANLGRLDLSVALATEQLCELTRLCSE  2800

Query  2316  VLQEENAHPDSKDLFGTSTRLVPSKERLLAAVRRVAAASAQLLMSAKSRRSKCSSADIQKLQVAGQLVKEATDRLSGVLHQAN--FTC--DSTGLYV--ITPMP--ATLQNVIETHSKIH  2427
                E        D      R++PS+ERLL+AVR+VA AS +LL + +S     +SA++  +Q A + VKE TD++S ++   +  F+     +GL V  +TPM   ++ +    T ++IH
Sbjct  2801  TRSEGPYTAPMDD----PIRVMPSRERLLSAVRQVATASGELLYAVQS-NVHITSANMIAVQTAARAVKEQTDKMSTMVRNKDPIFSHLPSQSGLEVSLLTPMSLVSSQEENTNTSTQIH  2915

Query  2428  SHQSELDVLQQRLAQL  2443
             + Q+ LD LQ  + +L
Sbjct  2916  TEQTVLDDLQNEVERL  2931
```
