# Supplementary material for: Identification of microRNA Genes in Three Opisthorchiids
Source: PLoS Negl Trop Dis. 2015 Apr 21;9(4):e0003680. doi: 10.1371/journal.pntd.0003680 (PMC4405270; doi:10.1371/journal.pntd.0003680)
Supplement: S9 Appendix — (PDF) [file pntd.0003680.s009.pdf]

S9 Appendix. Alignment of miR-190 precursors in the context of corresponding introns.

SS – secondary structure of csi-miR-190 pre-miRNA. sec. struc. – secondary structure. Mature miRNA sequences are in bold type and underlined.

```
                  Splice site         miR-190
sec. struc.                   ((.((((((((((.(((((.(((((.......(((((((((((....)
C. sinensis       GTAGGT~(47)~TGAGATATGTTTGGGTTACTTGGTGTGTGGTTTCCGATCGGAGCTACC
S. japonicum      GTAAAT~(56)~TGTGATATGTATGGGTTACTTGGTG-TCAGCGGATAACATGTTTTATT
S. mansoni        GTAAAT~(83)~TGTGATATGTATGGGTTACTTGGTG-TCAGCGGATAACATATTGTGTT
E. granulosus     GTATGT~(56)~TGAGATATGTTTGGGTTACTTGGTG----------------CTGTGCT
E. multilocularis GTATGT~(56)~TGAGATATGTTTGGGTTACTTGGTG----------------CTGTGCT
H. microstoma     GTTCGT~(53)~TGAGATATGTTTGGGTTACTTGGTG------------------CTGCC
T. solium         GTGTGT~(66)~TGAGATATGTTTGGGTTACTTGGTG----------------CTGTACT
                  **   *      ** ******* **************                   *

                                           miR-190*                    Splice site
sec. struc.       )))))))).)).......))))))))))))))))))))-----)..))
C. sinensis       TCTGGTCAGGTCTGTTGAACACCAGTGACCGAACATAT-----TCCCA~(2,424)~TTTCAG
S. japonicum      CGCCATTAAGCA----ATACACCAGTGACCAGACATATCCCTATCGCT~( 531 )~TTTTAG
S. mansoni        CGCTATTAAAAC------ACACCAGTGACCAGACATATCCCTATCGCA~(1,053)~TTTCAG
E. granulosus     C--TATCCTCTT------ACACCAGTGACCAAACATAT-----TCACA~(  90 )~TTCTAG
E. multilocularis C--TATCCTCTT------ACACCAGTGACCAAACATAT-----TCACA~(  89 )~TTCTAG
H. microstoma     TAT--TTAACTC----AAACACCAGTGACCGAACATAA-----TCCTA~( 224 )~TTGTAG
T. solium         C--TCTGCTCTT------ACACCAGTGACCAAACATAT-----TCACA~(  90 )~TTCTAG
                       *            ************  *****      **            **  **
```
