# Supplementary material for: Identification of microRNA Genes in Three Opisthorchiids
Source: PLoS Negl Trop Dis. 2015 Apr 21;9(4):e0003680. doi: 10.1371/journal.pntd.0003680 (PMC4405270; doi:10.1371/journal.pntd.0003680)

S1 Figure. Original photo of electrophoretograms.

Original photo of electrophoretogram of PCR products for Fig. 4D and Fig. 7E

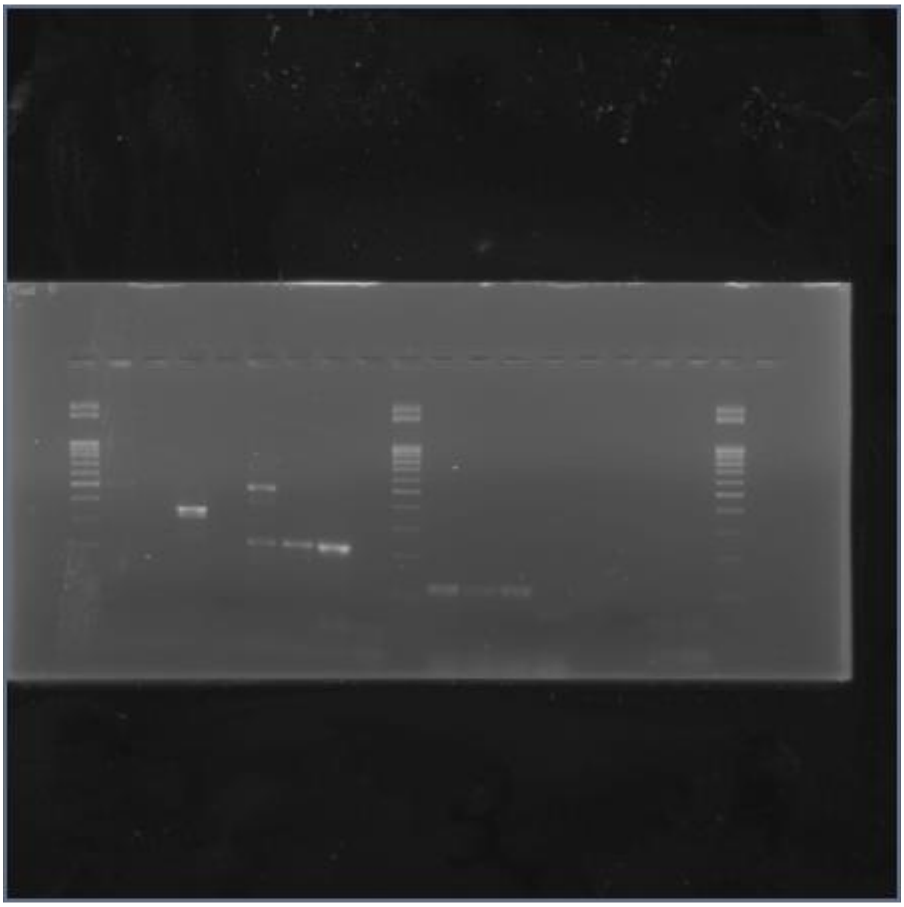

Original photo of electrophoretogram of PCR products for Fig. 4F

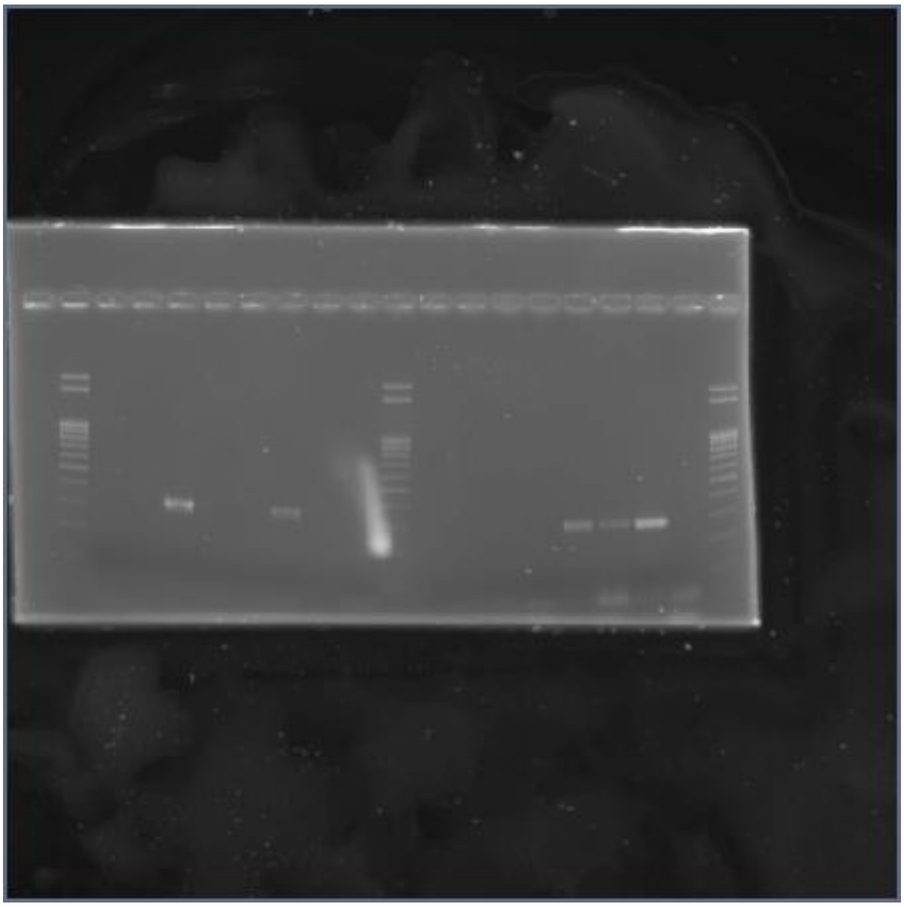

Original photo of electrophoretogram of PCR products for Fig. 4G

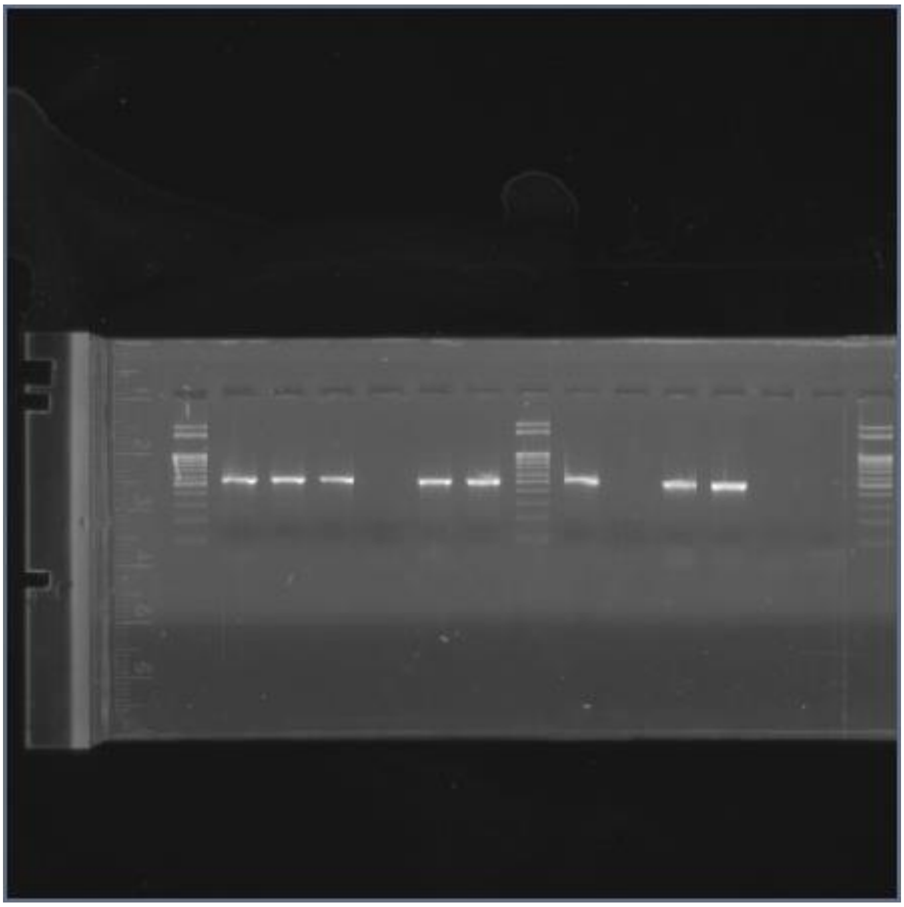

Supplement: S1 Fig — (PDF) [file pntd.0003680.s010.pdf]
