# Supplementary material for: Identification of microRNA Genes in Three Opisthorchiids
Source: PLoS Negl Trop Dis. 2015 Apr 21;9(4):e0003680. doi: 10.1371/journal.pntd.0003680 (PMC4405270; doi:10.1371/journal.pntd.0003680)
Supplement: S2 Fig — Mature miRNAs are red. Diferensis in sequences of miRNA precursors in red circles. (PDF) [file pntd.0003680.s011.pdf]

S2 Figure. Secondary structure of miR-71a, miR-2a, miR-2b, miR-2e precursors from *C. sinensis*, *O. felineus*, *O. viverrini*.

Mature miRNAs are red. Diferensis in sequences of miRNA precursors in red circles.

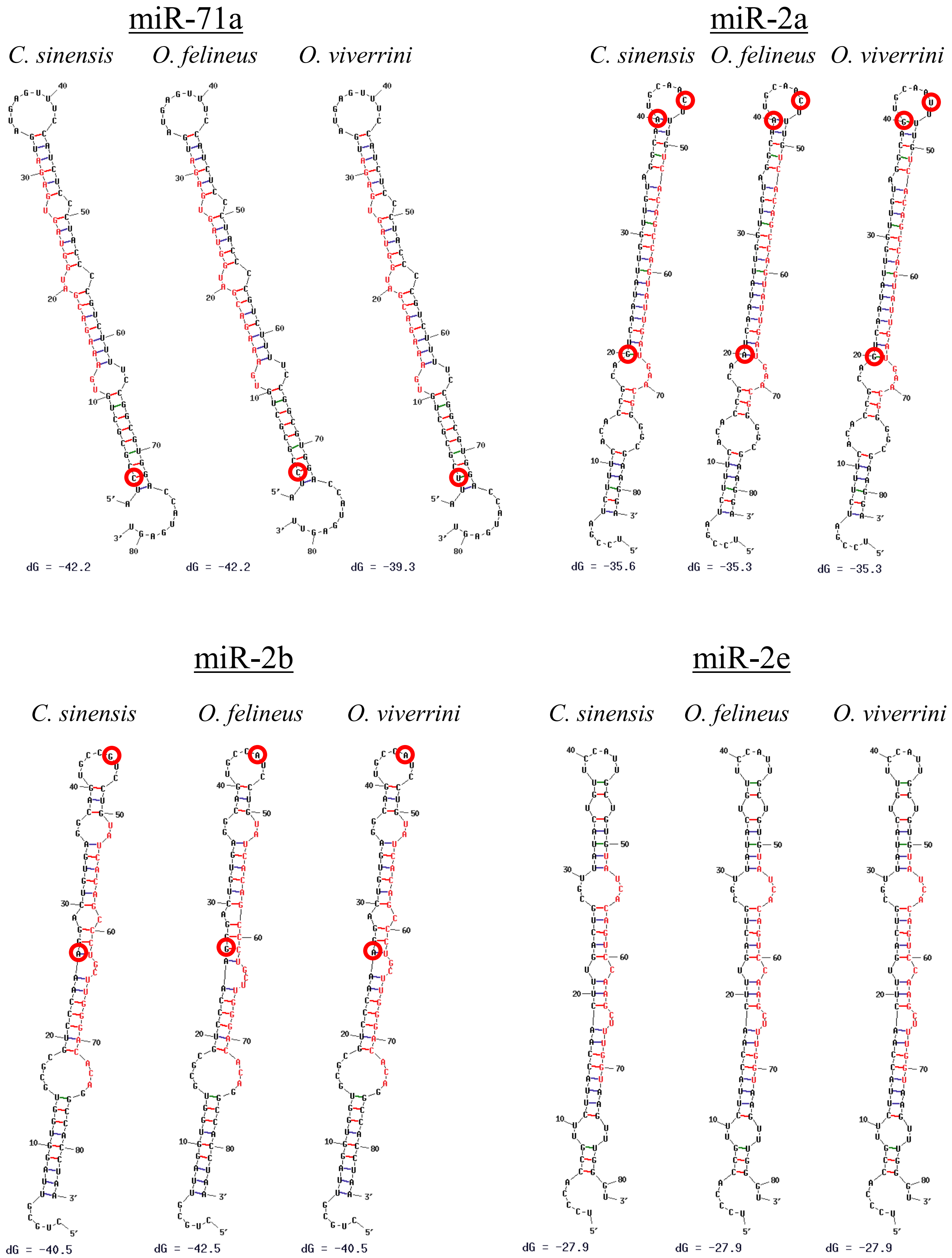

Mature miRNAs are red. Diferensis in sequences of miRNA precursors in red circles
